# Dynamic soil properties for soil health (DSP4SH) database 1.0 – Phase 1 and 2 datasets

**DOI:** 10.1016/j.dib.2024.110521

**Published:** 2024-05-12

**Authors:** Ekundayo Adeleke, Skye Wills, Tiffany Carter

**Affiliations:** USDA-Natural Resources Conservation Service, National Soil Survey Center, Hugh Hammond Bennett Center of Conservation and Soil Science, 1121 Lincoln Mall, Suite 382, Lincoln, NE 68508, USA

**Keywords:** Soil change, Ecological states, Land uses, Agricultural management systems, Soil enzymes, Soil organic carbon, Aggregate stability

## Abstract

The dynamic soil properties for soil health (DSP4SH) is a Science of Soil Health Initiative that was designed to collect, process, and publicize scientifically rigorous datasets that inform sound indicators and interpretations. The Soil and Plant Science Division of the United States Department of Agriculture - Natural Resources Conservation Service (USDA-NRCS) and university cooperators collected a suite of standardized soil health metrics across eight states (Oregon, Washington, Kansas, Minnesota, Illinois, Connecticut, North Carolina, and Texas) within five soil survey regions (Northwest, North Central, Northeast, Southeast, and South Central). The DSP4SH database provides a substantial dataset of soil health metrics assessed. The dataset is composed of dynamic soil properties (DSP) data collected from each management system or ecological state represented by one to three independent plot replicates. Each plot has a minimum of three pedons. Nine groups from the DSP4SH monitoring network provided datasets used in developing the database. The submitted data includes 37 laboratory measured parameters, 60 variables of layer/horizon descriptions, 41 variables for laboratory analysis conducted at the Kellogg Soil Survey laboratory, and 12 variables for the management systems. An additional 31 variables were developed for site or plot description. Additional variables were developed to normalize the dataset. In preparation for DSP assessment, all tables (except for dataset from KSSL lab) were categorized by management system or ecological state. The categories were business as usual (BAU), the reference condition (Ref) and the soil health management (SHM). The overarching goal of DSP4SH phase 1 and 2 dataset publication is to promote increased accessibility, further analysis of the data, and overall understanding of the benefits of surveying dynamic soil properties for soil health.

Specifications Table

**Table:** 

Subject	Agricultural Sciences (Soil Science), Environmental Science (Ecology), Data Science
Specific subject area	Dynamic soil properties; soil health indicators; soil survey; relational database
Type of data	Table, Raw, Code files, SQLite database
Data collection	The Science for Soil Health is an initiative established by Soil and Plant Science Division of the Natural Resources Conservation Service that would allow collaboration between NRCS staff and cooperators. This collaboration has resulted in generation of various scientific data. These data were acquired by using standard methods for describing and sampling soils in field and thereafter analyzing those samples in the laboratory. The specific and applicable methods utilized for collecting data are documented in the methods table of the database and the DSP4SH laboratory procedures. The instrumentation used to collect the data include elemental analyzers, manometers, mechanical rotating shakers, pH and electrical conductivity meters, microplate spectrophotometers, vortexers, incubators, and water baths.
Data source location	Eight US states: Oregon, Washington, Kansas, Minnesota, Illinois, Connecticut, North Carolina, and Texas
Data accessibility	Repository name: Ag data Commons Data identification number: 10.15482/USDA.ADC/25122323.v1 Direct URL to data: https://doi.org/10.15482/USDA.ADC/25122323.v1 Direct URL to code: https://github.com/alorthods/dsp4shdbv1-data-paper

## 1 Value of the Data

• The data for dynamic soil properties (aggregate stability, soil organic carbon, permanganate oxidizable carbon, autoclaved-citrate extractable protein, and soil respiration) can be used as a reference and baseline values for soil health indicators that can be linked to soil inherent properties and climate to enhance soil information.
• The data can also be used to assess the relationship and effectiveness of land use and management systems or conservation agriculture on overall soil health metrics. The data provides reference values that could be beneficial for state and transition models and other forms of related data modeling.
• These data provide a collection of dynamic soil properties and soil health indicators that can be used to standardize and update soil survey information.
• This dataset can be used to understand the influence of dynamic soil properties on soil health. These data would be beneficial to answering questions regarding the selection of land management and conservation practices that may be posed by researchers, soil scientist, ecologist, rangeland, and soil health specialists.

## 2 Background

Soil health is a foundational concept that describes the sustainability and productivity of a given soil. It is accepted that overall soil health is dependent on physical, biological, and chemical properties that are often dynamic in nature. Dynamic Soil Properties (DSPs) refer to the characteristics of soil that change on human timescale in response to both natural occurrences and human-induced activities, that includes agricultural and wildland management. These properties serve as markers for understanding how soil functions and undergoes transformations over time. The Science of Soil Health Initiative is a United States Department of Agriculture – Natural Resources Conservation Service (USDA-NRCS) effort to better understand soil health by assessing the variability of soil health indicators across the multiple soil types and land management practices. The USDA-NRCS has identified the following potential indicators that may provide key information to aid in the selection of best practices for conservation: bulk density, water stable aggregates, soil organic carbon (SOC), total nitrogen, texture, gravimetric moisture content, effervescence, electrical conductivity, pH (1-to-1, soil to water ratio), soil respiration, soil enzymes activities (β-glucosidase, N-acetyl-β-d-glucosaminidase, phosphomonoesterases (acid/alkaline phosphatase), and arylsulfatase), permanganate oxidizable carbon (POXC), and autoclaved-citrate extractable protein content. The datasets from the measurement of these indicators were collected in phases as these projects were completed. The phase 1 and 2 datasets were used for the development of first version of the database referred to as `dynamic soil properties for soil health database version 1.0`.

## 3 Data Description

**Fig. 1:**
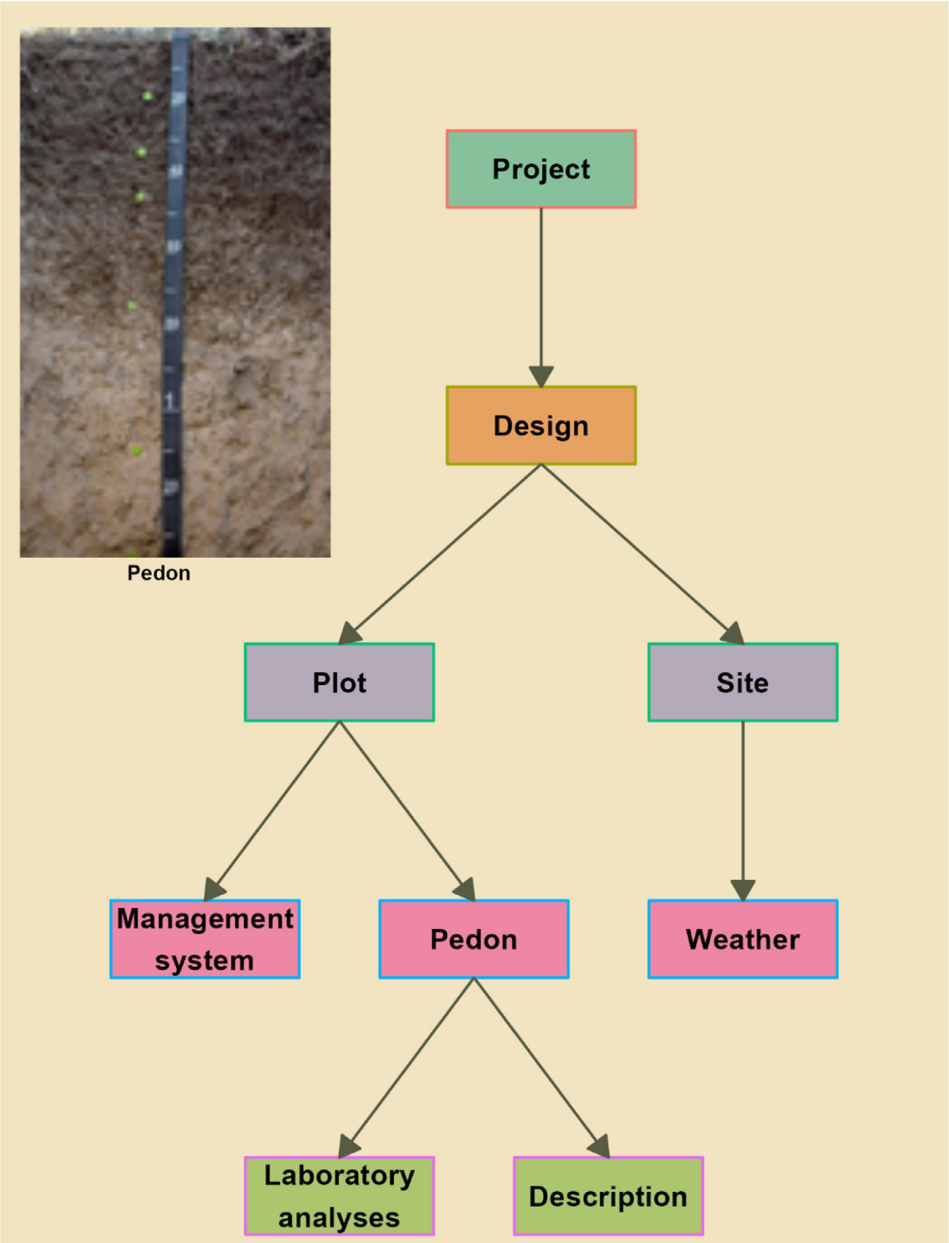
Generalized design of data collection. The flowchart above shows the five levels of the database, with data collected at the project level (dark green rectangle) linked to the dataset project design (dark orange rectangle) and linked to the dataset collected at the plot and site level (depicted as darkish purple color). The plot rectangle is linked to the management system and pedon level dataset (dark magenta color) while the site level dataset is linked to the weather dataset (dark magenta color). The pedon is linked to datasets of layer level laboratory analyses and descriptions (green rectangle) at the layer and horizon levels. The inset picture is a typical 2D representation of a pedon showing horizon and layers over 120 cm depth (from top to bottom). At least five fractions or peds were collected from each layer or horizon and consolidated as a single sample. Analyses were carried out for the layers (0–5 cm and 5–10 cm) and genetic horizons of all classification pedon. The number of pedons that were described and analyzed is 292. The number of horizons and layers analyzed is 1685.

The provided database includes ten datasets and four metadata tables [1]. There are five levels (Fig. 1) of the database. The project level allows for information and details on each project and management systems. The project level (with parent table named as *sourceproject*) is linked to the design level (with child table named as *projectdesign*) which provides details about the description of the project and linked to the site level (with table named as *plotoverview*). The site level has plot and site information that is linked to the pedon level. The pedon (referring to a 3-dimensional, representative sample of the soil, encompassing all its horizons and unique characteristics) level has the management system table (named as *dspplotmgt*) and pedon table (named as *pedon*) while the site table is linked to the weather table (named as *weather*). The pedon level is linked to the description level which is made up of pedon description (with table named as *layerdescription*) and laboratory analyses (with table named as *cooplabmst*) tables. An intermediate table designed to serve between the description and laboratory analyses is referred to as the *layerdesignation* table. The *horizon* is a section within the soil profile that has undergone distinct changes in its physical, chemical, or biological properties due to soil-forming processes, resulting in a recognizable and unique composition compared to other sections. The *layer* is defined as a fixed depth increment of the pedon.

The laboratory analyses tables provide details about the analyses conducted at the cooperator's laboratory and the Kellogg Soil Survey Laboratory – KSSL (with table named as *kssllabmst*).

## 4 Experimental Design, Materials and Methods

**Fig. 2:**
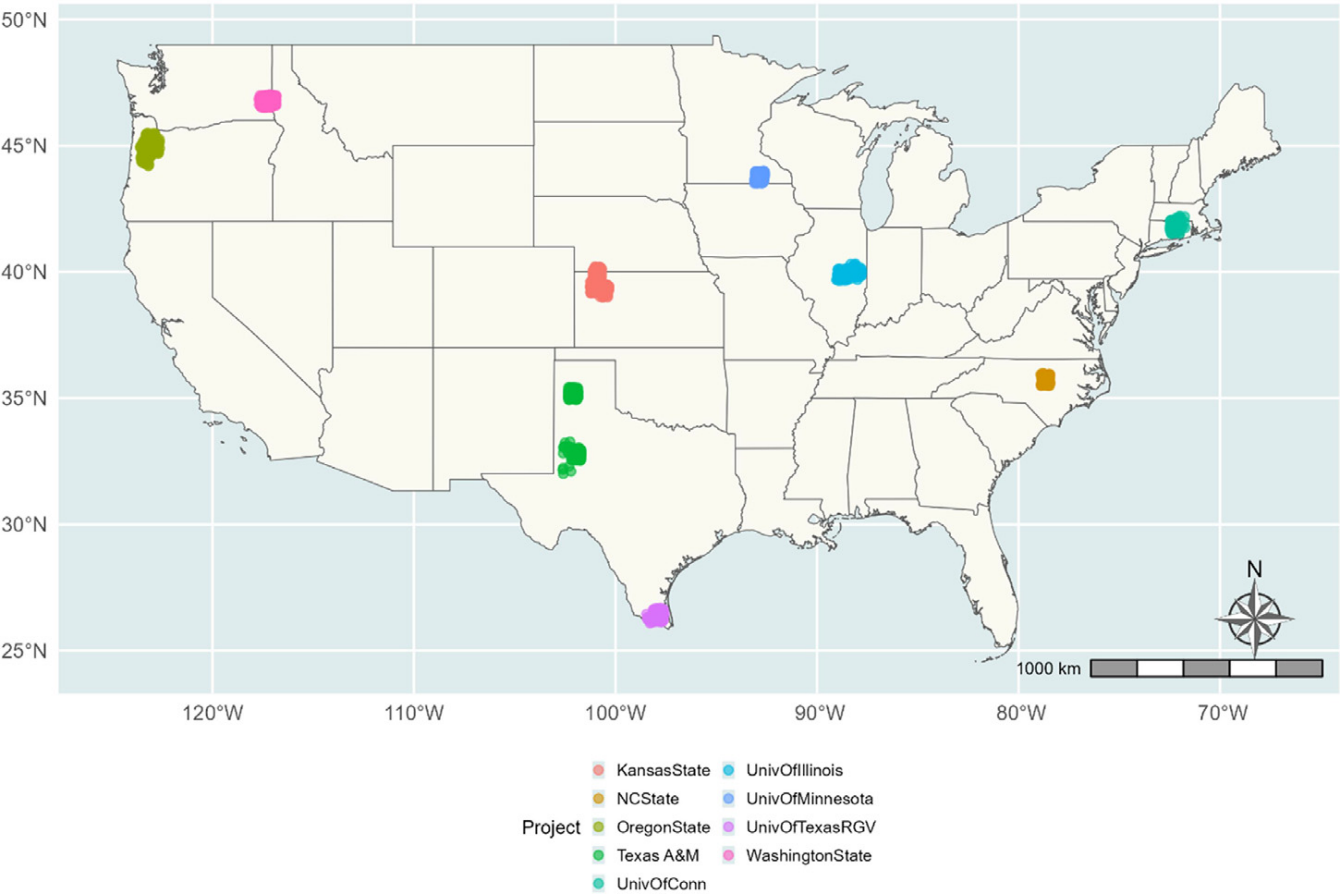
Distribution of DSP4SH projects. The DSP4SH projects were distributed across 9 different locations within US. Project locations are indicated by filled circles; different fill colors indicate different projects.

The acquisition of data began with the identification of sites based on various management differences (like tillage intensity, cover crops application and crop residue integration) and ecological states (like reference or cropland) that are relevant to soil health. The collection of information on ecological state and management history for each project (Fig. 2) was then carried out. This was followed by the identification and collection of the nearest climate station information for each site and plot information was also collected for each site. After locating a plot representing the management system, a characterization pedon and two satellite pedons were described per plot. For each pedon, each layer or pedogenetic horizon was described according to standard soil survey nomenclature using the Field Book for Describing and Sampling Soils[2]. Field description and observations included horizon nomenclature, field texture, soil color, redoximorphic features, concentrations, rock fragment volume, consistence, structure, roots, and pores information[2]. Field measurements included infiltration rates using single ring infiltrometer according to Ogden et al. [3,4]. Samples were collected from each layer at a fixed depth (0–5 cm and 5–10 cm) and thereafter by genetic horizon to 100 cm or bedrock. The collected samples were analyzed in the laboratory for biophysicochemical properties including bulk density, water stable aggregates, soil organic carbon (SOC), total nitrogen, texture, gravimetric moisture content, effervescence, electrical conductivity, pH (1-to-1, soil-to-water ratio), soil respiration, soil enzymes activities (β-glucosidase, N-acetyl-β-d-glucosaminidase, phosphomonoesterases (acid/alkaline phosphatase), and arylsulfatase), permanganate oxidizable carbon (POXC), and autoclaved-citrate extractable protein content [2].

Details of all laboratory methods used can be found in Standardized Methods for Selected Laboratory Procedures to Assess Soil Health. In brief, SOC was determined using the dry combustion method according to Nelson and Sommers (1996, Laboratory Method 1) [5]. Air dried soil sample was sieved to fine-earth fraction (<2 mm) and homogenized. The homogenized sample undergo further processing from <2 mm to <180 µm fraction using planetary ball mill and 80 mesh (≈180 µm). An aliquot of <180 µm sample was packed into tin foil that was weighed and analyzed for total carbon, nitrogen, and sulfur using an elemental analyzer. The soil sample was treated with hydrochloric acid and the evolved carbon dioxide (CO_2_) was measured manometrically as percentage of calcium carbonate (CaCO_3_). The SOC was calculated as the difference between total carbon and inorganic carbon. The mean weight diameter (MWD), an index used to relate aggregate stability to water stable aggregates [6] was determined using the Yoder wet sieving method according to Mikha and Rice (2004, Laboratory Method 2) [7]. For Yoder wet sieving method, the undisturbed soil clod subsample was sieved to <8 mm and air-dried. The air-dried sample was then placed in the top sieve of the nested sieves, pre-wetted for 10 mins before agitating for 5mins at 30 osc/min to disaggregate the soil through slaking. The mean weight diameter was then calculated as the sum of the fractional-mean weight diameter retained on each sieve. For water stable aggregates determined by KSSL wet aggregate stability method (Laboratory Method 3) [8], the undisturbed soil clod subsample processed to *a* < 8 mm fraction and air-dried was gently crushed to pass through 2 mm sieve and collected on a 1 mm sieve. The retention of the 2- to 1-mm air-dried sample was then measured on a 0.5 mm sieve after sample was submerged in distilled water overnight and thereafter agitated. The soil respiration was measured by conducting a 4-day incubation of the sample, with CO_2_ output determined by sealed chamber alkali trap respirometry (Laboratory Method 4) [9].

Soil samples were also quantified for five soil enzyme activities. The soil enzyme assays were quantified for β-glucosidase (BG) according to Eivazi and Tabatabai [10]; N-acetyl-β-d-glucosaminidase (NAG) according to Eivazi and Tabatabai [10]; phosphomonoesterases (acid phosphatase (ACP) and alkaline phosphatase (ALP)) according to Eivazi and Tabatabai [11]; and arylsulfatase (AS) according to Tabatabai and Bremner [12]. Briefly, 1 gram of soil sample was treated with modified universal buffer and p-nitrophenyl derivate substrate for each enzyme and then incubated for 1 hour at 37 °C. Calcium chloride (CaCl_2_) and THAM (pH 12) or NaOH solution was then added after incubation. Colorimetric determination of p-nitrophenol in an aliquot of the filtered solution was carried out and reported as potential enzyme activities (Laboratory Method 5). POXC was determined by quantifying reduction in the violet color intensity of 0.02 M KMnO_4_ (potassium permanganate) because of reaction with oxidizable C in soil according to Weil et al. (2003, Laboratory Method 6) [13]. Autoclaved-citrate extractable protein content was determined by extracting protein from samples using a neutral sodium citrate buffer solution. After clarification of the extract, the protein content was quantified using a bicinchoninic acid protein assay according to Walker (Laboratory Method 7) [14]. Soil texture was determined using the particle-size distribution analysis method (Gee and Or. 2002, Laboratory Method 8) [15]. The bulk density was determined using the field-state soil core method according to Grossman and Reinsch (2002, Laboratory Method 9) [16]. A metal sampler of known volume was driven into the soil. The metal cylinder was then removed from the soil extracting a known volume of the sample. Excess soil was removed from the edge of the cylinder and moist sample weight was recorded. The sample was then oven-dried at 110 °C to a constant weight and the bulk density calculated. The water content was determined using the gravimetric moisture content method (Laboratory Method 10) [8]. The effervescence was determined using the CaCO_3_ effervescence method (Laboratory Method 12) [8]. The electrical conductivity was measured with an electric conductivity meter (Laboratory Method 13). The pH was measured using a pH meter in a soil to water (1:1 w/v) solution (Laboratory Method 14) [8].

**Fig. 3:**
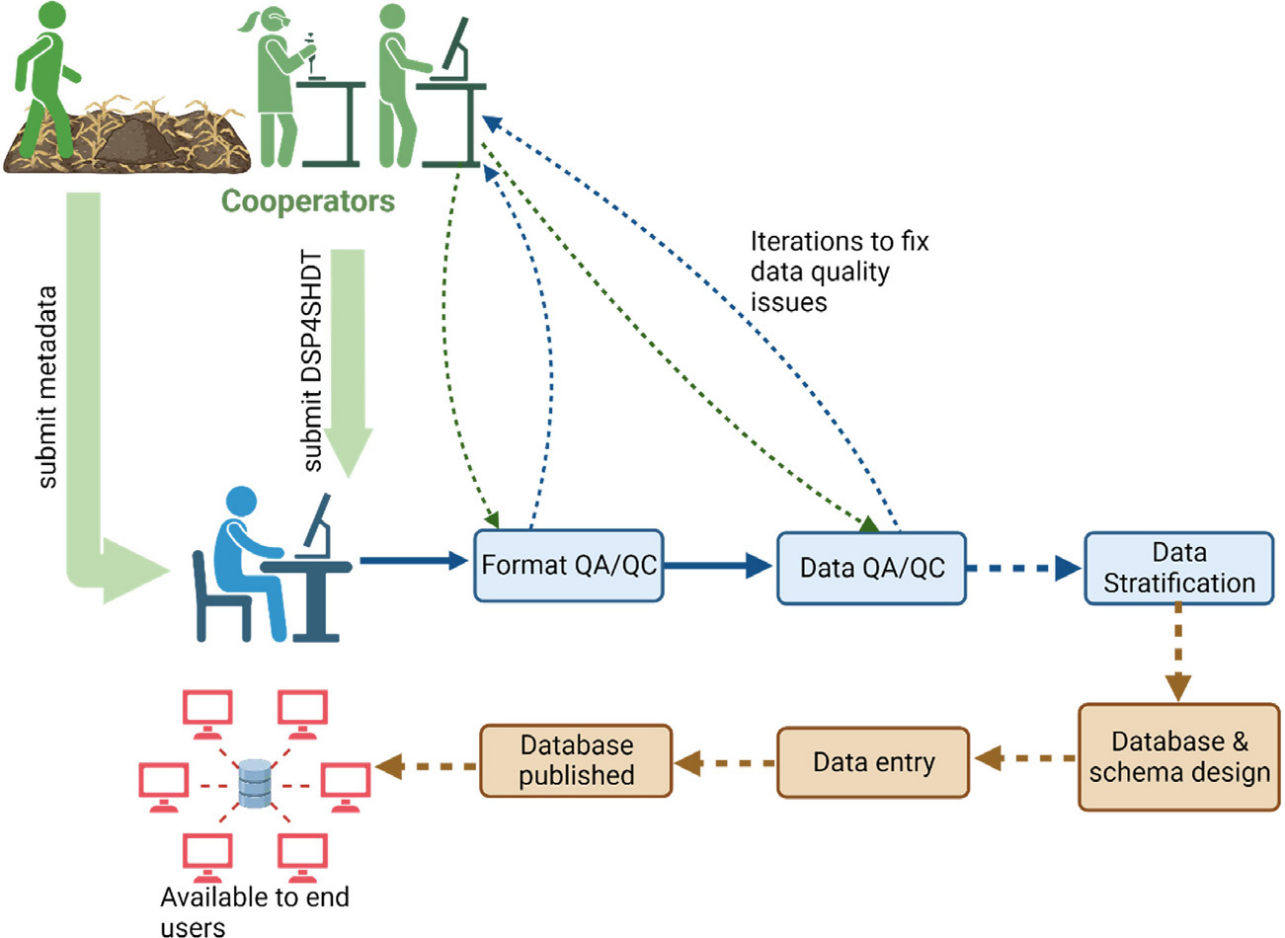
Generalized pipeline of processing DSP4SH data. The cooperators submitted the data template (DSP4SHDT) accompanied by the metadata to the DSP4SH data management team. This was followed by the template formatting for QA/QC, data QA/QC and then categorization of management systems. The database and schema were then designed. Then, data was entered, and database was made available to end users. The green color depicts the cooperators’ activities, the blue color depicts the DSP4SH data management team activities, the brown color depicts the DSP4SH database development activities, and the red color signifies activities of the end users. The solid lines indicate progression in the direction of the arrows while the dotted lines indicate progressions with iteration.

The pipeline for processing the collected DSP4SH data started with cooperator submission of data template (DSP4SHDT) and metadata to the DSP4SH project management team (Fig. 3). The DSP4SHDT, a template used for data transfer, is a spreadsheet that has 10 tabs. The tabs in the DSP4SHDT are “Instructions” – used to provide general information on how to use the template, “TablesOverview” – serves as a data dictionary (with brief detail on table name, column name, method and explanation, unit of measurement, and data type) that is used to provide the explanation of tables and fields within each table, “Project_design” – provides the space to enter description of the project design, “Project-Plot_overview” – provides the space to enter data on project at the plot level, “Map_photo_schematic” – provides description and details about where to populate plot maps, “Weather” - the space to enter details about the climate station closest to each site, “Pedon_entry” - the spreadsheet where details on each pedon can be added, “Layer_description” – spreadsheet where all data collected for all layers/horizon sampled can be added, “Layer_lab_msmt” – spreadsheet where all laboratory data can be added, and “ChoiceLists” – is a metadata for all lists inputted within previous tabs.

The submitted data template was then processed for format QA/QC and data QA/QC while iterations were performed in conjunction with the cooperators to resolve any data quality issues before stratification of the data. The database schema, entity relationship model and entity relationship diagram were then developed for the database. The data were imported from the DSP4SHDT, the database was created and uploaded with the data using custom-built R script. The SQLite database was then published as a data product that is available to the end users. This open access database is available to scientists, researchers, policy makers, and the public.

**Fig. 4:**
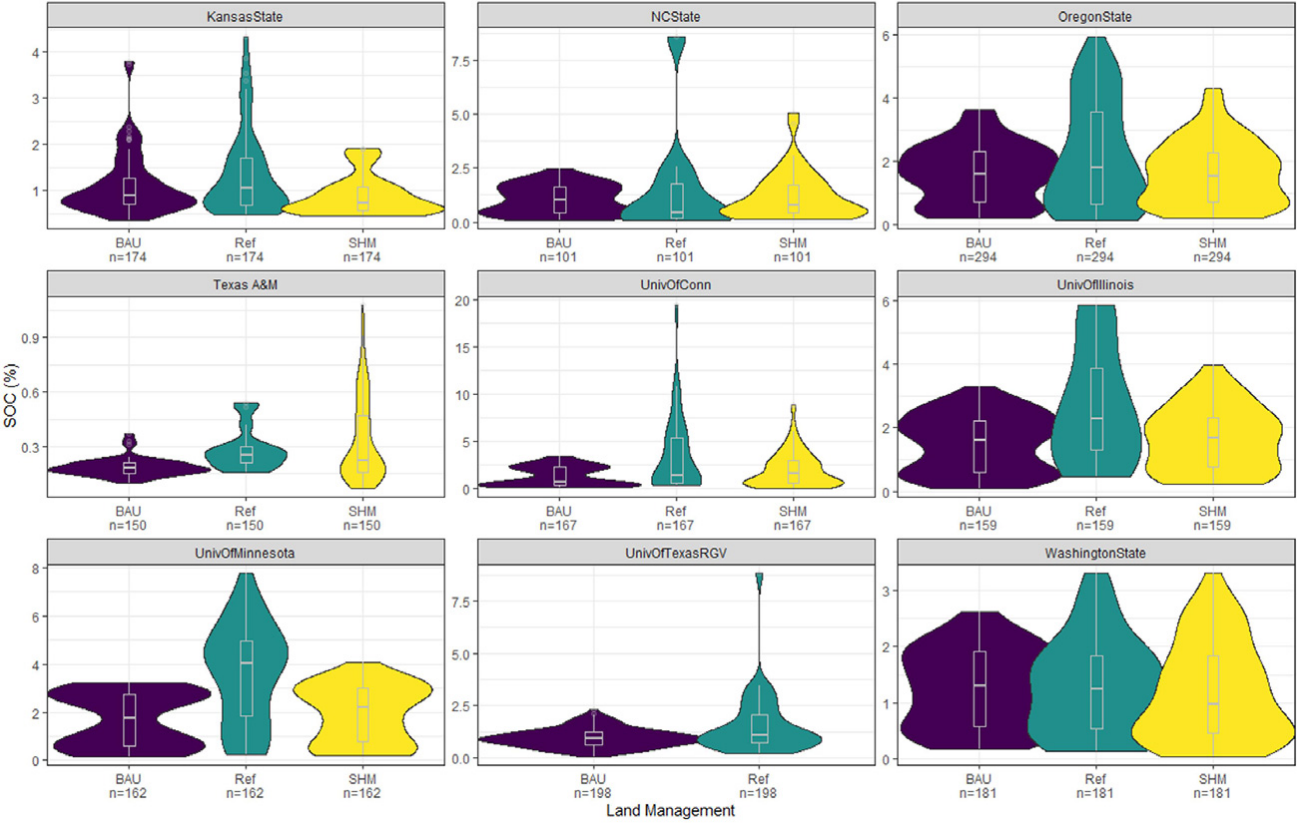
Distribution of the SOC vs. land management faceted by projects. The figure shows comparison of SOC and land management system across project and within project. Also shown in the figure is the count (n) of each classification (BAU, Ref or SHM) and measurement of SOC. The violin plot shows the spread of the SOC observations while the boxplot inset shows the central tendency and interquartile range of the SOC measurement by mass. Each observation of SOC represents SOC measurement for each layer by depth (cm).

The database can be accessed, and tables combined to analyze the soil health metrics and ecological or management systems provided in the database. The visualization of the distribution of the SOC versus land management faceted by the project provided in Fig. 4 shows one of many possibilities of harnessing what is available in the database. The SOC is available in the `cooplabmst` entity which was then joined with the `pedon` entity and then `dspplotmgt` entity. The generation of these entities was used to generate a plot of SOC vs land management in Fig. 4.

**Fig. 5:**
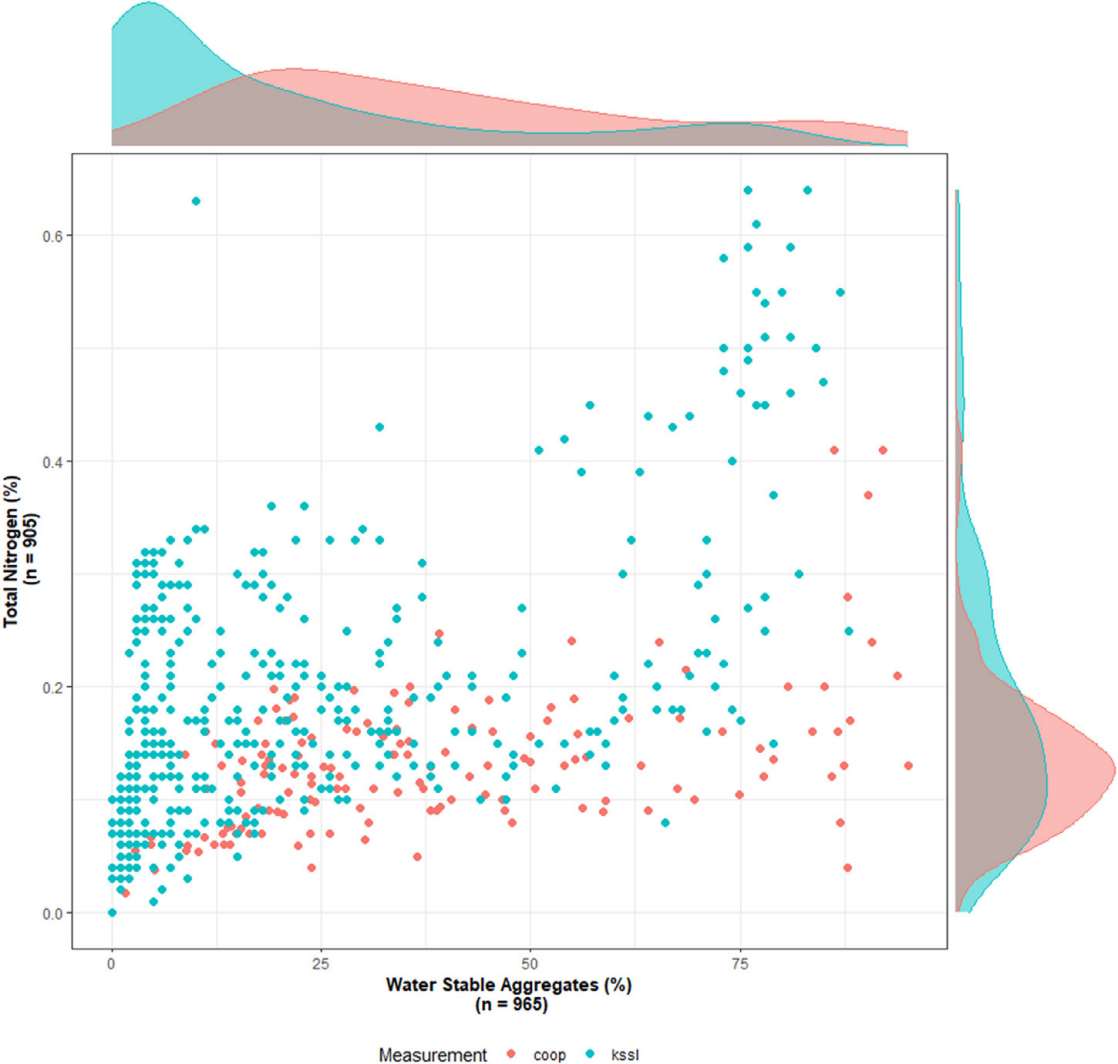
Plot of total nitrogen (percent) vs water stable aggregates (percent) by laboratory measurements with marginal density plot for each variable.

The combination of query and data manipulation can allow for visual exploration of the data. For example, the variables like total nitrogen (available as `TN_pct`) and water aggregate stability (available as `KSSL_WSA`) from the cooperators’ laboratory measurement entity, `cooplabmst` and KSSL measurement entity `kssllabmst` were queried from the database. The two variables were then filtered and joined to create a plot of total nitrogen vs water stable aggregates as shown in Fig. 5.

**Fig. 6:**
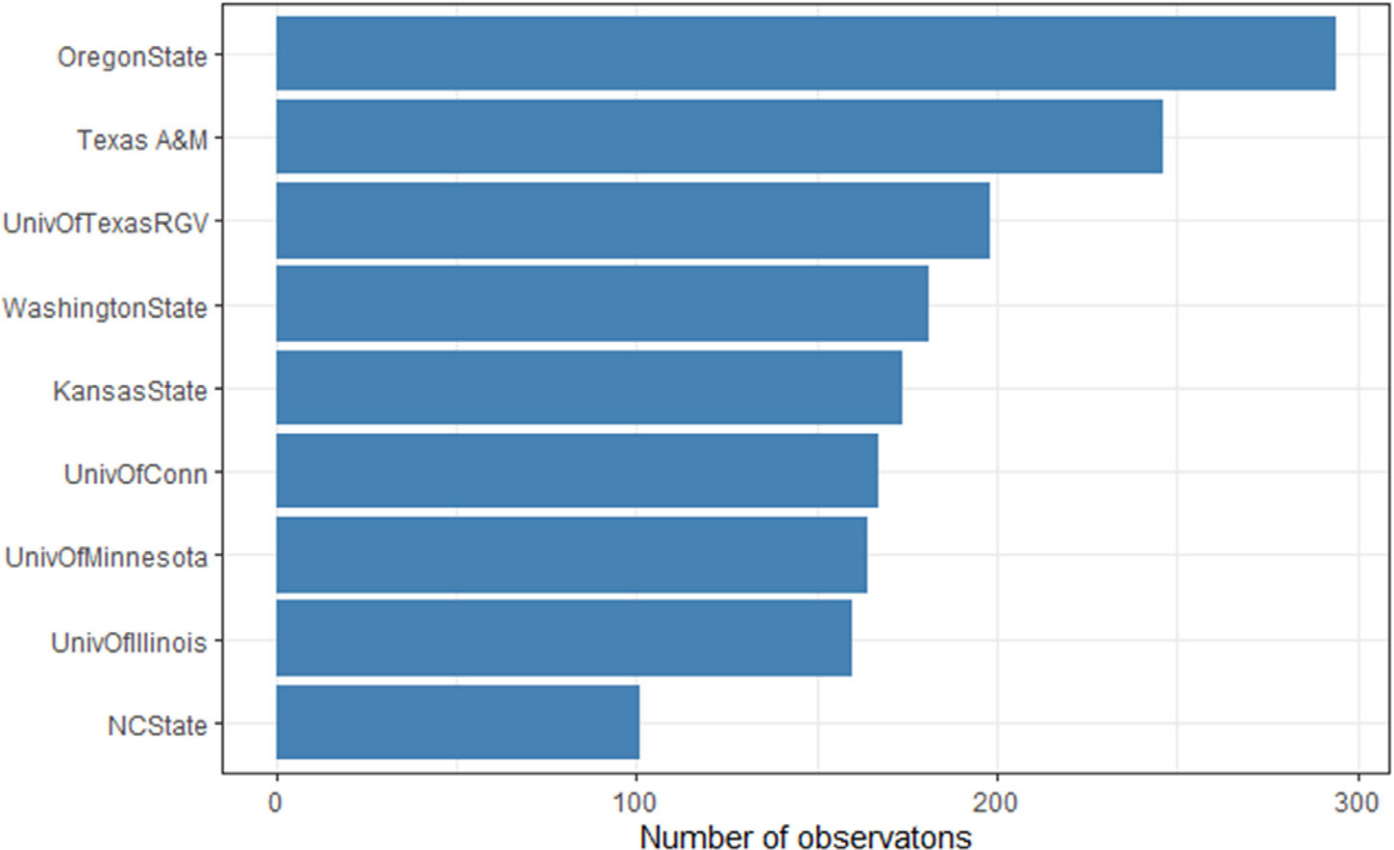
The number of observations within the laboratory measurement (cooplabmst) table.

The number of observations within the `cooplabmst` was also explored and shown in Fig. 6. This was achieved by a combination of the `dspplotmgt` entity which was combined with the `pedon` entity and then `cooplabmst` entity. The *OregonState* project had 294 observations, *Texas A&M* project had 246 observations, *UnivOfTexasRGV* project had 198 observations, *WashingtonState* project had 181 observations, *KansasState* project had 174 observations, *UnivOfConn* project had 167 observations, *UnivOfMinnesota* project had 164 observations, *UnivOfIllinois* project had 160 observations, and *NCState* project had 101 observations. This gave rise to a total of 1685 observations across all cooperators’ lab measurements.

## Limitations

There are limited data that combine assessment of dynamic soil properties and ecological state or management systems in the US. Despite the database accomplishing this distinct achievement, the restricted availability of these data across different regions of the US results in significant data gaps essential for developing soil health models under varying ecological conditions and management strategies. Subsequently, enhancing data collection efforts in underrepresented states across each region, focusing on a variety of soil types, will effectively bridge these gaps.

As previously noted, the database amalgamates soil characterization and management data, that can serve as a reference value for future studies. However, this data is not designed for use as a long-term soil monitoring data. In the absence of additional data, this limitation hampers the understanding of temporal soil changes under similar environmental conditions.

The combination of the data from this database with climate data for each location will enhance the modelling and analyses for ecological system functions.

## Ethics Statement

The authors declare that the presented data conforms with the requirements of publication in this journal and does not involve human subjects, animal experiments or data collected from social media platforms.

## CRediT authorship contribution statement

**Ekundayo Adeleke:** Conceptualization, Writing – original draft, Methodology, Data curation, Formal analysis, Visualization. **Skye Wills:** Conceptualization, Writing – review & editing, Formal analysis, Supervision. **Tiffany Carter:** Writing – review & editing, Supervision.

## Data Availability

Dynamic Soil Properties for Soil Health (DSP4SH) Database 1.0 – Phase 1 and 2 datasets (Original data) (Ag Data Commons)

## Appendix Supplementary materials

Image, application 1
