# Supplementary material for: Dynamic soil properties for soil health (DSP4SH) database 1.0 – Phase 1 and 2 datasets
Source: Data Brief. 2024 May 12;54:110521. doi: 10.1016/j.dib.2024.110521 (PMC11111840; doi:10.1016/j.dib.2024.110521)
Supplement: Supplementary file 1 [file mmc1.docx]

**Standardized Methods for Selected Laboratory Procedures to Assess Soil Health**

**Table of Contents**

[**Table of Method Index** 2](#_Toc152658915)

[**Introduction** 3](#_Toc152658916)

[**Sample Collection and Preparation** 3](#_Toc152658917)

[**Laboratory Method 1: Soil Organic Carbon (SOC)** 11](#_Toc152658918)

[**Laboratory Method 1a: Total Carbon, Dry Combustion Method** 11](#_Toc152658919)

[**Laboratory Method 1b: Soil Inorganic Carbon, Calcium Carbonate** 14](#_Toc152658920)

[**Laboratory Method 1c: Soil Organic Carbon, Loss on Ignition** 17](#_Toc152658921)

[**Laboratory Method 2: Yoder Wet Sieving Method** 22](#_Toc152658922)

[**Laboratory Method 3: KSSL Wet Aggregate Stability** 29](#_Toc152658923)

[**Laboratory Method 4: CO2 Respired, 4-Day Incubation** 33](#_Toc152658924)

[**Laboratory Method 5: Soil Enzymes** 38](#_Toc152658925)

[**ß-Glucosidase** 38](#_Toc152658926)

[**Acid Phosphatase** 43](#_Toc152658927)

[**Alkaline Phosphatase** 48](#_Toc152658928)

[**Arylsulfatase** 54](#_Toc152658929)

[**N-acetyl-B-glucoaminidase** 59](#_Toc152658930)

[**Laboratory Method 6: Permanganate Oxidizable C (Active Carbon)** 65](#_Toc152658931)

[**Laboratory Method 7: Autoclaved Citrate Extractable (ACE) Protein Content** 71](#_Toc152658932)

[**Laboratory Method 8: Particle-Size Distribution Analysis** 80](#_Toc152658933)

[**Laboratory Method 9: Bulk Density** 86](#_Toc152658934)

[**Laboratory Method 10: Water Content** 88](#_Toc152658935)

[**Laboratory Method 11: Total Nitrogen (as part of total carbon, total nitrogen, and Sulfur analysis)** 90](#_Toc152658936)

[**Laboratory Method 12: Effervescence** 94](#_Toc152658937)

[**Laboratory Method 13: Electrical Conductivity** 95](#_Toc152658938)

[**Laboratory Method 14: Hydrogen-Ion Activity (pH)** 97](#_Toc152658939)

[**Laboratory Method 15: Community Structure, Phospholipid Fatty Acids (PLFA)** 99](#_Toc152658940)

[**References** 114](#_Toc152658941)

# **Table of Method Index**

| **Method Code** | **Method Description** | **Method Name** | **Unit of measure** |
| --- | --- | --- | --- |
| Sand_pct | Total Sand (%) | PSDA, Air-dry, <2 mm Particles | % |
| Silt_pct | Total Silt (%) |  | % |
| Clay_pct | Total Clay (%) |  | % |
| Texture | The USDA textural class or subclass of the fine-earth fraction (≤2mm) that is determined in the laboratory |  |  |
| Bulk_Density | Field bulk density, Db_f_ (g cm-3) | Bulk Density, Soil Core | g cm-3 |
| Water_Content | Gravimetric water content | Air-dry to oven-dry ratio | % |
| TC_pct | Total carbon (%), measured by combustion as all organic and inroganic carbon, including those found in carbonate minerals. |  | % |
| Effervescence | Check for presence of carbonates | Effervescence, 1N HCl |  |
| SOC_est | estimated organic carbon based on sample pretreatment to remove CaCO_3_ | Modified Walkley-Black Method | % |
| SOC_pct | Organic carbon (%), measured through combustion and corrected for CaCO_3_ as inroganic carbon | Dry combustion method | % |
| SOC_loi | Soil organic content (%) calulated as the difference between 100 and mineral content (which is the ratio of the residual weight after ignition to the oven-dried weight) | Mineral Content, Loss on Ignition | % |
| TN_pct | Total nitrogen (%), measured with combustion | Combustion | % |
| KSSL_WSA | Water stable aggregate (%) measured using KSSL-based laboratory method 3 (Additional DSP4SH Methods, DSP4SH_LabProcedures pp. 13–16 ) | KSSL Wet Aggregate Stability | % |
| Yoder_AggStab_MWD | Mean weight diameter measured using Yoder/ARS-based laboratory method- (DSP4SH_LabProcedures and App. 2 (Stott 2019 - Soil Health Tech Note)) | Yoder-based Wet Sieving Method | mm |
| Yoder_AggStab_TotalWS | Total water stable soil aggregate fraction (%) Yoder/ARS method (DSP4SH_LabProcedures and Stott 2019 - Soil Health Tech Note) | Yoder-based Wet Sieving Method | mm |
| Yoder_WS_2-8 | Water stable soil aggregate fraction ranging from 2mm (2000µm) - 8mm (8000µm) diameter size | Yoder-based Wet Sieving Method | mm |
| Yoder_WS_pt5-2 | Water stable soil aggregate fraction ranging from 0.5mm (500µm) - 2mm (2000µm) diameter size | Yoder-based Wet Sieving Method | mm |
| Yoder_WS_250-500 | Water stable soil aggregate fraction ranging from 250µm - 500µm diameter size | Yoder-based Wet Sieving Method | µm |
| Yoder_WS_53-250 | Water stable soil aggregate fraction ranging from 53µm - 250µm diameter size | Yoder-based Wet Sieving Method | µm |
| EC | Electrical conductivity | Electric conductivity | dS/m |
| pH | Soil-water suspension (1:1). The pH of a sample measured in distilled water at a 1:1 soil:solution ratio | pH, H2O |  |
| Soil_Respiration | Carbon dioxide respiration (mg), 4-day incubation, measured using CASH-based laboratory method | CASH-based soil respiration method | mg CO2 |
| Bglucosidase | Soil enzyme - B-glucosidase measured using laboratory method | Enzymes | mg.Kg^-1^hr^-1^ |
| Bglucosaminidase | Soil enzyme - N-acetyl-B-glucosaminidase measured using laboratory method | Enzymes | mg.Kg^-1^hr^-1^ |
| Alkaline Phosphatase | Soil enzyme - Alkaline phosphatase measured using laboratory method | Enzymes | mg.Kg^-1^hr^-1^ |
| Acid Phosphatase | Soil enzyme - Acid phosphatase measured using laboratory method | Enzymes | mg.Kg^-1^hr^-1^ |
| **Method Code** | **Method Description** | **Method Name** | **Unit of measure** |
| Phosphodiesterase | Soil enzyme - Phosphodiesterase measured using laboratory method | Enzymes | mg.Kg^-1^hr^-1^ |
| Arylsulfatase | Soil enzyme - Arylsulfatase measured using laboratory method | Enzymes | mg.Kg^-1^hr^-1^ |
| POX_C | Permanganate oxidizable carbon measured using KSSL-based laboratory method | Permanganate oxidizable carbon | mg.kg^-1^ |
| ACE | Bioavailable organic nitrogen measured using laboratory method | Autoclavable Citrate Extractable Protein content | g.kg^-1^ |
| KSSL_LabSampleNo | Unique sample ID generated and added by NSSC staff (equivalent to natural_key on KSSL table) |  |  |
| PLFA | link individual samples to SHAC PLFA samples | Buyer and Sasser high-throughput method | pmol/g soil |

# **Introduction**

There is growing interest within government agencies, scientific communities, and public to assess soil health using soil health indicators as tool for land management. Conventional soil surveys have often provided data and information on the inherent properties of the soil but not much so about soil properties that change because of land use and management practices on human time scale. The Soil and Plant Science Division of National Resource Conservation Service (NRCS) under the Soil health initiative has recommended laboratory procedures to assess soil health through the project titled “Dynamic Soil Properties for Soil Health (DSP4SH). This project includes agreement with several universities that use common protocol and procedures that would evaluate proposed soil health metrics.

This document is an updated version of the laboratory protocol for the Science of Soil Health Initiative. The methods are internal recommended procedures by NRCS to assess soil health. They are written for ease of understanding and implementation of protocols from sampling in the field to analyses in laboratory.

# **Sample Collection and Preparation**

**Application**

The coordination of sampling-site selection and sample collection are critical part of quality assurance process that leads to successful laboratory characterization of soil samples. Successful laboratory analysis of soil starts with appropriate soil sampling (SSIR No. 42, 2014). Sample collection and preparation requires planning and coordination ahead of visit to the field or plot. In the field, the selection of site, description of site and soil pedon, and careful sample collection is required. A complete description of soil site not only provide context to the soil properties but also help with the evaluation and interpretation of laboratory analytical results (Patterson, 1993). The aim of collecting soil samples is to garner data on specific soil types and their properties. Sampling allows us to examine the factors of these soil properties with satisfactory accuracy while minimizing costs. Moreover, subsampling offers the opportunity to estimate a subset of the larger sampling unit without the need to measure it in its entirety. Therefore, due to the heterogeneity of soil and limitation of sample size, the amount of soil material needs to be adequate and thoroughly mixed to derive a representative sample. The representative samples can be collected from a layer or horizon of each pedon as bulk material, known volume (bulk density) or undisturbed material (aggregate stability). There are several possibilities to collect and prepare soil samples subject to physical, chemical, and biological analyses to be conducted. Therefore, the intent herein is not to detail all possibilities about sampling soil but to describe the main procedures to prepare samples for laboratory analyses used in assessing soil health. These laboratory analyses are often conducted on air-dry, and fine earth (<2 mm) fractions. Air-dry soil has more relatively stable moisture content making it easier to process the soil than the field moist sample. Most chemical and physical requires sieving the air-dry soil sample through a 2 mm sieve. For some other standard air-dry analyses, the <2 mm fraction is further processed (≈ 180 µm) to be in accordance with the standard method (e.g., to meet sample prep method of total C, N, S equipment). Moreover, some standard air-dry analyses require whole-soil samples.

**Summary of Method**

This method obtains representative samples of bulk material from every horizon where the samples have been described and undisturbed samples were obtained from the corresponding horizon. Also, known volume sampling will allow determination of bulk density for every layer. For most of the standard chemical, and physical analyses, the field sample is air-dried, crushed, and sieved to <2 mm. For some other standard analyses, air-dry samples may be prepared as a whole soil or processed to a finer (≈ 180 µm) earth fraction than <2 mm. Weight of the soil are taken as 2- to 5-mm fractions or 5- to 20-mm fractions that can then be crushed and sieved to <2 mm before chemical and physical standard analyses.

**Interferences**

- Soil variability and sample size are interferences to sample collection and preparation.
- Enough and additional soil samples may need to be collected to accurately measure the amount of rock fragments in soil.
- External sources of interferences could serve as obstacle to the sampling process (e.g., weather, wet terrain, steepness, and large rock fragments).
- Avoid samples drying out. Dried out samples could affect processing of soil, some sample analyses, and microbial biomass or activities.
- Make note of cores or probes not full due to compaction during sampling and adjust depth of horizon accordingly.
- Avoid prolong period between collection of samples and biological sample analyses.

**A. Soil sample collection:**

**Safety**

If the sampling pit is greater than 125cm (5 ft), create an opening or sloped end to one side of the pit to avoid entrapment. If using acetone for undisturbed clods, it should be used downwind from the pit to avoid collection of the fumes in bottom of the pit. For hand-dug and backhoe pits, care should be taken to avoid sharp edges of excavation tool. Proper clothing and hat should be used when working with excavation machinery.

**Equipment**

- Plastic bags, for mixed soil samples
- Zip-lock plastic freezer bags, for biological samples
- Tags, for bagged samples
- Plastic bags, for bulk density and thin section clods
- Core boxes, to transport cores from hydraulic probe
- Stapler, with staples
- Felt markers, permanent
- Sampling pans
- Sampling knives
- Chisel
- Rock hammer
- Nails
- Measuring tape
- Sieves (3-inch and 20-mm)
- Plastic sheets
- Canvas tarp
- Camera (for taking pictures)
- Frame, 50 cm x 50 cm
- Garden clippers
- Pruning shears
- Bucket
- Scale, 100-lb capacity, for rock fragments
- Electronic balance, ±0.01 g sensitivity, for weighing roots and plant residue
- Cooler, with ice or gel packs, for biological samples
- Containers, with screw caps, acid-washed, for water samples
- Gloves, plastic, powderless
- Bulk density equipment - if natural clods are not appropriate technique, e.g.,

bulk density cores

- First-aid kit
- Dust mask
- Hardhat
- Hand lens

**Reagents**

- Acetone
- Water, in spray bottle
- 1 N HCl
- Material Safety Data Sheets (MSDS)

**Procedures**

The DSP project is ideally designed to accommodate soil and ecological/management systems with multi-scale replication that would capture the variability within the field and improve soil survey information. The combination of one or two soil with two or three management systems (one of that should be native or represent a reference condition) will typically require three sites/field that includes all the combinations. After completing the soil and geomorphic description, describe and collect samples from the representative pedons of the DSP plot.

At each field/site, collect management information and field-level soil health indicators (may include site coordinates, ecological state and transitions, climate). At each plot, locate three independent pedons that represent the variation in topography and yield, collect the location coordinates (using standard world geodetic system (WGS) 1984), collect location specific field measurements and soil descriptions (including horizon designation, structure, texture, coarse fragments, colors, redox features). Pedon samples can be taken from the previously described pit. Sample pedon using sampling core or large probe at five locations within 1 m^2^.

The quantity of bulk samples collected from each horizon will depend on planned soil analyses. The typical sample size submitted to KSSL for laboratory characterization project (one per soil x management system combination is expected) is approximately 2 kg.

**Collecting and Handling of Soil Samples**

Ensure the soil sample collection bags are properly labeled. Collect whole-profile soil samples from a pedon of at least 1 m (100 cm) depth (or to the parent material for shallower soils). The sampling of pedon should be a combination of fixed-depth and horizon sampling scheme. The pedon should utilize fixed-depth sampling at 0-5, 5-10 cm and the rest of the pedon should be sampled according to genetic horizon up to 100 cm (or deeper/shallower accordingly).

If sampling core has been used, measure the length (cm) of each horizon and record it. Account for compaction if the soil is not full and adjust the horizon depth accordingly.

Keep samples collected cool (preferably in a cooler) and out of sunlight while in the field.

After returning from the field, place samples in a cool area (refrigerator about 0 to 4 ^o^C). Do not freeze unless specified for some specific biological analyses.

Double bag each sample and follow all applicable APHIS and local regulations for shipping and transportation of soil sample.

To process, air-dry in a cool location. See “Preparation of sample” section for guidance on preparing corresponding subsample for related analysis. Follow the established protocol for laboratory analysis.

**B. Preparation of soil sample**:

**Safety**

Use a face-shield, goggles and mask or other forms of respirators to prevent or reduce inhalation of dust. If using laboratory jaw crusher and pulverizer, use ear plugs, and keep clothes and hands away from the machine while it’s in use.

**Equipment**

- Electric Balance, ±1-g sensitivity and 15-kg capacity
- Cardboard trays for sample storage
- Trays, plastic
- Sieves, squares-hole, stainless steel, full-height
  - 180 µm, 80 mesh.
  - 2 mm, 10 mesh.
  - 4.75 mm, 4 mesh.
  - 8 mm, ⁵⁄₁₆ in
  - 19 mm, ¾ in
  - 76 mm, 3 in
- 200-mesh, 75 µm, nylon cloth sieve
- 40-mesh, 0.425 mm
- Pulverizer
- Wooden rolling pin
- Rubber roller
- Laboratory jaw crusher, Model BB200, Retsch Inc., Newton, PA
- Metal plate, 76 x 76 x 0.5 cm
- Containers, paper, 12-oz, with lids
- Containers, plastic, 1-pint, 1, 4, and/or 8 oz with tops
- Scintillation glass vials, 20-mL
- Metal weighing cans, 2-oz
- Brown Kraft paper
- Air compressor, Cast-iron Series, SpeedAire, Campbell Hausfeld Mfg. Co., Harrison, OH
- Planetary ball mill, Fritsch, Model P-5, Lab Synergy, VWR, Radnor, PA
- Rotor mill, Fritsch, Model P-14, VWR, Radnor, PA
- Stein Mill, Steinlite Corp., Atchison KS
- Syalon balls, 12- to 15-mm, and bowls, 80-mL
- Metal weighing cans, 2 oz
- Cross beater mill, Fritsch, Model P-16, VWR, Radnor, PA
- Downdraft tables, 36 in x 72 in, Dynamo Downdraft Cartridge Table, Air Cleaning Specialists, Inc., Fenton, MO

**Reagent**

- 1 N HCl

**Procedures**

Preparation of bulk samples

- Weigh (g) soil sample in bag as field-moist weight and record weight.
- Field-moist whole-soil samples:
  - For laboratory analyses that require field-moist whole-soil samples, remove rock fragments, take representative subsamples and store in the refrigerator for future analysis.
  - For laboratory analyses that require sieved (<2mm fraction) field-moist samples, remove rock fragments, sieve by forcing the samples through a 2-mm screen standard sieve using a rubber stopper or by hand. Proceed to use the homogenized sieved subsamples for analysis (like field-moist/oven-dry ratio or moisture content) or store in the refrigerator for further analysis.
- Air-dry samples:
  - Weigh (g) the samples before air-drying and record the weight. Spread the soil samples thinly on a tray after thoroughly mixing the samples. Air-dry in oven at 30 to 35 ^o^C for 3 to 7 days. The drying time may be shortened or extended based on the moisture content. Weigh (g) the air-dried sample and record the weight (air-dry/oven-dry ratio is required for air-dry analysis)
  - If whole-soil is required for lab analysis, then select representative subsample from air-dried sample and crush to <8mm fraction for KSSL aggregate stability (check KSSL_WSA method code in method index above or *cooplabmethod* table in the DSP4SH database).
  - For laboratory analyses that require <2mm fraction, crush the soil samples by hand or using lab crusher. Force the processed soil samples through a 2-mm sieves and weigh accordingly before proceeding on further analysis. Most DSP4SH tests require <2 mm fraction.
  - For laboratory analyses that require fine-earth (≈180µm) fraction, select representative subsamples from air-dried, sieved <2mm fraction and processed in a cross beater mill to ≈180µm fraction using ≈80 mesh in a 20 mL scintillation glass vial. This is important for total C and N analyses measured by combustion (check TC_pct, Effervescence, SOC_est, SOC_pct, SOC_loi, SOC_pct, and TN_pct in method index table or *cooplabmethod* table in the DSP4SH database).

To check for the presence of carbonates, place 1g of the air-dry fine-earth fraction in porcelain plate, add reverse osmosis water, and stir to remove entrapped air. Add 1 *N* HCl to the soil, observe amount of effervescence, and record as follows:

*None* – No visual effervescence.

*Very Slightly* – Bubbles rise at a few points in the sample and tiny streams of bubbles or very slower stream of larger bubbles.

*Slight* – More small and larger bubbles than very slight reaction.

*Strong* – More large bubbles than the slight reaction.

*Violent* – Entire sample depicts violent reaction and release of many large bubbles.

- Coarse fragment:

Sieve a representative horizon sample (≈30kg) to measure rock fragment that have a maximum diameter of 75mm using a 76-mm sieve. Discard the >75-mm sample. Weigh and record weight (kg) of <75-mm fraction. Sieve the <75mm fraction with a 2-mm sieve. Weigh and record weight (kg) of 2- to 75-mm fraction. Convert kg to g for calculation.

**Calculations**

The calculation of coarse fragment is based on oven-dry weight (*odw*). Air-dry/oven-dry ratio can be used to determine the *odw* of <2mm fraction of sample. Then percentage 2- to 75mm fraction can be determined using the following calculations:

Percentage fraction of 2-75mm fraction = (A/B) x 100

Where:

A=Weight of 2- to 75-mm fraction (g)

B=Weight of <75mm fraction (g)

Determine the *odw* by weighing the sample (field-moist - FM or air-dry - AD) after oven-drying at 110 ^o^C for 24hr or by using the calculation as follows:

oven-dry weight (*odw*) = (C/D)

Where:

C = FM weight (g) or AD weight (g)

D = field-moist weight/oven-dry weight ratio **or** air-dry weight/oven-dry weight ratio

**Report**

Weight (kg) of field-moist, <75-mm fraction

Weight (g) of field-moist soil sample

Weight (g) of air-dry soil sample

Weight (g) of air-dry processed soil sample

Weight (g) of 2- to 5-mm fraction

Effervescence with HCl (none, very slight, slight, strong, violent)

**References**

Brown, L. E., & Reinsch, T. G. (1993). Collection and Preparation of Soil Samples for the Federal Soil Survey Laboratory Program. https://doi.org/10.1520/STP23869S

Pribyl, D. W. (2010). A critical review of the conventional SOC to SOM conversion factor. Geoderma, 156(3), 75–83. https://doi.org/10.1016/j.geoderma.2010.02.003

# **Laboratory Method 1: Soil Organic Carbon (SOC)**

Adapted from USDA NRCS. (2023). [Kellogg Soil Survey Laboratory Methods Manual, Soil Survey Investigations Report No. 42, Version 6.0, Part 1: Current Methods (usda.gov)](https://www.nrcs.usda.gov/sites/default/files/2023-01/SSIR42.pdf)

Method code: SOC_pct

**Overview**

The difference between the total carbon (C) and the inorganic C is an estimate of the organic C in a soil. The inorganic C should be approximately equivalent to the carbonate value measured by carbon dioxide (CO2) evolution under strong acid (Nelson and Sommers, 1996). Total carbon in a soil is determined by dry combustion, and the amount of carbonate is determined by treating a sample with hydrochloric acid (HCl) and then taking a manometric measurement of the evolved CO2. The amount of carbonate is then calculated as a calcium carbonate (CaCO3) equivalent. Organic C defines mineral and organic soils. In soil taxonomy, organic C is used at the lower taxonomic levels, such as the ustollic and fluventic subgroups (Soil Survey Staff, 2014).

**Calculation**

SOC (%) = Total C (%) – Inorganic C (%)

where:

SOC (%) = Estimated soil organic carbon

Total C (%) = Value determined by Laboratory Method 1a

Inorganic C (%) = Value determined by Laboratory Method 1b

**Collecting, Handling, and Processing of Soil Samples**

Ensure the soil sample collection bags are properly labeled. Keep collected samples cool and out of sunlight while in the field (preferably in a cooler).

After returning from the field, the field sample is air-dried at 30 to 35 ^o^C, crushed and sieved to <2mm and then milled to <180µm using 80-mesh.

### **Laboratory Method 1a: Total Carbon, Dry Combustion Method**

**Application**

Soil organic matter (SOM) has been defined as the organic fraction of the soil excluding undecayed plant and animal residue, and it has been used synonymously with “humus” (Soil Science Society of America, 1997). For laboratory analyses, however, the soil organic matter generally includes only the organic material that passes through a 2-mm sieve with soil particles (Nelson and Sommers, 1982). The organic matter content influences many soil properties, including capacity of water retention; extractable bases; capacity to supply nitrogen (N), phosphorus (P), and micronutrients; stability of soil aggregates; and soil aeration (Nelson and Sommers, 1996). Organic C consists of the cells of microorganisms; plant and animal residue at various stages of decomposition; stable “humus” synthesized from residue; and nearly inert and highly carbonized compounds, such as charcoal, graphite, and coal (Nelson and Sommers, 1982). Because organic C is a major component of soil organic matter, a measurement of organic C can be an indirect determination of organic matter.

The Kellogg Soil Survey Laboratory (KSSL) currently uses a direct determination of soil organic matter. The organic matter is destroyed, and then the loss in the weight of the soil is taken as a measurement of the organic matter content. The percent organic matter lost on ignition (400 °C) can be used instead of the estimates of organic matter content from the now obsolete wet combustion method.

Total carbon—Total C is the sum of organic and inorganic C. Most of the organic C is associated with the organic matter fraction, and the inorganic C generally is in the carbonate minerals. The organic C in mineral soils generally ranges from 0 to 12 percent (Nelson and Sommers, 1996). Total C is quantified by two basic methods, i.e., wet or dry combustion. The KSSL uses dry combustion. In total C determinations, both forms of C in a soil are converted to CO2 and then the evolved CO2 is quantified.

**Summary of Method**

An air-dry (80 mesh, <180 μm) sample is packed in tin foil, weighed, and analyzed for total C, N, and S (sulfur) using an elemental analyzer. The elemental analyzer works according to the principle of catalytic tube combustion in an oxygenated CO2 atmosphere at a high temperature. The combustion gases are freed from foreign gases. Each of the desired measuring components (dinitrogen [N2], CO2, and sulfur dioxide [SO2]) are separated out by specific adsorption columns and are determined in succession with a thermal conductivity detector. Helium is the flushing and carrier gas. Percent total C, N, and S are reported by methods, respectively.

**Interferences**

Avoid contamination by body grease or perspiration during sample packing. Avoid substance loss after weighing by exact folding of the tin foil around the sample. Minimize air in the sample material (falsifying the N value) by compressing the sample packing. Insufficient O2 dosing reduces the catalysts, decreasing their effectiveness and durability. Burnt sample substance that remains in ash finger falsifies the results of subsequent samples. Tungsten trioxide (WO3) is used as a sample additive and combustion filler to aid combustion or bind interfering substances (alkaline or earth-alkaline elements, nonvolatile sulfates).

**Safety**

Exhaust gas pipes should lead into a hood to ventilate fumes. Do not analyze aggressive combustible products. Before working on electrical connections (adsorption columns) or changing reaction tubes, cool down the instrument and shut it off. Wear gloves and safety glasses at all times during operation and maintenance of instrument.

**Equipment**

Elemental analyzer with online electronic balance (0.1 ±mg sensitivity) and an AC or elemental analyzer equipped with an automatic sample feeder and an online electronic balance (±0.1 mg sensitivity). Parts included depend on the make and model of the analyzer.

Many instruments include—

- Combustion tube
- Reduction tube
- Gas purifier (u-tube)
- O_2_ lance
- Tin foil cups and/or ceramic boats
- Computer with software for the analyzer and a printer

**Reagents**

Reagents needed vary with the make and model of the analyzer. Use the reagents specified for total C analysis by the manufacturer of the analyzer.

Many instruments require—

- Sulfanilic acid, calibration standard, 41.6 percent C, 4.1 percent hydrogen (H), 8.1 percent nitrogen (N), 27.7 percent oxygen (O), and 18.5 percent sulfur (S)
- Tungsten oxide powder, sample additive
- Tungsten trioxide granulate, combustion tube filling
- Quartz wool
- Silver wool
- Helium, carrier gas, 99.996 percent purity
- Oxygen, combustion gas, 99.995 percent purity

**Elemental Analyzer Calibration and Analysis**

- Refer to the manufacturer’s manual for operation and maintenance of the elemental analyzer. Conditioning of the elemental analyzer and determination of factor and blank value limit are included in the daily measuring routine. The analyzer furnace temperature must be 1350 °C to achieve full combustion (e.g., Leco Corporation 2014). Soil samples for analysis should be finely ground. The sample size ranges from 0.200 to 0.05 g (oven-dried weight), depending on the instrument.
- Perform a calibration that covers the desired working range of sample values. The calibration test analyzes sulfanilic acid with carbon content at different ratios. The computer program automatically computes the calibration function (linear, polynomial, or mixed). The calibration typically remains stable for at least 6 months. Recalibration is recommended when the daily factor is outside the range of 0.9 to 1.1 or if components that influence the results (e.g., detector or adsorption column) are exchanged. Changing the desorption temperature of adsorption columns may also make recalibration necessary.
- Add 0.100 g of tungsten oxide to tin foil and tare. Pack a homogenized, finely ground, air-dried, and weighed (0.100 to 0.05 g) soil sample in the tin foil, and place it into the carousel of the automatic sample feeder for the elemental analyzer. The sample weight is based on visual observation of the sample and related to element content, homogeneity, and combustion behavior of the sample. The sample weight is entered into the computer from an online electronic balance via an interface. Quality control (QC) is performed at a minimum on every 35 to 40 samples.

**Calculations**

C (%) = Ci x AD/OD

where:

C (%) = C (%), oven-dry basis

Ci = C (%) instrument

AD/OD = Air-dry/oven-dry ratio (If unavailable, use 1.0.)

**Report**

Report total C to the nearest 0.01 percent.

### **Laboratory Method 1b: Soil Inorganic Carbon, Calcium Carbonate**

**Application**

The distribution and amount of calcium carbonate (CaCO_3_) are important factors affecting the fertility, erosion, available water capacity, and genesis of a soil. Calcium carbonate provides a reactive surface for adsorption and precipitation (e.g., phosphate, trace elements, and organic acids) (Loeppert and Suarez, 1996). The determination of CaCO_3_ equivalent is a criterion in soil taxonomy (Soil Survey Staff, 2014). The carbonate content of a soil is used to define carbonatic, particle-size, and calcareous soil classes and to define calcic and petrocalcic horizons (Soil Survey Staff, 2014). The formation of calcic and petrocalcic horizons has been correlated to a variety of processes, including translocation and net accumulation of pedogenic carbonates from a variety of sources and alteration of lithogenic (inherited) carbonate to pedogenic carbonate (soil-formed carbonate through in situ dissolution and re-precipitation of carbonates) (Rabenhorst et al., 1991). The CaCO_3_ equivalent commonly is reported on the <2-mm soil fraction; however, the equivalent is determined on both the <2-mm and 2- to 20-mm fractions in some soils that have hard carbonate concretions. The CaCO_3_ equivalent is routinely determined by the KSSL if the calcium dichloride (CaCl_2_) pH is >6.95 and/or effervescence after treatment with 1 N HCl is violent, strong, slight, or very slight.

**Summary of Method**

The soil samples are treated with HCl, the evolved CO_2_ is measured monometrically, and then the amount of carbonate is calculated as percent CaCO_3_.

**Interferences**

A chemical interference is the reaction by the acid with other carbonates (e.g., carbonates of magnesium [Mg], sodium [Na], and potassium [K]) that may be in the soil sample. The calculated CaCO_3_ is only a semiquantitative measurement (Nelson, 1982). Temperature changes in the reaction vessel may cause analytical interference. When sealing the vessel, do not hold the vessel any longer than necessary to tighten the cap. The internal pressure must be equalized with the atmosphere. After the septum is pierced with a needle, ≈5 to 10 seconds are required to equalize the internal pressure of the bottle. With extensive use, septa leak gas under pressure; therefore, they should be replaced at regular intervals. Do not touch the glass of the vessel when reading the pressure.

**Safety**

- Wear protective clothing (coats, aprons, sleeve guards, and gloves) and eye protection (face shields, goggles, or safety glasses) when handling acids.
- Thoroughly wash hands after handling acids. Use a fume hood when diluting concentrated HCl. Use the safety showers and eyewash stations to dilute spilled acids. Use sodium bicarbonate and water to neutralize and dilute spilled acids.
- The gelatin capsule may leak acid while being filled. Keep other personnel away from the area when filling capsules.
- High pressure may develop inside the bottle if there is a large amount of a calcareous sample.
- Do not use more than 2 g of a sample in a bottle. If high pressure develops in the bottle, release the pressure by venting the gas with a syringe needle.
- Some bottles may break without shattering. Discard bottles that have hairline cracks or other obvious defects.

**Collecting, Handling, and Processing of Soil Samples**

Ensure the soil sample collection bags are properly labeled. Keep collected samples cool and out of sunlight while in the field (preferably in a cooler).

After returning from the field, place samples in a cool area (refrigerator).

Process samples by air-drying in a cool location and sieving through a 2-mm screen.

Follow the established protocol for laboratory analysis.

**Equipment**

- Electronic balance, ±0.10-mg sensitivity.
- Electronic balance, ±1-mg sensitivity.
- Threaded weighing bottles, wide mouth, clear glass, standard, 120 mL (4 fl. oz.), 48-mm neck size. For best results, grind rim of bottle with 400- to 600-grit sandpaper on a flat glass plate.
- Machined PVC caps for threaded 120 mL (4 fl. oz.) weighing bottles, 54-mm diameter with 12.7-mm diameter hole drilled in center, O-ring seal.
- O-rings, 3.2 x 50.8 x 57.2 mm (⅛ x 2 x 2¼ in).
- Flanged stopper no. 03-255-5, Fisher Scientific. Place in machined cap.
- Manometer, hand-held gauge and differential pressure, PCL-200 Series, Omega Engineering, Stanford, CT.
- Hypodermic needle, 25.4 mm (1 in), 23 gauge. Connect needle to pressure tubing on transducer.
- Mechanical rotating shaker, 140 rpm, Eberbach 6140, Eberbach Corp., Ann Arbor, MI.

**Reagents**

- Reverse osmosis deionized (RODI) water, ASTM Type I grade reagent water.
- Methyl red indicator.
- Hydrochloric acid (HCl), concentrated, 12 N.
- HCl, 3 N. Dilute 500 mL of concentrated HCl with 1500 mL of RODI water. Add a few crystals of methyl red indicator. Methyl red indicator turns yellow if HCl is consumed by sample. If this reaction occurs, adjust the sample size (smaller).
- Gelatin capsule, 10 mL, size 11, Torpac Inc., Fairfield, NJ.
- Glycerin, USP. Put the glycerin in a small squeeze bottle, and use it to lubricate the O-rings.
- CaCO_3_, Ultrex, assay dried basis 100.01 percent.

**Procedure**

Manometer calibration

Calibrate the manometer quarterly or whenever equipment changes (e.g., old rubber septum replaced). Calibrate by weighing three replicates of CaCO_3_ standards (0, 0.025, 0.05, 0.1, 0.2, 0.3, 0.4, 0.5, 0.75 g). Weigh to the nearest 0.1 mg. Dry the standard samples in the oven for 2 hours at 110 °C. Remove samples from oven, place in desiccator, and cool to ambient temperature.

<2-mm basis

- A CaCl_2_ pH >6.95 generally is used as an indicator of the presence of carbonates. The presence of carbonates (effervescence with HCl) also is checked during laboratory preparation.
- Weigh 0.5 to 2 g of fine-grind, air-dry soil sample to the nearest mg and place in a 120-mL, wide-mouth bottle. Run three blanks and a quality control check sample for every batch of 24 samples. The quality control check sample serves as a single point check. Vary the sample weight according to the CaCO_3_ content in sample based on effervescence as follows:
  - Use a 2-g sample weight if effervescence is none, very slight, or slight.
  - Use a 1-g sample weight if effervescence is strong.
  - Use a 0.5-g sample weight if effervescence is violent.
- Lubricate the O-ring in bottle cap with glycerin from a squeeze bottle.
- Dispense 10 mL of 3 N HCl into a gelatin capsule, and carefully place the top on the capsule. If the HCl squirts or leaks out of the capsule, discard the capsule.
- Place the capsule in a bottle. Cap the bottle immediately.
- Release pressure in the bottle by piercing the stopper with a hypodermic needle. Remove the needle after ≈5 to 10 seconds.
- After 5 to 10 minutes, the HCl will dissolve through the capsule. Shake the bottle on a shaker at a rate of 140 rpm for the first 10 minutes and the last 10 minutes of a 1-hour interval at room temperature (20 ±2 °C). After the 1-hour interval, measure the pressure in the bottle by piercing the stopper of the cap with a hypodermic needle connected to the manometer.
- Autozero the manometer before taking readings. Record the manometer readings (mm mercury [Hg]).

<20-mm basis (only for soil samples that have hard carbonate concretions)

- Determine the carbonate content of the 2- to 20-mm fraction on a fine-grind (<180 μm), air-dry sample by the <2-mm-basis method.
- The carbonate in the 2- to 20-mm fraction and in the <2-mm fraction are combined and converted to a <20-mm soil basis.

**Calculations**

- Correct the manometer readings as follows:

CR = (MR − BR)

where:

CR = Corrected reading

MR = Manometer reading

BR = Blank reading

Three blanks are run with each batch of 24 samples. The average of the three blanks is used as the BR.

- Calculate the regression equation for the corrected manometer readings. Use the CaCO_3_ weights as the dependent variable (regressed or predicted values) and the corresponding manometer readings as the independent variable.
- Use the corrected (CR) linear regression (slope, intercept) equation to estimate percent CaCO_3_ in the sample as follows:

CCE = [(CR x Slope + Intercept)/sample weight (g)] x AD/OD

where:

CCE = Calcium carbonate equivalent (%) in <2-mm fraction or 2- to 20-mm fraction

CR = Corrected manometer reading

AD/OD = Air-dry/oven-dry ratio (If not available, use 1.0.)

Carbonate = (A x B) + [C x (1-B)]

where:

Carbonate = Carbonate as CaCO_3_ on a <20-mm basis (percent)

A = CaCO_3_ in <2-mm fraction (percent)

B = Weight of <20-mm fraction minus weight of 2- to 20-mm fraction divided by weight of <20-mm fraction

C = CaCO_3_ in 2- to 20-mm fraction (percent)

**Report**

Report CaCO_3_ equivalent as a percentage of oven-dry soil to the nearest whole number.

### **Laboratory Method 1c: Soil Organic Carbon, Loss on Ignition**

Method code: SOC_loi

**Application**

The mineral content consists of the plant ash and soil particles that remain after removal of organic matter. The percentage of organic matter lost on ignition can be used to define organic soils in place of estimates of organic matter by the Walkley-Black organic C method (method obsolete). The determination of organic matter by loss on ignition is a taxonomic criterion for organic soil materials (Soil Survey Staff, 2014). Organic C data by Walkley-Black are generally considered invalid if organic C >8 %.

**Summary of Method**

Dry sample overnight at 110 °C in moisture can. Cool and weigh. Place sample in a cold muffle furnace and raise the temperature to 400 °C. Heat sample overnight (16 h), cool, and weigh. The ratio of the weights (400 °C/110 °C) is the mineral content percentage.

**Interferences**

The sample must be placed in a cold muffle furnace to prevent rapid combustion and sample splattering.

**Safety**

Use caution when the muffle furnace is hot. Wear protective clothing and goggles. Handle the heated material with tongs.

**Equipment**

- Metal weighing tins
- Oven, 110 °C
- Muffle furnace, 400 °C
- Electronic Balance, ±0.01-g sensitivity

**Procedure**

- Place a 10 to 15 g sample in a tared weighing tin
- Dry sample at 110 °C overnight
- Remove sample from oven, cap, and cool in a desiccator
- When cool, record weight to nearest 0.01 g
- Place sample and weighing tin in a cold muffle furnace. Raise temperature to 400 °C. Heat overnight (16 h)
- Remove sample from oven, cap, and cool in a desiccator
- When cool, record sample weight to nearest 0.01 g

**Calculations**

Mineral Content (%) = (RW / ODW) x 100

where:

RW = Residue weight after ignition

ODW = Oven-dry soil weight

Organic matter percent can then be calculated as follows:

Organic Content (%) = 100 − Mineral Content (%)

**Laboratory Method 1d: Soil Organic Carbon, estimated by Walkley-Black Method**

Method code: SOC_est

**Application**

Organic C by the Walkley-Black method is a wet combustion technique to estimate organic C. A correction factor is used to convert the Walkley-Black value to an organic matter content. A common value for the factor is 1.724 based upon the assumption that soil organic matter contains 58% organic C. A review of the literature reveals that the factor is highly variable, not only among soils but also between horizons in the same soil (Broadbent, 1953; Pribyl, 2010). In addition, a recovery factor is used because the Walkley-Black method does not completely oxidize all the organic C.

**Summary of Method**

The SSL uses the Walkley-Black modified acid-dichromate FeSO4 titration organic carbon procedure. A sample is oxidized with 1 *N* potassium dichromate and concentrated sulfuric acid (1:2 volume ratio). After 30 min, the reaction is halted by dilution with water. The excess dichromate is potentiometrically back titrated with ferrous sulfate. A blank is carried throughout the procedure to standardize the ferrous sulfate. Percent organic C is reported on an oven-dry soil basis.

**Interferences**

Dichromate methods that do not use additional heating do not give complete oxidation of organic matter. Even with heating, the recovery may not be complete. Walkley and Black (1934) determined an average recovery factor of 76%. Other studies have found recovery factors ranging from 60% to 86%. Thus, an average correction factor yields erroneous values for many soils. The Walkley-Black method is only an approximate or semiquantitative estimate of organic C.

Maintain the ratio of dichromate solution to concentrated H2SO4 at 1:2 to help maintain uniform heating of the mixture.

The presence of significant amounts of chloride in the soil results in a positive

error. If the chloride in the soil is known, use the following correction factor (Walkley, 1947) for the organic C.

Organic C (%) = Apparent soil C % − (Soil Cl^−^ %) / 12

The presence of significant amounts of ferrous ions results in a positive error (Walkley, 1947). The dichromate oxidizes ferrous to ferric iron.

Cr_2_O_7_ ^2−^ + 6 Fe^2+^ + 14 H^+^ = 2 Cr^3+^ + 6 Fe^3+^ + 7 H_2_O

The presence of manganese dioxide results in a negative error (Walkley,

1947). When heated in an acidic medium, the higher oxides of manganese, e.g.,

MnO2, compete with dichromate for oxidizable substances.

2 MnO_2_ + C^o^ + 4 H+ = CO_2_ + 2 + 2 Mn + 2 H2O

All dichromate methods assume that the organic C in the soil has an average oxidation state of zero and an equivalent weight of 3 g per equivalent when reacting with dichromate. When the soil has carbonized material, e.g., charcoal, graphite, coal and soot, the Walkley-Black method gives low recovery of this material, i.e., recovery range is from 2 to 36%.

**Safety**

Wear protective clothing (coats, aprons, sleeve guards, and gloves) and eye protection (face shields, goggles, or safety glasses) when preparing acids and dichromate. Toxic chromyl chloride may be released from the sample if high concentrations of chloride are present. Use the fume hood to contain the gases released by this procedure. Use the safety showers and eyewash stations to dilute spilled acids. Use sodium bicarbonate and water to neutralize and dilute spilled acids and dichromate. Follow the manufacturer’s safety precautions when using the automatic titrator.

**Equipment**

- Electronic balance, ±1-mg sensitivity
- Titration beakers, borosilicate glass, 250 mL
- Automatic dispenser, 5 to 20 mL, Oxford no. 470 or equivalent, for K2Cr2O7,

capable of volume adjustment to 10.00 ±0.01 mL, 0.5% reproducibility

- Dispenser, Zippette 30 mL or equivalent, for concentrated H_2_SO_4_, Brinkmann Instruments, Inc
- Shaker, Eberbach 6000 power unit, fitted with spring holders for titration beakers, reciprocating speed of 60 to 260 epm, with 6040 utility box carrier and 6110 floor stand, Eberbach Corp., Ann Arbor, MI
- Automatic titrator, Metrohm 686 Titroprocessor Series 04, 664 Control Unit, 674 Sample Changer Series 5, and 665 Dosimat Series 14, Metrohm Ltd., Brinkmann Instruments, Inc
- Platinum electrode, Metrohm part no. 6.0412.000

**Reagents**

- Distilled deionized (DDI) water
- Potassium dichromate, 1.000 *N*, primary standard. Dissolve 49.035 g of K_2_Cr_2_O_7_ reagent, dried at 105 ^o^C, in 1-L volumetric flask with DDI water
- Sulfuric acid (H_2_SO_4_), concentrated, reagent
- Ferrous sulfate, 1 *N*, acidic. Dissolve 1 kg of FeSO_4_•7H_2_O in 6 L of DDI water. Carefully add 640 mL of concentrated H_2_SO_4_ with stirring. Cool and dilute to 8 L with DDI water.

**Procedure**

*Digestion of Organic C*

- Weigh 1.000 g air-dry soil and place in a titration beaker. If the sample contains >3% of organic C, use a smaller sample size. Refer to Table 1 for sample weight guide. If sample size is <0.5 g, use <80-mesh soil. If sample size is >0.5 g, use <2-mm soil
- With automatic dispenser, add 10.00 mL of K_2_Cr_2_O_7_ solution to the titration beaker. Mix by swirling the sample
- Use the dispenser to carefully add 20 mL of concentrated H_2_SO_4_ to the beaker. Mix by swirling solution. Adjustment in the amount of K_2_Cr_2_O_7_ added to sample requires appropriate adjustment in the amount of H_2_SO_4_ so that a 1:2 volume is maintained
- Place titration beaker on the reciprocating shaker and shake 1 min. If the dichromate-acid mixture turns a blue-green color, all the dichromate has been reduced. Add more dichromate and acid to maintain a 1:2 volume
- Refer to Table below for dichromate:acid volumes

*Digestion of organic C. Guide for sample weight and dichromate:acid volumes*:

| OC (%) | Sample (g) | K_2_Cr_2_O_7_ (mL) | H_2_SO_4_ (mL) |
| --- | --- | --- | --- |
| 0-3 | 1.000 | 10.00 | 20 |
| 3-6 | 0.500 | 10.00 | 20 |
| 3-6 | 1.000 | 20.00 | 40 |
| 6-12 | 0..500 | 20.00 | 40 |
| 12-24 | 0.250 | 20.00 | 40 |
| 24-50 | 0.100 | 30.00 | 60 |

- Place the beaker on a heat resistant surface for 30 min
- Add ≈180 mL DDI water to the beaker to stop the reaction

*Titration of Excess Dichromate*

- Titrate eight reagent blanks at the start of each batch to determine the normality of the ferrous sulfate. A blank is 10.00 mL K_2_Cr_2_O_7_ plus H_2_SO_4_ without soil. The average titer is used for the blank titer value
- Place the appropriate blanks and samples in the sample holder magazines and place on the sample changer
- Refer to the manufacturer’s instruction manual for operation of automatic titrator
- Set the endpoint to 700 mV. Set the controls of the 664 Control Unit to the appropriate settings
- Prime the burette with 50 mL of ferrous sulfate solution before starting the titrations
- When a long series of samples are being titrated, intersperse blank samples throughout the titrations. The blank titer drifts over time, mainly because of the temperature change of the solution. Any sample with a titer of less one milliliter and/or endpoint of less than 620 millivolts should be reanalyzed
- Press “Start” on the titrator

**Calculations**

OC (%) = [(Blank x Volume) − (10 x Titer) x 3 x 100 x AD/OD] / [Blank x Sample

Weight (g) x 0.77 x 1000]

where:

OC (%) = Organic C (%)

Blank = Average titer of reagent blanks (mL)

Volume = Volume of 1 *N* K_2_Cr_2_O_4_ (mL)

Titer = Titer of FeSO_4_ (mL)

AD/OD = Air-dry/oven-dry ratio (method 4B5)

3 = Equivalents per C (assumed)

1000 = Meq eq^−1^

100 = Convert to 100-g basis

0.77 = Assumed C oxidation factor

**References**

Nelson, D.W., and L.E. Sommers. 1982. Total carbon, organic carbon, and organic matter. p. 539–579. In A.L. Page, R.H. Miller, and D.R. Keeney (eds.) Methods of soil analysis. Part 2. Chemical and microbiological properties. 2nd ed. Agron. Monogr. 9. ASA and SSSA, Madison, WI.

Nelson, D.W., and L.E. Sommers. 1996. Total carbon, organic carbon, and organic matter. p. 961–1010. In D.L. Sparks (ed.) Methods of soil analysis. Part 3. Chemical methods. No. 5. ASA and SSSA, Madison, WI.

Soil Science Society of America. 1997. Glossary of soil science terms. Rev. ed. Soil Sci. Soc. Am., Madison, WI.

Loeppert, R.H., and D.L. Suarez. 1996. Carbonate and gypsum. p. 437–474. In D.L. Sparks (ed.) Methods of soil analysis. Part 3–Chemical methods. Soil Sci. Am. Book Series No. 5. ASA and SSSA, Madison, WI.

Soil Survey Staff. 2014. Keys to soil taxonomy. 12th ed. USDA–NRCS.

Rabenhorst, M.C., L.T. West, and L.P. Wilding. 1991. Genesis of calcic and petrocalcic horizons in soils over carbonate rocks. p. 61–74. In W.D. Nettleton (ed.) Occurrence, characteristics, and genesis of carbonate, gypsum, and silica accumulations in soils. Soil Sci. Soc. Am. Spec. Publ. No. 26. ASA and SSSA, Madison, WI

Nelson, R.E. 1982. Carbonate and gypsum. p. 181–197. In A.L. Page, R.H. Miller, and D.R. Keeney (eds.) Methods of soil analysis. Part 2. Chemical and microbiological properties. 2nd ed. Agron. Monogr. 9. ASA and SSSA, Madison, WI.

Broadbent, F.E. 1953. The soil organic fraction. Adv. Agron. 5:153–183.

Pribyl, D. W. (2010). A critical review of the conventional SOC to SOM conversion factor. Geoderma, 156(3), 75–83. https://doi.org/10.1016/j.geoderma.2010.02.003

# **Laboratory Method 2: Yoder Wet Sieving Method**

Adapted from Mikha, M. M., & Rice, C. W. (2004). Tillage and Manure Effects on Soil and Aggregate-Associated Carbon and Nitrogen. *Soil Science Society of America Journal*, 68(3), 809–816. https://doi.org/10.2136/sssaj2004.8090

Method code: Yoder_AggStab_MWD, Yoder_AggStab_TotalWS, Yoder_WS_2-8, Yoder_WS_pt5-2, Yoder_WS_250-500, Yoder_WS_53-250

**Application**

An aggregate is a group of primary soil particles that cohere to each other more strongly than to other surrounding soil particles (Soil Science Society of America, 1997). Disaggregation of soil mass into aggregates requires the application of a disrupting force. Aggregate stability is a function of the capacity of cohesive forces among particles to withstand the applied disruptive force. The analysis of soil aggregation can be used to evaluate or predict the effects of various agricultural techniques, such as tillage, additions of organic matter, and erosion by wind and water (Nimmo and Perkins, 2002). The measurement can serve as a predictor of the potential for infiltration and soil erosion.

**Summary of Method**

This method provides measurements of multiple stable aggregate fractions following a 10-minute agitation at 30 cycles per minute with a Yoder-style machine. Initially, air-dry samples are pre-wetted for 10 minutes before sieving to overcome differential in speed of wetting related to the varying content of clay in samples.

The SOP presented here is from Mikha & Rice (2004). The number of sieves used were reduced so that there are only three fractions: macroaggregates (> 250 μm), microaggregates (250-53 μm), and non-aggregated material (< 53 μm). However, sieve sizes can be substituted or more sieves can be added if appropriate for specific samples. Testing should be performed before choosing sieve sizes.

Weigh, record and calculate mean weight diameter

Dry @ 70 ^o^C overnight or until devoid of moisture

Oscillate for 10mins @30cycle/min (stroke length 4cm) with 10 mins pre-wet time

50 ± 0.05 g (air-dried soil)

Flowchart summarizing Yoder wet sieving method

**Interferences**

Air bubbles in the sieve can create tension in the water and reduce the percentage of aggregates that are retained on the sieve. Variation in the moisture content of air-dry soils can affect results.

**Safety**

If ovens are used, hot surfaces are a hazard. Because moving parts of the Yoder machine are a hazard, keep hands off the machine when it is on. Inspect and maintain electrical components, and only use outlets that have a ground fault circuit interrupter (GFCI). Follow standard laboratory safety precautions.

**Collecting, Handling, and Shipping of Soil Samples**

Ensure the soil sample collection bags are properly labeled. Keep collected samples cool and out of sunlight while in the field (preferably in a cooler). Do not dry or freeze.

After returning from the field, place samples in a cool area (refrigerator).

If shipping samples, do so in field-moist, undisturbed state, or process samples to <8 mm in the field and ship samples in moist or air-dried state. Pack samples so that they are not crushed or compacted during shipment. Double bag each sample and follow all applicable APHIS regulations. Ship using a 2- or 3-day service.

Follow “Preparation of Samples” section for guidance on processing sample for analysis.

**Equipment**

*Reusable supplies:*

- Bowls, Rubbermaid or equivalent, 1800 mL
- Sieves, square hole, stainless steel, 152-mm/6-in diameter (diameter is important), 50-mm (full) height.
- 2 mm; #10
- 500 μm; #35 (optional)
- 250 μm; #60
- 53 μm; #270
- Metal plates, 152-mm diameter or more
- Medium weigh boat
- Labeling tape
- Labeled aluminum pans (e.g., 1 lb. bread loaf pans) or 8 oz. glass jars, three per subsample (or one per number of sieves). Example of labeled jars for 2 replicates of sample 1:

**1A – 10, 1A – 60, 1A – 270**

**1B – 10, 1B – 60, 1B – 270**

- Rubber spatula
- Rinse bottle
- 5-gallon bucket or equivalent
- Labeled weigh boats (two per sample) A and B (Note: condiment cups are a useful alternative for weighing soils in advance and transporting them between spaces)
- Rubber policeman for aggregate breakdown

*Instrumentation*:

- Electronic balance, ±0.01-g sensitivity and 500-g capacity
- Forced-air drying Oven
- Yoder-style wet sieving apparatus (set for 30 cycles per min and 4 cm stroke length) and water tubes (4 per machine or whatever capacity available)
- Optional: Cart with wheels for sample storage during drying process
- Optional: Three-quarter sheet pans for transferring aggregate samples to and from drying oven
- Optional: 15-cm diameter funnel
- Optional: Bottle brush

**Reagent**

- Deionized (DI) water
- Dissolve 35.7 g of sodium hexametaphosphate solution (Na_4_P_2_O_7_) and 7.94 g of sodium carbonate (Na_2_CO_3_) in 1 liter of RO water.

**Preparation of Samples**

Gently break up moist, unprocessed samples and pass through an 8-mm sieve. Do not compact or crush samples. Air-dry all samples to a constant weight at 30 to 35 °C. Homogenize samples on a paper sheet by using corners of sheet to tumble samples repeatedly.

**Procedure**

**Labeling and Weighing Sample**

- Prepare samples: pre-label weigh boats or condiment cups with 2 replicates per sample (A and B). Tare each vessel and carefully weigh 50 ± 0.05 g (≈0.14g of air-dried soil cm^-2^ or 25g depending on the diameter of the sieve) of sample into each vessel.
- Prepare jars: pre-label aluminum pans or glass jars with labeling tape. Each individual sample/replicate will require a pan per sieve size. Therefore, if there are 3 sieve sizes, you will need 6 pans for 2 replicates. Record the mass of each pan in advance (M1).

Example: You have 20 soil samples to run that will be separated into 4 sieve sizes. You will need to pre-label 40 weigh boats/condiment cups and 160 pans/glass jars.

**Setup of Sieving Machine**

Machine Set-Up

- Check that machine runs for 30 cycles per minute and has a 4 cm stroke length.
- Check bracket and tube conditions, overall condition of equipment, and deal with any problems. Set the rotor on the device to its highest position.
- Stack the sieves in order (2 mm on top, 250 µm in middle, and 53 µm on bottom).
- Hang the sieves in the sieving bracket and make sure they are secure.
- Fill tube with DI water to fill line, and gently submerge the sieving bracket. Vigorously tap sieving stack against bottom of tube to release trapped air until no bubbles remain and then re-secure all sieving brackets. If necessary, connect all sieving brackets to the main frame that attaches to the motor, continuing to maintain that the rotor is set in its highest position.
- Adjust the water level in each tube so it is just above the screen (just enough to cover the soil) on the top sieve. Turn the motor on to test that no air is introduced underneath the 2 mm sieve at any point while the rotor is turning. Also, confirm that the water level in the tube outside the sieve is moving in concert with the water level inside the sieving bracket. If they are not moving in concert, you may still have trapped air in the sieving bracket. Any trapped or introduced air can alter the level of disruption experienced by aggregates.
- When levels are all confirmed, return the rotor to its highest position.

Day 1: Sieving

- Gently distribute the sample across the top sieve as evenly as possible. Start machine and timer (10 min). Allowing the soil to be submerged before the start of the wet-sieving process.
- Allow each sample to be sieved for 10 min. During this time, observe the samples and make note of anything of interest, such as complete slaking of sample, visible air bubbles, etc.
- When finished sieving, immediately remove the sieve sets from the tubes and position to drain excess water for a few minutes (engage draining position or set the sieves angled on the top of the tube).
- Using DI water from squirt bottles, gently rinse all aggregates from each sieve into its corresponding pre-weighed pan. Develop a setup that works for you: a funnel commensurate with the size of the sieve can help collect and direct aggregates into the pan or jar (but then be sure to rinse the funnel before removing). Be very careful to check all tools to make sure all aggregates have been added to the pan before moving on to the next fraction. NOTE: Water from the tube that has samples that have pass through all sieves including 53µm would be calculated by difference (adjusted soil weight minus sum of all other fractions).
- If the pan becomes too full to transport easily, use a second (pre-weighed) pan. Be sure to note on the pan that it is a second pan and for which sample ID#.
- Place filled pans in the soil drying oven at 70°C until all water has evaporated and the soil is dry (usually overnight).
- Rinse all sieves, brackets, and tubes and allow to dry. Do not dry sieves in oven.

Day 2: Record data and perform sand + gravel corrections

- Remove pans from oven when dry, allow to come to room temperature, and arrange in order to match your data sheet. Record the mass of each fraction (M_2_). This mass is considered the pre-corrected aggregate fractional mass.
- Next, corrections are performed on each fraction to ensure that the soil withheld by a sieve is in fact an aggregate and not just a large particle. Utilize sodium hexametaphosphate solution to disperse the dry aggregate sample by transferring the aggregate into the corresponding sieve. Place the sieve containing the dry aggregate in a bowl containing sodium hexametaphosphate solution. Triturate the aggregate with thumb and fore-finger or a rubber spatula on the side walls of the sieves to break up all aggregates (avoid crushing aggregates on the screen themself). Rinse the sand that is left on the sieve with water from the rinse bottle. Use cool or lukewarm water to avoid stretching of sieve mesh. Check the rinse water for clarity to confirm that all small particles have been rinsed through the sieve.
- As before, sand and gravel remaining on the screen are washed with a rinse bottle into the *same labeled pan* and placed back in the soil drying oven at 70°C until all water has evaporated and soil is dry (usually overnight).
- Cleanup: Rinse all sieves and rubber spatulas with water and allow to dry. Decant clear water off the 5-gallon bucket into the sink and compost any remaining soil slurry at the bottom of the bucket.

Day 3: Record data and cleanup

- As before, remove pans from oven when dry, allow to come to room temperature, and arrange in order to match your data sheet. Record the mass of sand and gravel for each fraction (M_3_). Remember that you will have already recorded the mass of the pan/jar for that sample.
- Cleanup: Remove labeling tape from all pans/jars and rinse with water. A sponge or bottle brush may be helpful to remove residue. Air-dry or dry in oven.

**Calculations**

- M_1_ = Mass pan/jar
- M_2_ = Pre-corrected aggregate fractional mass + mass pan/jar
- M_3_ = Mass sand and gravel (s/g) + mass pan/jar

Step 1: *Calculate corrected aggregate fractional mass*

You will calculate one value per fraction per replicate per sample.

$$Corrected aggregate fractional mass \left( g \right)=M_{2}-M_{3}$$

Step 2: *Calculate total aggregate mass per sample*

You will calculate one value per replicate per sample.

$$Total aggregate mass \left( g \right)= \sum_{i=fraction}^{n=\# of fractions} Corrected aggregate fractional {mass}_{i}$$

Step 3: *Calculate total sand and gravel per sample*

1. First, calculate the s/g fractional mass (one value per fraction per replicate per sample)

$$s/g fractional mass \left( g \right)= M_{3}- M_{1}$$

2. Next, calculate the total s/g per sample (one value per replicate per sample).

$$Total s/g mass \left( g \right)= \sum_{i=fraction}^{n=\# of fractions} s/g fractional {mass}_{i}$$

Step 4: *Calculate corrected sample mass*

You will calculate one value per replicate per sample.

$$Corrected sample mass (g)=Original sample mass-Total s/g mass$$

Step 5: *Calculate Sieve Mean Diameter (SMD)*

You will calculate one per sieve size.

$$Sieve mean diameter (mm)= \frac{opening in sieve passed+opening in sieve retained}{2}$$

Step 6: Calculate the water stable aggregates per sample in each size *fraction* (WS_frac_)*.*

You will calculate one value per fraction per replicate per sample.

$$fraction aggregates \left( no unit \right)= \frac{corrected aggregate fractional mass}{corrected sample mass}$$

Step 7: *Calculate fractional Mean Weight Diameter (MWD_frac_)*

You will calculate one value per fraction per replicate per sample.

$$MWD_{frac} (mm)=SMD\times fraction aggregates$$

Step 8: *Calculate the total MWD*

You will calculate one value per replicate per sample.

$$MWD \left( mm \right)= \sum_{i=fraction}^{n=\# of fractions} MWD_{frac(i)}$$

You will report your final value as MWD (mm).

**Quality Control**

Multiple replicates of each sample should be run, and the values averaged. If these differ from each other by more than a set threshold (suggest an average deviation of 5 percent from the common mean), then the sample should be rerun.

**Example** (using 50g weight of unknown sample (a rep) and 3 sieves):

| Aggregate Fraction | Tin Weight (M_1_) | Tin + Dry Soil/Rocks | Dry Soil/Rocks weight | *WS_frac_* | SMD | *MWD_frac_* | Mean Weight Diameter (MWD) | Corrected sample mass | Initial sample weight: |
| --- | --- | --- | --- | --- | --- | --- | --- | --- | --- |
| µm | g | g | g |  | µm |  | µm | g | g |
| Rock | 63.36 | 64.55 | 1.19 |  | - | - | 823 | 48.78 | 49.97 |
| >2000 | 55.42 | 64.55 | 9.13 | 0.1872 | 2000 | 374.40 |  |  |
| 250-2000 | 55.05 | 71.86 | 16.81 | 0.3446 | 1125 | 387.68 |  |  |
| 53-250 | 55.15 | 72.92 | 17.77 | 0.3643 | 151.5 | 55.19 |  |  |
| <53 |  |  | 5.07 | 0.1039 | 53 | 5.51 |  |  |

Corrected sample mass = original sample mass – total s/g mass = 49.97 – 1.19 = 48.78

*WS_frac_*(>2000) = 9.13 / 48.78 = 0.1872

*MWD_frac_* = ([WSA_frac_] x [SMD])

MWD = ∑ *MWD_frac_*

**References**

Soil Science Society of America. 1997. Glossary of soil science terms. Rev. ed. Soil Sci. Soc. Am., Madison, WI.

Mikha, M. M., & Rice, C. W. (2004). Tillage and Manure Effects on Soil and Aggregate-Associated Carbon and Nitrogen. Soil Sci. Soc. Am. J., 68.

Nimmo, J. R., & Perkins, K. S. (2002). 2.6 Aggregate Stability and Size Distribution. In Methods of Soil Analysis (pp. 317–328). John Wiley & Sons, Ltd. https://doi.org/10.2136/sssabookser5.4.c14

# **Laboratory Method 3: KSSL Wet Aggregate Stability**

**Air-Dry, 2 to 1 mm, 2- to 0.5-mm Aggregates Retained**

Adapted from USDA NRCS. (2023). [Kellogg Soil Survey Laboratory Methods Manual, Soil Survey Investigations Report No. 42, Version 6.0, Part 1: Current Methods (usda.gov)](https://www.nrcs.usda.gov/sites/default/files/2023-01/SSIR42.pdf)

Method code: KSSL_WSA

**Application**

An aggregate is a group of primary soil particles that cohere to each other more strongly than to other surrounding soil particles (Soil Science Society of America, 1997). Disaggregation of soil mass into aggregates requires the application of a disrupting force. Aggregate stability is a function of the capacity of cohesive forces among particles to withstand the applied disruptive force. The analysis of soil aggregation can be used to evaluate or predict the effects of various agricultural techniques, such as tillage, addition of organic matter, and erosion by wind and water (Nimmo and Perkins, 2002). The measurement can serve as a predictor of the potential for infiltration and soil erosion. This method provides a measure of aggregate stability from a disruption of initial air-dry aggregates by abrupt submergence followed by wet sieving. This procedure was developed by the soil survey field offices of the Natural Resources Conservation Service.

**Summary of Method**

This method measures the retention of air-dry aggregates (2 to 1 mm) on a 0.5-mm sieve after sample has been submerged in reverse osmosis (RO) water overnight and then agitated.

**Interferences**

- Air bubbles in the sieve can create tension in the water and reduce the percentage of aggregates that are retained on the 0.5-mm sieve.
- Variation in the moisture content of air-dry soils can affect results.
- A correction is needed for the sand particles >0.5 mm that are resistant to dispersion in sodium hexametaphosphate.

**Safety**

If ovens are used, hot surfaces are a hazard. Follow standard laboratory safety precautions.

**Collecting, Handling, and Processing of Soil Samples**

Ensure the soil sample collection bags are properly labeled. Keep collected samples cool and out of sunlight while in the field (preferably in a cooler). Do not dry or freeze.

After returning from the field, place samples in a cool area (refrigerator).

If shipping samples, do so in field-moist, undisturbed state, or process samples to <8 mm in the field and ship samples in moist or air-dried state. Pack samples so that they are not crushed or compacted during shipment. Double bag each sample and follow all applicable APHIS regulations. Ship using a 2- or 3-day service.

To process, air-dry in a cool location. Take a natural fabric (NF) subsample from the air-dried soil sample. Follow “Preparation of Subsample” section for guidance on preparing NF subsample for analysis.

**Equipment**

- Bowls, Rubbermaid or equivalent, 1800 mL
- Electronic balance, ±0.01-g sensitivity and 500-g capacity
- Sieves, square hole
- 0.5 mm, stainless steel, no.35, 125-mm diameter, 50-mm height
- 1 mm, brass, 203-mm diameter, 50-mm height
- 2 mm, brass, 203-mm diameter, 50-mm height
- Oven, 110 °C
- Camping plate, Coleman (Peak 1 Model 8553-462) or equivalent, stainless steel, 152-mm diameter
- Aluminum foil dish, 57-mm diameter x 15-mm deep, lifting tab
- Syringe, any size

**Reagents**

- Reverse osmosis (RO) water
- Sodium hexametaphosphate solution. Dissolve 35.7 g of sodium hexametaphosphate solution (Na4P2O7) and 7.94 g of sodium carbonate (Na2CO3) in 1 liter of RO water. Calgon water softener may be used as an alternative to Na4P2O7.

**Procedure**

**Preparation of Subsample**

- After the soil sample is air-dried, take a representative NF subsample large enough to ensure 3 g of 2- to 1-mm material is available for analysis.
- Assemble a 2-mm sieve on top of a 1-mm sieve. Crush sample so that it passes through the 2-mm sieve with minimal reduction in size. Use as little force as possible. Crush by hand at first and then with a mortar and pestle to sieve entire NF subsample.
- Return the material that is retained on the 1-mm sieve to the soil sample container. Discard the remaining material.

**Analysis**

- When ready for analysis, sieve the material again with the 1-mm sieve to remove dust and other small particles.
- Weigh out an approximately 3.00-g (±0.05 g) sample of the 2- to 1-mm material and place in an aluminum foil dish.
- Place a 0.5-mm sieve in plastic bowl. Fill bowl with water to a height of 20 mm above the base of the screen. Remove air bubbles on the screen with a syringe.
- Sprinkle the 3.00-g sample (2 to 1 mm) onto the submersed 0.5-mm sieve. As much as possible, distribute the sample so aggregates form an even layer and do not overlap.
- Allow sample to sit overnight.
- Agitate the sample by raising and lowering the sieve in the bowl of water 20 times within 40 seconds. On the upward strokes, drain sieve but do not raise so high that air enters beneath the sieve.
- Remove sieve from bowl, place on Coleman plate, and dry in oven for 2.0 to 2.5 hours at 110 °C. During the drying process, the plate retains the soil material that drops through the sieve.
- Remove the sample from the oven. Weigh and record the weight of the sieve, plate, and sample (W1).

If no sand (>0.5-mm particles) is present—

- Brush sample from the sieve and plate.
- Weigh and record weight of the sieve and plate (W2).
- Calculate the aggregates retained (WR) on 0.5-mm sieve, WR = W1 − W2.

If sand (>0.5-mm particles) is present (and no particle-size data is available)—

- Brush sample from plate and sieve. Discard the sample material on plate, but retain the sample fraction from the 0.5-mm sieve.
- Weigh and record weight of the sieve and plate (W2).
- Calculate the aggregates retained (WR) on 0.5-mm sieve, WR = W1 − W2.
- Return the sample fraction from the 0.5-mm sieve to the sieve.
- Disperse sample fraction returned to the sieve with sodium hexametaphosphate solution. Alternatively, place 3 g of Calgon in plastic bowl and stir until dissolved and then use to disperse sample fraction.
- Place the 0.5-mm sieve and sample with sodium hexametaphosphate solution (or Calgon) so that the solution line is 35 mm above the base of the screen.
- Gently triturate the dispersing solution with fingers to remove the soft <0.5-mm material adhering to the ≥0.5-mm sand particles.
- Remove sieve from sodium hexametaphosphate solution (or Calgon) and rinse with RO water until all sodium hexametaphosphate solution (or Calgon) has passed through the sieve and only the sand (>0.5-mm particles) is left on sieve.
- Place sieve on Coleman plate, place in oven, and dry for 2.0 to 2.5 hours at 110 °C.
- Remove sample from oven. Record the weight of sieve, plate, and sand (W3).
- Discard sample, and brush sieve and plate. Record the weight of the sieve and plate (W4).
- Determine sand weight, SW = W3 − W4. Sand weight will be used as the correction in the final calculation.

**Cleanup**

- After all samples are processed, thoroughly wash sieves, bowls, and plates with RO water, especially the sieves in which sodium hexametaphosphate solution (or Calgon) was used.
- Store inverted to dry.

**Calculations**

Aggregates (%) = {{(WR−SW) / {[IW / (AD/OD)] − Sw}} x 100

where:

IW = Initial sample weight (g)

WR = Total weight of aggregates retained on 0.5-mm sieve (g)

SW = Weight of 2- to 0.5-mm sand (g)

AD/OD = Air-dry/oven-dry ratio (If not available, use 1.00.)

**Report**

Report aggregate stability as a percentage of aggregates (2 to 0.5 mm) retained after wet sieving. Do not report determinations if the 2- to 0.5-mm primary particles are ≥50 percent of the 2- to 1-mm sample.

**References**

Soil Science Society of America (1997). Glossary of soil science terms. Rev. ed. Soil Sci. Soc. Am., Madison, WI.

Nimmo, J. R., & Perkins, K. S. (2002). 2.6 Aggregate Stability and Size Distribution. In Methods of Soil Analysis (pp. 317–328). John Wiley & Sons, Ltd. https://doi.org/10.2136/sssabookser5.4.c14

# **Laboratory Method 4: CO2 Respired, 4-Day Incubation**

Moebius-Clune, B.N., D.J. Moebius-Clune, B.K. Gugino, O.J. Idowu, R.R. Schindelbeck, A.J. Ristow,

H.M. van Es, J.E. Thies, H. A. Shayler, M. B. McBride, D.W. Wolfe, and G.S. Abawi, 2016.

Comprehensive Assessment of Soil Health – The Cornell Framework Manual, Edition 3.0, Cornell

University, Geneva, NY. Available at <https://bpb-us-e1.wpmucdn.com/blogs.cornell.edu/dist/7/9922/files/2021/11/CASH-Standard-Operating-Procedures-030217final-u8hmwf.pdf>

Method code: Soil_Respiration

**Application**

Biota play an important role in many processes that occur in soil. Soil microbes, through their metabolic pathways, cycle plant nutrients from unavailable forms to available forms. Microbes also contribute to the formation of soil structure, or the formation of mineral and organic matter into aggregates. The space between these soil aggregates allows soil to absorb water and cycle air, which are important for healthy, productive soils. A robust, active community of soil microbes can improve the response of plants to water stress in fluctuating environments, and it can suppress excessive growth of disease-causing organisms in plants and aid in resistance of plants to disease.

**Summary of Method**

A 4-day incubation period is used to measure output of carbon dioxide (CO2)—a waste product of respiration—by chamber alkali trap respirometry. An air-dried soil sample is rewet in a jar. Microbial activity, which has been very low in the sample due to its dry state, resumes rapidly as the soil rehydrates. CO2 diffuses out of the soil sample and is prevented from escaping the jar by an airtight seal. A small beaker of potassium hydroxide (KOH), which serves as a trap for the CO_2_, is sealed in the jar with the soil sample. The properties of the KOH solution change in proportion to the amount of CO2 trapped. To determine the amount of CO2 absorbed by the alkali trap, the electrical conductivity (EC) of the KOH solution is measured at the end of the 4-day incubation period. The amount of CO2 trapped is calculated from the change in EC. This calculation is used to estimate the amount of CO2 evolved (released) from the sample and then by inference, the metabolic activity of the microbial community in the sample.

**Interferences**

- It is best practice to purchase 0.5 M KOH as a premade solution. KOH pellets absorb water and carbon dioxide from the air after opening; thus, the precision of solutions prepared in the laboratory is less reliable.
- Because KOH absorbs CO2 from the air, which decreases its conductivity, minimize the amount of time KOH is open to ambient air and do not reuse dispensed KOH.
- Growth of observable mold or fungus triggers a rerun of the sample.

**Safety**

KOH is caustic, and it has a delayed reaction when it comes in contact with skin. Wear gloves and safety glasses while handling KOH.

**Collecting, Handling, and Processing of Soil Samples**

Ensure the soil sample collection bags are properly labeled. Keep collected samples cool and out of sunlight while in the field (preferably in a cooler).

After returning from the field, place samples in a cool area (refrigerator). Do not dry or freeze.

Double bag each sample, and follow all applicable APHIS regulations. Ship using a 2- or 3-day service.

Process samples by air-drying in a cool location and sieving through an 8-mm screen.

Follow the established protocol for laboratory analysis.

**Equipment**

**Pre-setup**

- Soil samples (sieved to 8 mm, air-dried to constant mass)
- Jars with lids (wide mouth, 1 pint, Ball mason jars, standard 2-part lids)
- Labeling tape and permanent marker
- Filter paper (55 mm)
- Weighing paper (3” x 3” or 4” x 4”)
- Pre-perforated aluminum weigh boats (57 mm, 9 holes in bottom)
- Standard teasing needles, straight point
- Trap assembly (10-ml beaker taped onto plastic tripod, or ‘pizza stool’)
- Jar racks (25 place)

**Incubation Initiation**

- Distilled, deionized water (ddH2O)
- 0.5 M KOH (kept covered)
- 500-ml beakers
- Large beaker or watch glass (cover for beaker of KOH)
- 10-ml pipettor
- 10-ml pipette tips

**Post-incubation Reading**

- Incubated samples in jars
- EC meter with computer
- Kimwipes® and cut strips of filter paper
- Container for spent KOH
- Washtub with distilled water (dH2O) for trap assemblies
- Hydrochloric acid (HCl) to neutralize KOH
- pH test strips or handheld pH meter
- Stir plate and stir bar

**Method**

**Preparation of Sample Jar**

Recommended batch size.—42 sample jars (2 replicates per soil sample), 5 blank jars, and 2 quality control (QC) sample jars; total of 49 jars per batch. Begin sample batch with a blank, and place the remaining 4 blanks 10 sample jars apart, excluding QC sample jars. Analyze the QC samples after the 2nd replicate of the 5th soil sample and after the 2nd replicate of the 20th soil sample.


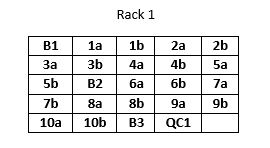

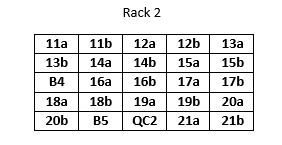


**Figure 1.—Example of sample, blank, and QC orientation in Ball jar racks from standard operating procedures by Moebius-Clune et al.**

- Pre-perforate aluminum weigh boats with a teasing needle. Place nine holes in the center, making a uniform 3 x 3 square.
- Label a set of jars for the samples that will be weighed out. Ensure 25-place jar racks are available for use.
- Add two filter papers, each offset from the other, to the bottom of each jar. If needed, use long forceps to adjust placement of filter papers.
- Weigh 20.00 g <8-mm sieved, air-dried soil into a pre-perforated aluminum weigh boat that has a weigh paper placed under it. Pre-weighed soil may be stored in covered jars a few days ahead of incubation setup.
- Using long forceps, place the weigh boat into the jar. If any soil remains on the weigh paper, tap remaining soil into the aluminum weigh boat after placing weigh boat in the jar.
- Place one trap assembly (10-ml beaker stuck to plastic tripod, or pizza stool) into each respirometer jar. Press the legs of the stool down into the soil sample to allow it to firmly stand and to better conform the shape of the flat-bottomed weigh boat to the slightly domed bottom of the jar.

**Incubation Setup**

- Dispense KOH and ddH2O into clean, dry 500-ml beakers. Cover the KOH beaker with a 1-L beaker or a watch glass to minimize excessive exposure to air.
- Work with a few jars at a time (i.e., a set of 11 jars that includes 10 sample jars and a blank) to minimize contact time of air to KOH.
- Note the date and time setup begins. Read samples at the same time of day on day four of the incubation period.
- Add 9 ml of 0.5 M KOH to trap beakers in jars.
- Using a different 10-mL pipette tip, add 7.5 ml ddH2O into each jar. Avoid contact with the KOH trap. Dispense water close to the jar wall to avoiding dispensing directly into the soil.
- Place a lid on the jar as soon as both reagents are added. Minimize the amount of time the jar is open.
- Close the jars securely with the screw-top rings. Screw on tight enough to make an airtight seal.
- Carefully move closed jars to a jar rack to avoid spilling KOH from the trap assemblies. Jar racks should be labeled with information relevant to the sample set to easily identify for retrieval (i.e., sample number range, batch identification numbers, start/stop date and times).
- Discard leftover KOH into a used KOH container. Do not return leftover KOH to stock solution.
- Incubate for 4 days. Avoid major temperature fluctuations. Use an incubator at ambient temperature, if available.

**Measurement after Incubation**

- Ensure the EC meter is properly calibrated according to manufacturer’s instructions.
- Recommendation.—Fill a dry beaker with KOH from a “used KOH” container, and check that the EC meter probe is working properly before beginning sample readings.
- Fill one beaker with tap water for rinsing the EC meter probe, and fill one beaker with ddH2O for final cleaning of the probe.
- If using a computer to record measurements, ensure that the computer is on and ready to receive data from the EC meter.
- Retrieve the rack of jars from the shelf, and move the rack to the bench.
- Remove a numbers of jars in order, as when setting up the incubation.
- Remove the rings from the number of jars removed from the rack. Leave the flat lids in place.
- Remove the flat lid from first jar. Place the probe into the trap beaker, briefly and gently stir with the probe, and then let the probe rest at the bottom of the trap beaker.
- Wait for the reading to stabilize and then record the EC value.
- Between samples, blot the probe dry with a Kimwipe and a strip of filter paper. Continue reading samples as described in steps above. If there is a pause between measurements, make sure no KOH is allowed to dry on the EC meter probe.
- If reading sets of 11 or 12 jars, avoid letting jars sit open for long periods.
- When all the samples are read, rinse the probe well in the beaker of tap water and then in the beaker of ddH2O. Set the probe onto a paper towel to dry.
- Make note of any duplicates that deviate by 5 percent, and flag them for rerunning. Visually inspect jars for growth of visible mold or fungus, which triggers a rerun.

**Cleanup**

- Empty the waste from KOH trap beakers into a used KOH container, and hold for neutralization.
- Place used KOH container on stir plate in a fume hood. Place a stir bar in the container, and turn stir function on at low to moderate speed. Wear gloves and eye protection.
- Neutralize with HCl while stirring, and test with a litmus paper strip or a handheld pH meter.
- When neutral, autoclave in accordance with APHIS regulations, if applicable, and dispose of down the drain with copious quantities of tap water.
- Separate trap beakers from pizza stools. Set beakers into a tub of distilled water to soak overnight before cleaning. Set pizza stools aside to be cleaned and reused.
- Empty contents of respiration jars into waste bin.
- Wash and rinse well all glassware in tap water. Rinse several times with distilled water, and then rinse with ddH2O.
- Allow glassware to fully dry. Inspect glassware. If any residue remains after drying, wash again and rinse well. Residue may interfere with further usage.
- Power down computer and EC meter, if needed.

**Calculation**

To manually calculate respiration rate—

The trap capacity for CO2 absorption is known based on its volume and concentration. When fully saturated, 0.5 M KOH can absorb sufficient CO2 to become 0.25 M potassium carbonate (K2CO3). (In practice, the absorption would likely become fairly inefficient when close to the endpoint, but the amount used is calibrated so that this situation is rare.) One mole KOH can accommodate one-half mole CO2 (accumulates as carbonate [CO3 2-] when trapped). So 9 ml of 0.5 M KOH can accommodate 0.009 L * 0.25 mol/L * 44.01 g/mol * 1000 mg/g = 99.025 mg CO2. Of this theoretical total trap capacity of 99.025 mg CO2, some fraction is actually absorbed. The proportion of the total trap capacity that is absorbed is equivalent to the proportion of the total conductivity drop that would be observed (between the EC of the ‘raw’ 0.5 M KOH and the EC of 0.25 M K2CO3) if the trap was saturated, which is the total actually observed. To restate—the difference between the measured EC for a sample (or blank) and the EC of the ‘raw’ KOH is a quantity that can be called the ‘observed EC drop.’ This quantity is some fraction of the total possible drop, which can be called ‘full capacity EC drop.’ Dividing the ‘observed EC drop’ by the ‘full capacity EC drop’ gives a fraction that is equivalent to the fraction of the total trap capacity for CO2 absorption that is actually used. So:

P = ((ECraw – ECsample)/(ECraw – ECsat))

Given a constant temperature for all samples,

ECraw = Electrical conductivity of pure 0.5 M KOH

ECsat = Electrical conductivity of 0.25 M K2CO3

ECsample = Electrical conductivity of the trap associated with a particular sample

P = Proportion of the trap capacity for CO2 absorption that is actually used

P x (trap capacity in mg) = amount of CO2 in mg absorbed by the trap in question

**Report**

Report CO2 absorbed in mg.

**References**

Moebius-Clune, B.N., D.J. Moebius-Clune, B.K. Gugino, O.J. Idowu, R.R. Schindelbeck, A.J. Ristow, H.M. van Es, J.E. Thies, H. A. Shayler, M. B. McBride, D.W. Wolfe, and G.S. Abawi, 2016. Comprehensive Assessment of Soil Health – The Cornell Framework Manual, Edition 3.0, Cornell University, Geneva, NY. Available at [CASH-Standard-Operating-Procedures-030217final-u8hmwf.pdf (bpb-us-e1.wpmucdn.com)](https://bpb-us-e1.wpmucdn.com/blogs.cornell.edu/dist/7/9922/files/2021/11/CASH-Standard-Operating-Procedures-030217final-u8hmwf.pdf)

# **Laboratory Method 5: Soil Enzymes**

##### **Overview**

The four enzymes to be analyzed for the science of soil health are:

- β-glucosidase (BG) that is involved in the C-cycle (Eivazi and Tabatabai, 1988; Deng and Popova, 2011)
- N-acetyl-β-D-glucosaminidase (NAG) that is involved in both the C-cycle and N-cycle (Parham and Deng, 2000; Deng and Popova, 2011)
- Phosphomonoesterases (acid/alkaline phosphatase; Pase) that are involved in the P-cycle (Eivazi and Tabatabai, 1977; Acosta-Martínez and Tabatabai, 2011)
- Arylsulfatase (AS) that is involved in the S-cycle (Tabatabai, 1970; Klose et al., 2011).

The methods are traditional, bench-scale assays. If a microplate reader is available, the methods can be adapted for use of the instrument (Deng et al., 2011). The microplate reader method allows for simultaneous analysis of multiple enzymes using a small quantity of soil. Before switching to a microplate reader method, ensure that it provides equivalent values as compared to the bench-scale assays.

**Collecting, Handling, and Processing of Soil Samples**

Ensure the soil sample collection bags are properly labeled. Keep collected samples cool and out of sunlight while in the field (preferably in a cooler).

After returning from the field, place samples in a cool area (refrigerator). Do not dry or freeze.

If shipping samples, double bag each sample and follow all applicable APHIS regulations. Ship using a 2- or 3-day service.

Process samples by air-drying in a cool location and sieving through a 2-mm screen.

Biological activity of air-dry samples is low during storage.

## **ß-Glucosidase**

Method code: Bglucosidase

Application

Soil enzymes are important to the biochemical functions of organic matter decomposition and serve as catalysts in reactions necessary for the life processes of organic wastes, organic matter formation, and nutrient cycling (Das and Varma, 2011). These enzymes include amylase, arylsulphatases, ß-glucosidase, cellulose, chitinase, dehydrogenase, phosphatase, protease, and urease released from plants, animals, organic compounds, microorganisms, and soils. ß-glucosidase is a common and predominant enzyme in soils (Eivazi and Tabatabai, 1988; Tabatabai, 1994). Glucosidase is involved in the hydrolysis and biodegradation of ß-glucosidase in plant debris decomposing in the ecosystem. Its final product is glucose, which is an important C energy source for life of microbes in the soil (Esen, 1993). The ß-glucosidase enzyme is sensitive to changes in pH and soil management practices; thus, it is useful as an indicator of soil quality (Acosta-Martinez and Tabatabai, 2000; Madejon et al., 2001; Das and Varma, 2011). An enzyme assay, such as that for ß-glucosidase, reflects potential activity. It does not represent true in situ activity levels; therefore, it should be viewed as an index.

Summary of Method

A 1-g sample is treated with the Modified Universal Buffer (MUB) and p-nitrophenyl-β-D-glucopyranoside (PNG) and incubated for 1 hour at 37 °C. After incubation, calcium chloride (CaCl_2_) and THAM solution (pH 12) are added. A 10-mL sample is then pipetted, filtered, and analyzed. Data are reported as mg p-nitrophenol per kg oven-dry soil per h.

Interferences

- The ß-glucosidase assay can be determined on both air-dry and field-moist samples. On a routine basis, air-dried samples are easier to handle and do not require immediate analysis.
- ß-glucosidase activity in the sample declines with air-drying, but that does not affect the ranking of soil treatments within a soil type or the ranking across different soil types (Bandick and Dick, 1999).
- Because p-nitrophenol is light sensitive, read samples as soon as possible after filtration.

Safety

- Wear disposable gloves that have an appropriate polymer rating for chemical resistance, safety glasses and/or a face shield, and a lab coat or apron when preparing reagents, especially concentrated acids and bases.
- Thoroughly wash hands after handling reagents.
- Use safety showers and eyewash stations to dilute spilled acids and bases.
- Use sodium bicarbonate (CAS #497-9-8) and water to neutralize and dilute spilled acids.
- Dispense concentrated acids and bases in a fume hood.
- Always work in a fume hood when handling p-nitrophenol solutions.

Equipment

- Electronic balance, ±1.0-mg sensitivity
- Volumetric flasks, acid washed, 50 mL, 100 mL, 1000 mL
- Plastic bottle, amber, 1000 mL
- Funnel, 60° angle, long stem, 50-mm diameter
- Filter, 0.45 µm
- Pipettes, electronic digital, 2500 µL and 10 mL, 2500-µL and 10-mL tips
- Syringe filters, 0.45 µm, Whatman
- Centrifuge tubes, 50 mL, polypropylene
- Cuvettes, plastic, 4.5 mL, 1-cm light path
- Centrifuge
- Disposable pipettes
- Incubator or water bath, 37 °C
- Spectrophotometer, UV visible
- Vortex mixer
- Syringes, 1 mL

Reagents

- Reverse osmosis, deionized water (RODI), ASTM Type I grade reagent water
- Hydrochloric acid solution, 0.1 M

Components.—hydrochloric acid (HCl) (CAS #7647-01-0) concentrated, 12 N, RODI water

Preparation.—In a 1-L flask, add 8.33 mL HCl to 1L RODI water.

- Sodium hydroxide solution, 1 M

Components.—sodium hydroxide (NaOH) (CAS #1310-73-2), RODI water

Preparation.—In a 1-L volumetric flask, add 40.00 g NaOH to 1 L RODI water.

- MUB stock solution

Components.—THAM 2-amino-2 (hydroxymethyl)-1-3-propanediol (NH_2_C(CH_2_OH)_3_) (CAS #77-86-1); maleic acid (HO_2_CCH=CHCO_2_H) (CAS #110-16-7); citric acid (C_6_H_8_O_7_) (CAS #77-92-9); boric acid (H_3_BO_3_) (CAS #10043-35-3); sodium hydroxide solution, 1 M

Preparation.—Dissolve the following in 488 mL of sodium hydroxide solution in a 1-L volumetric flask:

- 12.10 g THAM,
- 11.60 g maleic acid,
- 14.00 g citric acid, and
- 6.30 g boric acid.

Dilute to 1 L with RODI. Store in refrigerator.

- MUB 6.0, working solution (pH 6.0)

Components.—MUB stock solution, 0.1 M HCl solution, RODI water

Preparation.—Place 200 mL MUB stock solution in a 500-mL beaker that has a magnetic stir bar. Place the beaker on a magnetic stirrer. While stirring, slowly add 0.1 M HCl to the MUB stock solution until the pH reaches 6.0. Transfer acidified solution to 1-L volumetric flask, and adjust the volume to 1 L with RODI.

- PNG solution, 0.05 M

Components.—p-nitrophenyl-β-D-glucopyranoside (PNG) (C_12_H_15_NO_8_) (CAS #2492-87-7), MUB 6.0 working solution

Preparation.—In a 100-mL volumetric flask, dissolve 1.506-g PNG in about 80 mL MUB 6.0. After the PNG is dissolved, bring the solution to a volume of 100 mL with MUB 6.0. Use within 5 days. Store in refrigerator (stable several days at 4 °C).

- Calcium chloride solution, 0.5 M

Components.—calcium chloride dihydrate (CaCl_2_•2H_2_O) (CAS #10035-04-8), RODI water

Preparation.—In a 1-L volumetric flask, dissolve 73.5 g CaCl_2_•2H_2_O in about 700 mL RODI. Bring to volume with RODI.

- Sodium hydroxide solution, 0.5 M

Components.—sodium hydroxide (NaOH) (CAS #1310-73-2), RODI water

Preparation.—In a 1-L volumetric flask, dissolve 20 g NaOH in about 700 mL RODI. After the NaOH is dissolved, bring to volume with RODI.

- THAM 10 solution, 0.1 M

Components.—THAM 2-amino-2 (hydroxymethyl)-1-3-propanediol (NH_2_C(CH_2_OH)_3_) (CAS #77-86-1), RODI water

Preparation.—In a 1-L volumetric flask, dissolve 12.20 g THAM in about 800 mL RODI. After the THAM is dissolved, bring to volume with RODI (pH ≈10).

- THAM 12 solution, 0.1 M

Components.—sodium hydroxide solution, 1 M; THAM 2-amino-2 (hydroxymethyl)-1-3-propanediol (NH_2_C(CH_2_OH)_3_) (CAS #77-86-1); RODI water

Preparation.—Dissolve 12.20 g THAM in about 800 mL RODI. After the THAM is dissolved, adjust the pH to 12 by slowly adding 0.5 M sodium hydroxide. Bring to volume with RODI.

- Reagent A: p-nitrophenol stock standard solution

Components.—p-nitrophenol (C_6_H_5_NO_3_) (CAS #100-02-7), RODI water

Preparation.—In a fume hood, dissolve 1.00 g p-nitrophenol in about 700 mL RODI in a 1-L volumetric flask. After the p-nitrophenol is dissolved, bring to volume with RODI. Store at 4 °C in amber bottle. Use within 30 days.

- Reagent A1: p-nitrophenol working standard solution

Components.—reagent A, RODI water

Preparation.—In a fume hood, add 1 mL reagent A to 100-mL volumetric flask. Bring to volume with RODI. Mix well. Store at 4 °C in amber bottle. Use within 30 days

- Reagents A2-A6: Standard p-nitrophenol calibration and verification solutions

Components.—reagent A1; THAM 12, 0.1 M; calcium chloride solution, 0.5 M; RODI water

Preparations.—Prepare in 50-ml centrifuge tubes. Refer to table 1 for concentrations and dilutions. Bring to volume with RODI water. Cap tubes and then shake to mix.

**Table 1: Reagents A2-A6—Standard p-Nitrophenol Calibration and Verification Solutions**

| **Reagent** | **Final amount in 10 ml** (µg) | **p-nitrophenol working standard solution**  (reagent A1) (mL) | **CaCl_2_** | **THAM 12** | **RODI water** |
| --- | --- | --- | --- | --- | --- |
| A2 | 50.0 | 5 | 1 mL | 4 mL | Dilute with RODI water to volume |
| A3 | 40.0 | 4 |
| A4 | 30.0 | 3 |
| A5 | 20.0 | 2 |
| A6 | 10.0 | 1 |
| Blank | 0 | 0 |

Procedure

- - Weigh 1 g (±0.03 g) of <2-mm, air-dry soil into disposable centrifuge tubes. Label the odd numbered tubes **“**control**”** and the even-numbered tubes **“**treatment**.”**
- Preheat MUB 6.0 and substrate to 37 °C prior to assay. Warm CaCl_2_, THAM 12, and THAM 10 dilution reagent to room temperature.
- In a fume hood, add 4 mL MUB 6.0 to all control and treatment tubes.
- Add 1 mL of PNG solution to the treatment tubes only. Start the 60-minute timer immediately after adding the PNG. Cap all tubes and mix in vortexer.
- Incubate all samples at 37 °C for 1 hour.
- Add 1 mL of 0.5 M CaCl_2_ and 4 mL of 0.1 THAM 12 to all tubes and swirl.
- Add 1 mL PNG solution to control tubes and mix in vortexer. Remove caps. Let samples settle 5 minutes before filtering.
- Draw up sample into a 10-mL syringe and attach a 0.45-µm filter. Filter sample directly into cuvette. Because p-nitrophenol is light sensitive, read samples as soon as possible after filtration. Cap the samples waiting to be read.
- Prepare calibration curve plotting absorbance at 410 nm versus amount of p-nitrophenol.
- Record the amounts of p-nitrophenol. Read the samples in control/treatment pairs.
- If readings exceed high standard, dilute with THAM 10. Cap and swirl. Transfer to cuvettes, and record the amount of p-nitrophenol.

Calculations

mg p-nitrophenol / kg oven-dry soil / h = B x [(D_T_ x A_T_ / Wt_T_) − (D_C_ x A_C_ / Wt_C_)] / T

where:

A_T_ = Amount of p-nitrophenol in treatment sample (µg)

A_C_ = Amount of p-nitrophenol in control sample (µg)

Wt_T_ = Weight of treatment sample (g)

Wt_C_ = Weight of control sample (g)

D_C_ = Dilution factor for control sample

D_T_ = Dilution factor for treatment sample

T = Incubation time (h)

B = Air-dry/oven-dry ratio (method 3D1) or field-moist/oven-dry ratio

Note: µg p-nitrophenol / g oven-dry soil / hour = mg p-nitrophenol / kg oven-dry soil / hour

Report

Report ß-glucosidase activity as mg p-nitrophenol / kg oven-dry soil / h.

## **Acid Phosphatase**

Method code: AcidPhosphatase

**Introduction to Assay for Acid Phosphatase Enzyme**

Soil enzymes are important to the biochemical functions of organic matter decomposition, and they are catalysts for reactions necessary for the life processes of organic waste, organic matter formation, and nutrient cycling (Das and Varma, 2011). Enzyme assays reflect potential activity. They do not represent true in situ activity levels; therefore, results should be viewed as an index.

**Scope and Field of Application**

Acid phosphatase is a common extracellular enzyme produced by many organisms in the soil. It removes the phosphate molecule from organic compounds, such as phospholipids and nucleic acids. Once the phosphate is cleaved, it becomes soluble and can be taken up by a cell. This is important because phosphate commonly is the limiting nutrient for microbial growth in soil.

**Principle**

The acid phosphatase assay can be determined on both air-dry and field-moist samples; however, air-dry samples do not require immediate analysis. Modified Universal Buffer (pH 6.5) is added to 1-g samples (control and treatment) and then 0.05 M p-nitrophenyl phosphate solution is added to the treatment samples only (Tabatabai and Bremner, 1969). The samples are incubated for 1 hour at 37 °C. After incubation, 0.5 M calcium chloride (CaCl_2)_ and 0.5 M sodium hydroxide (NaOH) are added to each sample. To the control samples only, p-nitrophenyl phosphate solution is added. Samples are allowed to settle for 5 minutes. A 10-mL aliquot is filtered for each sample. A calibration curve is prepared plotting absorbance at 405 nm versus amount of p-nitrophenol. If readings exceed the high standard, dilute original filtered sample with water. Data are reported as mg p-nitrophenol per kg oven-dry soil per h (Eivazi and Tabatabai, 1977).

**Interferences**

Because p-nitrophenol is light sensitive, read samples as soon as possible after filtration.

**Equipment**

- Electronic balance, ±1.0mg sensitivity
  - Volumetric flasks, acid washed, 50 mL, 100 mL, 1000 mL
  - Plastic bottles, amber, 1000 mL
  - Disposable plastic cups, 1 oz. (30 mL)
  - Pipettes, electronic digital, 2500 µL and 10 mL, 2500-µL and 10-mL tips
  - Pipette, electronic digital, 50 ml
  - Syringe filters, 0.45 µm, Whatman
  - Centrifuge tubes with lids, 50 mL
  - Cuvettes, plastic, 4.5 mL, 1-cm light path
  - Incubator or water bath, 37 °C
  - Spectrophotometer, UV visible, dual view
  - Vortexer, mini
  - Syringes, 10 mL
  - Beaker, 500 mL
  - pH meter
  - Magnetic stir bars
  - Magnetic stir plate

**Reagents**

- - Reverse osmosis, deionized (RODI) water, ASTM Type I grade reagent water
  - THAM 2-amino-2(hydroxymethyl)-1-3-propanediol (NH_2_C(CH_2_OH)_3_) (CAS #77-86-1)
  - p-nitrophenyl-phosphate disodium salt hexahydrate (O_2_NC_6_H_4_OP(O)(ONa)_2_ 6H_2_O) (CAS #333338-18-4)
  - p-nitrophenol (C_6_H_5_NO_3)_ (CAS #100-02-7)
  - Calcium chloride dihydrate (CaCl_2_•2H_2_O) (CAS #10035-04-8)
  - Hydrochloric acid (HCl) concentrated, 12 N (CAS #7647-01-0)
  - Maleic acid (HO_2_CCH=CHCO_2_H) (CAS #110-16-7)
  - Sodium hydroxide (NaOH) (CAS #1310-73-2)
  - Citric acid (C_6_H_8_O_7_) (CAS #77-92-9)
  - Boric acid (H_3_BO_3_) (CAS #10043-35-3)
  - Sodium hydroxide (NaOH) (CAS #1310-73-2)
  - Sodium hydroxide solution, 1.0 M

Components.—sodium hydroxide (NaOH), RODI water

- To a 1-L glass volumetric flask, add the following in order:
- 700 mL RODI water,
- 40.00 g NaOH, and
- fill to volume with RODI water.
- Invert to mix.
  - Hydrochloric acid solution, 0.1 N

Components.—concentrated hydrochloric acid (HCl), RODI water

- To a 100 mL glass volumetric flask, add the following in order:
- 70 mL RODI water,
- 0.833 mL concentrated HCl, and
- fill to volume with RODI water.
- Invert to mix.
  - Modified Universal Buffer (MUB) stock solution

Components.—THAM (NH_2_C(CH_2_OH)_3_), maleic acid (HO_2_CCH=CHCO_2_H), citric acid (C_6_H_8_O_7_), boric acid (H_3_BO_3_), 1 M sodium hydroxide solution

- To a 1-L glass volumetric flask, add the following in order:
- 488 mL, 1 M sodium hydroxide solution,
- 12.10 g THAM,
- 11.60 g maleic acid,
- 14.00 g citric acid,
- 6.30 g boric acid, and
- fill to volume with RODI water.
- Store in refrigerator.
  - MUB 6.5: Working solution (pH 6.5)

Components.—MUB stock solution, 0.1 N HCl solution, RODI water

- To a 500 mL glass beaker, add a magnetic stir bar. Place the beaker on a magnetic stirrer. Add the following in order:
- 200 mL MUB stock solution and
- 0.1 N HCl adjusting the pH to 6.5.
- Transfer acidified solution to 1 L volumetric flask.
- Fill to volume with RODI water.
- Invert to mix.
  - pNPP solution, 0.05 M

Components.—p-nitrophenyl-phosphate disodium salt hexahydrate (O_2_NC_6_H_4_OP(O)(ONa)_2_ 6H_2_O), MUB 6.5 solution

- To a 50 mL glass volumetric flask, add the following in order:
- 40 mL MUB pH 6.5 solution,
- 0.9275 g p-nitrophenyl phosphate disodium salt hexahydrate, and
- fill to volume with MUB pH 6.5 solution.
- Store solution in refrigerator.
- Prepare and use same week as analysis.
- Sufficient reagent for about 25 samples in duplicate (control and treatment samples)
  - Calcium chloride solution, 0.5 M

Components.—calcium chloride dihydrate (CaCl_2_•2H_2_O), RODI water

- To a 1-L glass volumetric flask, add the following in order:
- 700 mL RODI water,
- 73.5 g CaCl_2_•2H_2_O, and
- fill to volume with RODI water.
- Invert to mix.
  - Sodium hydroxide solution, 0.5 M

Components.—sodium hydroxide (NaOH), RODI water

- To a 1-L glass volumetric flask, add the following in order:
- 700 mL RODI water,
- 20 g NaOH, and
- fill to volume with RODI water.
- Invert to mix.
  - p-nitrophenol stock standard solution (reagent A)

Components.—p-nitrophenol (C_6_H_5_NO_3_), RODI water

- In a fume hood, add the following in order to a 1-L glass volumetric flask:
- 700 mL RODI water,
- 1.00 g p-nitrophenol, and
- fill to volume with RODI water (after p-nitrophenol is dissolved).
- Store at 4 °C in amber bottle.
- Use within 30 days.
  - p-nitrophenol working standard solution (reagent A1)

Components.—reagent A, RODI water

- In a fume hood, add the following in order to a 100-mL glass volumetric flask:
- 1 mL reagent A and
- fill to volume with RODI water.
- Invert to mix.
- Store at 4 °C in amber bottle.
- Use within 30 days
  - Standard p-nitrophenol calibration and verification solutions (Standards A2-A6)

Components.—reagent A1, 0.5 M calcium chloride solution, 0.5 M sodium hydroxide solution, RODI water

- Refer to table 1 for mixing instructions.
- Refer to table 2 for concentrations.
- Prepare in 50-mL disposable centrifuge tubes.
- Cap and shake tubes to mix.

**Table 1: Standards A2-A6—Preparation**

| **Standard p-nitrophenol calibration and verification solutions** | | | | |
| --- | --- | --- | --- | --- |
| **Standard** | **Reagent A1**  (mL) | **0.5 M CaCl_2_** | **0.5 M NaOH** | **RODI**  **water** |
| A6 | 5 | 1 mL | 4 mL | 0 |
| A5 | 4 |  |  | 1 |
| A4 | 3 |  |  | 2 |
| A3 | 2 |  |  | 3 |
| A2 | 1 |  |  | 4 |
| Blank | 0 |  |  | 5 |

**Table 2: Standards A2-A6—Amount in 10 ml of Solution**

| **Standard p-nitrophenol calibration and  verification solutions** | |
| --- | --- |
| **Standard** | **Final amount** (µg) |
| A6 | 50.0 |
| A5 | 40.0 |
| A4 | 30.0 |
| A3 | 20.0 |
| A2 | 10.0 |
| Blank | 0 |

- - Toluene

Optional application that inhibits microbial growth during long incubation periods. It is not advised to use toluene for a 1-hour incubation period (Dr. Richard Dick, Ohio State University, June 2017).

**Health and Safety**

Wear disposable gloves that have an appropriate polymer rating for chemical resistance, safety glasses and/or face shield, and a lab coat or apron when preparing reagents, especially concentrated acids and bases. Dispense concentrated acids and bases in a fume hood. Thoroughly wash hands after handling reagents. Use safety showers and eyewash stations to dilute spilled acids and bases. Use sodium bicarbonate and water to neutralize and dilute spilled acids.

Always work in a fume hood and wear gloves when handling p-nitrophenyl phosphate, p-nitrophenol solutions, and toluene, which is highly flammable.

Follow the manufacturer's safety precautions when using the spectrophotometer.

**Preparation of Samples**

Air-dry the field samples at 30 to 35 °C, crush, and sieve to <2 mm. The weight of the air-dry soil sample remains relatively constant. Biological activity of air-dry samples is low during storage.

**Procedure**

- - Weigh 1 g (±0.03 g) of <2-mm, air-dry soil into 50-mL tubes. Label odd-numbered tubes “control” and even-numbered tubes “treatment.”
  - Prior to assay, preheat MUB 6.5 and substrate to 37 °C. Warm CaCl_2_ and NaOH
    0.5 M to room temperature.
  - Working in a fume hood, add 4 mL MUB 6.5 to all tubes (control and treatment).
  - Add 1 mL pNPP solution to the treatment, or even numbered, tubes only. Cap all tubes (control and treatment). Immediately start the 60-minute timer. After starting timer, use vortexer to mix all tubes and then place tubes in a water bath or an incubator.
  - Incubate all tubes (control and treatment) at 37 °C for 1 hour. After 1 hour, remove from water bath or incubator.
  - Add 1 mL 0.5 M calcium chloride (CaCl_2_) solution and 4 mL 0.5 M sodium hydroxide (NaOH) solution to all tubes. Swirl the tubes. Note: The CaCl_2_ and NaOH reagents may be combined in a ratio of 1:4. Combine 10 to 15 minutes before use. Add 5 mL of the combined reagent to each tube.
  - Add 1 mL pNPP solution to control, or odd numbered, tubes and vortex all tubes (control and treatment). Remove caps. Let samples settle 5 minutes before filtering.
  - Draw up sample into a 10-mL syringe and attach a 0.45-µm filter. Filter sample directly into disposable cup. Transfer filtrate into cuvettes. Because p-nitrophenol is light sensitive, read sample as soon as possible after filtration. Cover the samples waiting to be read.
  - Set the spectrophotometer to read at 405 nm. Auto zero with the calibration blank. Sample concentration is calculated from the regression equation.
  - Record the amount of p-nitrophenol. Read the samples in control/treatment pairs.
  - If readings exceed the 50-µg standard for p-nitrophenol, dilute an aliquot of the filtrate with RODI until the absorbance readings are within the limits of the calibration curve. Dilute an aliquot from both the control and treatment tubes for samples that exceed the standard.

**Calculations**

mg p-nitrophenol / kg oven-dry soil / h = B x [(D_T_ x A_T_ / Wt_T_) − (D_C_ x A_C_ / Wt_C_)] / T

where:

A_T_ = Amount of p-nitrophenol in treatment sample (µg)

A_C_ = Amount of p-nitrophenol in control sample (µg)

Wt_T_ = Weight of treatment sample (g)

Wt_C_ = Weight of control sample (g)

D_C_ = Dilution factor for control sample

D_T_ = Dilution factor for treatment sample

T = Incubation time (h)

B = Air-dry/oven-dry ratio (If not available, use 1.0.)

Note: µg p-nitrophenol / g oven-dry soil / hour = mg p-nitrophenol / kg oven-dry soil / hour

**Report**

Report acid phosphatase activity as mg p-nitrophenol / kg oven-dry soil / h.

## **Alkaline Phosphatase**

Method code: AlkalinePhosphatase

**Introduction to Assay for Alkaline Phosphatase Enzyme**

Soil enzymes are important to the biochemical functions of organic matter decomposition, and they are catalysts for reactions necessary for the life processes of organic waste, organic matter formation, and nutrient cycling (Das and Varma, 2011). Enzyme assays reflect potential activity. They do not represent true in situ activity levels; therefore, results should be viewed as an index.

**Scope and Field of Application**

Alkaline phosphatase is a common extracellular enzyme produced by many organisms in soil. It removes the phosphate molecule from organic compounds, such as phospholipids and nucleic acids. Once the phosphate is cleaved, it becomes soluble and can be taken up by a cell. This is important because phosphate commonly is the limiting nutrient for microbial growth in soil.

**Principle**

The assay for alkaline phosphatase may be determined on both air-dry and field-moist samples; however, immediate analysis is not required for air-dry samples. Modified Universal Buffer (pH 11) is added to 1-g samples (control and treatment) and then 0.05 M p-nitrophenyl phosphate solution is added to the treatment samples only (Tabatabai and Bremner, 1969). All of the samples are incubated for 1 hour (h) at 37 ºC. After incubation, 0.5 M CaCl_2_ and 0.5 M NaOH are added to each sample. To the control samples only, p-nitrophenyl phosphate solution is added. Samples are allowed to settle for 5 minutes. A 10-mL aliquot is filtered for each sample. A calibration curve is prepared that plots absorbance at 405 nm versus amount of p-nitrophenol. Data are reported as mg p-nitrophenol per kg oven-dry soil per h (Eivazi and Tabatabai, 1977).

**Interferences**

Because p-nitrophenol is light sensitive, samples must be read as soon as possible after filtration.

**Equipment**

- Electronic balance, ±1.0mg sensitivity
  - Volumetric flasks, acid washed, 50 mL, 100 mL, 1000 mL
  - Plastic bottles, amber colored, 1 L
  - Disposable plastic cups, 1 oz. (30 mL)
  - Pipettes, electronic digital, 2500 µL and 10 mL that have 2500-µL and 10-mL tips
  - Pipette, electronic digital, 50 mL
  - Syringe filters, 0.45 µm, Whatman
  - Centrifuge tubes with lids, 50 mL
  - Cuvettes, plastic, 4.5 mL, 1-cm light path
  - Incubator or water bath, 37 ºC
  - Spectrophotometer, UV visible, dual view
  - Vortexer, mini
  - Syringes, 10 mL
  - Beaker, 500 mL
  - pH meter
  - Magnetic stir bars
  - Magnetic stir plate

**Reagents**

- - Reverse osmosis, deionized (RODI) water, ASTM Type I grade reagent water
  - THAM: 2-amino-2(hydroxymethyl)-1-3-propanediol (NH_2_C(CH_2_OH)_3_) (CAS #77-86-1)
  - p-nitrophenyl-phosphate disodium salt hexahydrate (O_2_NC_6_H_4_OP(O)(ONa)_2_ 6H_2_O) (CAS #333338-18-4)
  - p-nitrophenol (C_6_H_5_NO_3_) (CAS #100-02-7)
  - Maleic acid (HO_2_CCH=CHCO_2_H) (CAS #110-16-7)
  - Calcium chloride dihydrate (CaCl_2_•2H_2_O) (CAS #10035-04-8)
  - Citric acid (C_6_H_8_O_7_) (CAS #77-92-9)
  - Boric acid (H_3_BO_3_) (CAS #10043-35-3)
  - Sodium hydroxide (NaOH) (CAS #1310-73-2)
  - Sodium hydroxide solution, 1.0 M

Components.—sodium hydroxide (NaOH), RODI water

- To a 1-L glass volumetric flask, add the following in order:
- 700 mL RODI water,
- 40.00 g NaOH, and
- fill to volume with RODI water.
- Invert to mix.
  - Sodium hydroxide solution, 0.1 M

Components.—1.0 M sodium hydroxide solution, RODI water

- To a 250-mL glass volumetric flask, add the following in order:
- 150 mL RODI water,
- 25 mL 1 M sodium hydroxide,
- fill to volume with RODI water.
- Invert to mix.
  - Sodium hydroxide solution, 0.5 M

Components.—sodium hydroxide (NaOH), RODI water

- To a 1-L glass volumetric flask, add the following in order:
- 700 mL RODI water,
- 20 g NaOH, and
- fill to volume with RODI water.
- Invert to mix.
  - Modified Universal Buffer (MUB) stock solution

Components.—THAM: 2-amino-2 (hydroxymethyl)-1-3-propanediol (NH_2_C(CH_2_OH)_3_), maleic acid (HO_2_CCH=CHCO_2_H), citric acid (C_6_H_8_O_7_), boric acid (H_3_BO_3_), 1.0 M sodium hydroxide solution, RODI water

- To a 1-L glass volumetric flask, add the following in order:
- 488 mL 1 M sodium hydroxide solution,
  - 12.10 g THAM,
- 11.60 g maleic acid,
- 14.00 g citric acid,
  - 6.30 g boric acid, and
- fill to volume with RODI water.
- Store in refrigerator.
  - MUB 11 working solution, pH 11

Components.—MUB stock solution, 0.1 M sodium hydroxide (NaOH) solution, RODI water

- To a 500-mL glass beaker, add a magnetic stir bar. Place beaker on a magnetic stirrer. Add the following in order:
- 200 mL MUB stock solution and
- 1.0 M NaOH solution until the pH reaches 11.0.
- Transfer solution to a 1-L glass volumetric flask, and fill to volume with RODI water.
  - p-nitrophenyl phosphate (pNPP) solution, 0.05 M

Components.—p-nitrophenyl-phosphate disodium salt hexahydrate (O_2_NC_6_H_4_OP(O)(ONa)_2_ 6H_2_O), MUB 11 solution

- To a 50-ml glass volumetric flask, add the following in order:
- 40 mL MUB pH 11 solution,
- 0.9275 g p-nitrophenyl phosphate disodium salt hexahydrate, and
- fill to volume with MUB pH 11 solution.
- Store in a refrigerator.
- Prepare and use for analysis within the same week.
- Reagent sufficient for approximately 25 samples in duplicate (control and treatment).
  - Calcium chloride solution, 0.5 M

Components.—calcium chloride dihydrate (CaCl_2_•2H_2_O), RODI water

- To a 1-L glass volumetric flask, add the following in order:
- 700 mL RODI water,
- 73.5 g CaCl_2_•2H_2_O, and
- fill to volume with RODI water.
- Invert to mix.
  - p-nitrophenol stock standard solution (reagent A)

Components.—p-nitrophenol (C_6_H_5_NO_3_), RODI water

- In a fume hood, add the following in order to a 1-L glass volumetric flask:
- 700 mL RODI water,
- 1.0 g p-nitrophenol, and
- fill to volume with RODI water (after p-nitrophenol is dissolved).
- Store at 4 °C in amber bottle.
- Use within 30 days.
  - p-nitrophenol working standard solution (reagent A1)

Components.—reagent A, RODI water

- In a fume hood, add the following in order to a 100-mL glass volumetric flask:
- 1 mL reagent A and
- fill to volume with RODI water.
- Invert to mix.
- Store at 4 °C in amber bottle.
- Use within 30 days.
  - Standard p-nitrophenol calibration and verification solutions (Standards A2-A6)

Components.—reagent A1, 0.5 M calcium chloride solution, 0.5 M sodium hydroxide solution, RODI water

- Refer to table 1 for mixing instructions.
- Refer to table 2 for concentrations.
- Prepare in 50-mL disposable centrifuge tubes.
- Cap tubes and then shake to mix.

**Table 1: Standards A2-A6—Preparation**

| **Standard p-nitrophenol calibration and verification solutions** | | | | |
| --- | --- | --- | --- | --- |
| **Standard** | **Reagent A1**  (mL) | **0.5 M CaCl_2_** | **0.5 M NaOH** | **RODI**  **water** |
| A6 | 5 | 1 mL | 4 mL | 0 |
| A5 | 4 |  |  | 1 |
| A4 | 3 |  |  | 2 |
| A3 | 2 |  |  | 3 |
| A2 | 1 |  |  | 4 |
| Blank | 0 |  |  | 5 |

**Table 2: Standards A2-A6—Total Amount in 10 ml of Solution**

| **Standard p-nitrophenol**  **calibration and verification solutions** | |
| --- | --- |
| **Standard** | **Amount in 10 ml  of solution** (µg) |
| A6 | 50.0 |
| A5 | 40.0 |
| A4 | 30.0 |
| A3 | 20.0 |
| A2 | 10.0 |
| Blank | 0 |

- - Toluene

Optional application that inhibits microbial growth during long incubation periods. It is not advised to use toluene for a 1-hour incubation period (Dr. Richard Dick, Ohio State University, June 2017).

**Health and Safety**

Wear disposable gloves that have an appropriate polymer rating for chemical resistance, safety glasses and/or face shield, and lab coat or apron when preparing reagents, especially concentrated acids and bases. Dispense concentrated acids and bases in a fume hood. Thoroughly wash hands after handling reagents. Use safety showers and eyewash stations to dilute spilled acids and bases. Use sodium bicarbonate and water to neutralize and dilute spilled acids.

Always work in a fume hood and wear gloves when handling p-nitrophenyl phosphate, p-nitrophenol solutions, and toluene, which is highly flammable. Follow the manufacturer's safety precautions when using the spectrophotometer.

**Preparation of Samples**

Air-dry the field samples at 30 to 35 °C, crush, and sieve to <2 mm. The weight of the air-dry soil samples remains relatively constant. Biological activity of air-dry samples is low during storage.

**Procedure**

- - Weigh 1 g (±0.03 g) of <2-mm, air-dry soil into 50-mL tubes. Label odd-numbered tubes “control” and even-numbered tubes “treatment.”
  - Prior to assay, preheat MUB 11 and substrate to 37 °C. Warm CaCl_2_ and NaOH 0.5M to room temperature.
  - Working in a fume hood, add 4 mL MUB 11 solution to all tubes (control and treatment).
  - Add 1 mL pNPP solution to the treatment, or even-numbered, tubes only. Cap all tubes. Immediately start the 60-minute timer. After starting the timer, use the vortexer to mix all tubes and then place the tubes in a water bath or an incubator.
  - Incubate all samples (control and treatment) at 37 °C for 1 hour. After 1 hour, remove from water bath or incubator.
  - Add 1 mL 0.5 M CaCl_2_ solution and 4 mL 0.5 M NaOH to all tubes. Swirl the tubes. Note: The CaCl_2_ and NaOH reagents may be combined in a ratio of 1:4. Combine reagents 10 to 15 minutes before use. Add 5 mL of the combined reagent to each tube.
  - Add 1 mL pNPP solution to control, or odd-numbered, tubes. Vortex all tubes (control and treatment). Remove caps from tubes. Let samples settle 5 minutes before filtering.
  - Draw up sample into a 10-mL syringe and attach a 0.45-µm filter. Filter sample directly into disposable cup. Transfer filtrate into cuvettes. Because p-nitrophenol is light sensitive, read samples as soon as possible after filtration. Cover the samples waiting to be read.
  - Set the spectrophotometer to read at 405 nm. Auto zero with the calibration blank. Sample concentration is calculated from the regression equation.
  - Record the amount of p-nitrophenol. Read the samples in control/treatment pairs.
  - If readings exceed the 50-µg standard for p-nitrophenol, dilute an aliquot of the filtrate with RODI until the absorbance readings are within the limits of the calibration curve. Dilute an aliquot from both the control and treatment tubes for samples that exceed the standard.

**Calculations**

mg p-nitrophenol / kg oven-dry soil / h = B x [(D_T_ x A_T_ / Wt_T_) − (D_C_ x A_C_ / Wt_C_)] / T

where:

A_T_ = Amount of p-nitrophenol in treatment sample (µg)

A_C_ = Amount of p-nitrophenol in control sample (µg)

Wt_T_ = Weight of treatment sample (g)

Wt_C_ = Weight of control sample (g)

D_C_ = Dilution factor for control sample

D_T_ = Dilution factor for treatment sample

T = Incubation time (h)

B = Air-dry/oven-dry ratio (method 3D1) or field-moist/oven-dry ratio (method 3D2)

Note: µg p-nitrophenol / g oven-dry soil / hour = mg p-nitrophenol / kg oven-dry soil / hour

**Report**

Report alkaline phosphatase activity as mg p-nitrophenol / kg oven-dry soil / h.

## **Arylsulfatase**

Method code: Arylsulfatase

**Introduction to Assay for Arylsulfatase Enzyme**

Soil enzymes are important to the biochemical functions of organic matter decomposition, and they are catalysts for reactions necessary for the life processes of organic waste, organic matter formation, and nutrient cycling (Das and Varma, 2011). Enzyme assays reflect potential activity. They do not represent true in situ activity levels; therefore, results should be viewed as an index.

**Scope and Field of Application**

Arylsulfatase (EC 3.1.6.1) plays an important role in sulfur (S) cycling in soil (Bielka et al., 1984). It can catalyze the hydrolysis of organic sulfate esters, and it can limit the rate of the cycling process (Tabatabai and Bremner, 1970; Speir and Ross, 1978; Chröst, 1991). Arylsulfatase is dominantly in fungi and bacteria, but plants and animals also produce the enzyme (Fitzgerald, 1978).

**Principle**

The arylsulfatase assay can be determined on both air-dry and field-moist samples; however, immediate analysis is not required for air-dry samples. Acetate buffer 0.5 M pH 5.8 is added to 1-g samples (control and treatment) and then 0.05 M potassium
4-nitrophenyl sulfate is added to the treatment samples. All of the samples are incubated for 1 hour at 37 °C. After incubation, 0.5 M calcium chloride and 0.5 M sodium hydroxide are added to all the samples. To the control samples only, 0.05 M potassium 4-nitrophenyl sulfate is added. Samples are allowed to settle for 5 minutes. A 10-mL aliquot is filtered for each sample. A calibration curve is prepared that plots absorbance at 405 nm versus amount of p-nitrophenol. Data are reported as mg p-nitrophenol per kg oven-dry soil per h.

**Interferences**

Because p-nitrophenol is light sensitive, samples must be read as soon as possible after filtration.

**Equipment**

- Electronic balance, ±1.0-mg sensitivity
  - Volumetric flasks, acid washed, 50 mL, 100 mL, 1000 mL
  - Plastic bottles, amber colored, 1000 mL
  - Disposable plastic cups, 1 oz. (30 mL)
  - Pipettes, electronic digital, 2500 µL and 10 mL, 2500-µL and 10-mL tips
  - Pipette, electronic digital, 50 mL
  - Syringe filters, 0.45 µm, Whatman
  - Centrifuge tubes with lids, 50 mL
  - Cuvettes, plastic, 4.5 mL, 1-cm light path
  - Incubator or water bath, 37 °C
  - Spectrophotometer, UV visible, dual view
  - Vortexer, mini
  - Syringes, 10 mL
  - Beaker, 500 mL
  - pH meter
  - Magnetic stir bars
  - Magnetic stir plate

**Reagents**

- - Reverse osmosis, deionized (RODI) water, ASTM Type I grade reagent water
  - Potassium 4-nitrophenyl sulfate (NO_2_C_6_H_4_OSO_2_OK) (CAS #6217-68-1)
  - Sodium acetate trihydrate (C_2_H_3_NaO_2_•3H_2_O) (CAS #6131-90-4)
  - Glacial acetic acid (99%) (CH_3_CO_2_H) (CAS #64-19-7)
  - Calcium chloride dihydrate (CaCl_2_•2H_2_O) (CAS #10035-04-8)
  - Sodium hydroxide (NaOH) (CAS #1310-73-2)
  - p-nitrophenol (C_6_H_5_NO_3_) (CAS #100-02-7)
  - Acetate buffer solution, 0.5 M, pH 5.8

Components.—sodium acetate trihydrate, (C_2_H_3_NaO_2_•3H_2_O), glacial acetic acid (CH_3_CO_2_H), RODI water

- To a 1-L glass volumetric flask, add the following in order:
- 700 ml RODI water,
- 68 g sodium acetate trihydrate,
- 1.70 mL glacial acetic acid (to adjust the pH to 5.8), and
- fill to total volume with RODI water.
  - Potassium 4-nitrophenyl sulfate, 0.05 M

Components.—potassium 4-nitrophenyl sulfate (NO_2_C_6_H_4_OSO_2_OK), 0.5 M acetate buffer solution

- To a 50-mL glass volumetric flask, add the following in order:
- 40 mL acetate buffer solution,
- 0.614 g potassium 4-nitrophenyl sulfate, and
- fill to volume with acetate buffer solution.
- Store in a refrigerator.
- Prepare and use for analysis within the same week.
- Reagent sufficient for approximately 25 samples in duplicate (control and treatment).
  - Calcium chloride solution, 0.5 M

Components.—calcium chloride dihydrate (CaCl_2_•2H_2_O), RODI water

- To 1-L glass volumetric flask, add the following in order:
- 700 mL RODI water,
- 73.5 g CaCl_2_•2H_2_O, and
- fill to volume with RODI water.
- Invert to mix.
  - Sodium hydroxide solution, 0.5 M

Components.—sodium hydroxide (NaOH), RODI water

- To a 1-L glass volumetric flask, add the following in order:
- 700 mL RODI water,
- 20 g NaOH, and
- fill to volume with RODI water.
- Invert to mix.
  - p-nitrophenol stock standard solution (reagent A)

Components.—p-nitrophenol (C_6_H_5_NO_3_), RODI water

- In a fume hood, add the following in order to a 1-L glass volumetric flask:
- 700 mL RODI water,
- 1.0 g p-nitrophenol, and
- fill to volume with RODI water (after p-nitrophenol is dissolved).
- Invert to mix.
- Store at 4 °C in amber bottle.
- Use within 30 days.
  - p-nitrophenol working standard solution (reagent A1)

Components.—reagent A, RODI water

- In a fume hood, add the following in order to a 100 mL-volumetric flask:
- 1 mL reagent A and
- fill to volume with RODI water.
- Invert to mix.
- Store at 4 °C in amber bottle.
- Use within 30 days.
  - Standard p-nitrophenol calibration and verification solutions (Standards A2-A6)

Components.—reagent A1, 0.5 M calcium chloride solution, 0.5 M sodium hydroxide solution, RODI water

- Prepare in 50-mL centrifuge tubes.
- Refer to table 1 for mixing instructions.
- Refer to table 2 for concentrations.
- Bring to volume with RODI water.
- Cap tubes and shake to mix.

**Table 1:** **Standards A2-A6—Preparation**

| **Standard p-nitrophenol calibration and verification solutions** | | | | | |
| --- | --- | --- | --- | --- | --- |
| **Standard** | **Final amount**  (µg) | **Reagent A1**  (mL) | **0.5 M CaCl_2_** | **0.5 M NaOH** | **RODI**  **Water**  (mL) |
| A6 | 50.0 | 5 | 1 mL | 4 mL | 0 |
| A5 | 40.0 | 4 |  |  | 1 |
| A4 | 30.0 | 3 |  |  | 2 |
| A3 | 20.0 | 2 |  |  | 3 |
| A2 | 10.0 | 1 |  |  | 4 |
| Blank | 0 | 0 |  |  | 5 |

**Table 2:** **Standards A2-A6—Total Amount in 10 ml of Solution**

| **Standard p-nitrophenol calibration and  verification solutions** | |
| --- | --- |
| **Standard** | **Amount in 10 ml** **of solution** (µg) |
| A6 | 50.0 |
| A5 | 40.0 |
| A4 | 30.0 |
| A3 | 20.0 |
| A2 | 10.0 |
| Blank | 0 |

- - Toluene

Optional application that inhibits microbial growth during long incubation periods. It is not advised to use toluene for a 1-hour incubation period (Dr. Richard Dick, Ohio State University, June 2017).

**Health and Safety**

Wear disposable gloves that have an appropriate polymer rating for chemical resistance, safety glasses and/or face shield, and a lab coat or apron when preparing reagents, especially concentrated acids and bases. Thoroughly wash hands after handling reagents. Use safety showers and eyewash stations to dilute spilled acids and bases. Use sodium bicarbonate and water to neutralize and dilute spilled acids.

Always work in a fume hood and wear gloves when weighing and handling
p-nitrophenol and potassium 4-nitrophenyl sulfate solutions. Follow the manufacturer's safety precautions when using the spectrophotometer.

**Preparation of Samples**

Air-dry the field sample at 30 to 35 °C, crush, and sieve to <2 mm. The weight of the air-dried soil sample remains relatively constant. Biological activity of the air-dried sample is low during storage.

**Procedure**

- - Weigh 1 g (±0.03 g) of <2-mm, air-dry soil into 50-ml tubes. Label odd-numbered tubes “control” and even-numbered tubes “treatment.”
  - Prior to assay, preheat acetate buffer and substrate to 37 °C. Warm CaCl_2_ and NaOH 0.5 M to room temperature.
  - Working in a fume hood, add 4 mL acetate buffer solution to all tubes (control and treatment).
  - Add 1 mL potassium 4-nitrophenyl sulfate solution to the treatment tubes only. Cap all tubes. Immediately start the 60-minute timer. After starting the timer, use vortexer to mix tubes and place tubes in a water bath or an incubator.
  - Incubate or water bathe all samples at 37 °C for 1 hour. After 1 hour, remove from incubator or water bath.
  - Add 1 mL 0.5 M calcium chloride solution and 4 mL 0.5 M sodium hydroxide solution to all tubes. Swirl the tubes.

Note: The CaCl_2_ and NaOH reagents may be combined in a ratio of 1:4. Combine reagents 10 to 15 minutes before use. Add 5 mL of the combined reagent to each tube.

- - Add 1 mL potassium 4-nitrophenyl sulfate solution to control tubes. Use vortexer to mix the tubes. Remove caps. Let samples settle 5 minutes before filtering.
  - Draw up a sample into a 10 mL syringe and attach a 0.45-µm filter. Filter sample directly into disposable cup. Transfer filtrates into cuvettes. Because p-nitrophenol is light sensitive, read samples as soon as possible after filtration. Cover the samples waiting to be read.
  - Set the spectrophotometer to read at 405 nm. Auto zero with the calibration blank. Sample concentration is calculated from the regression equation.
  - Record the amount of p-nitrophenol. Read the samples in control/treatment pairs.
  - If the color intensity of the original filtrate exceeds the 50-µg high standard, dilute an aliquot of the filtrate with RODI until the colorimeter reading is within the limits of the calibration graph. Dilute an aliquot from both the control and treatment tubes for samples that exceed the standard.

**Calculations**

mg p-nitrophenol / kg oven-dry soil / h = B x [(D_T_ x A_T_ / Wt_T_) − (D_C_ x A_C_ / Wt_C_)] / T

where:

A_T_ = Amount of p-nitrophenol in treatment sample (µg)

A_C_ = Amount of p-nitrophenol in control sample (µg)

Wt_T_ = Weight of treatment sample (g)

Wt_C_ = Weight of control sample (g)

D_C_ = Dilution factor for control sample

D_T_ = Dilution factor for treatment sample

T = Incubation time (h)

B = Air-dry/oven-dry ratio (method 3D1) or field-moist/oven-dry ratio (method 3D2)

Note: µg p-nitrophenol / g oven-dry soil / hour = mg p-nitrophenol / kg oven-dry soil / hour

**Report**

Report arylsulfatase activity as mg p-nitrophenol / kg oven-dry soil / h.

## **N-acetyl-B-glucoaminidase**

Method code: Bglucosaminidase

**Introduction to Assay for PNAG Enzyme**

Soil enzymes are important to the biochemical functions of organic matter decomposition, and they are catalysts for reactions necessary for the life processes of organic waste, organic matter formation, and nutrient cycling (Das and Varma, 2011). Enzyme assays reflect potential activity. They do not represent true in situ activity levels; therefore, results should be viewed as an index.

**Scope and Field of Application**

N-acetyl-β-D-glucosaminidase (NAGase, EC 3.2.1.52) (Bielka et al., 1984) is one of three enzymes that catalyze the hydrolysis of chitin (Tronsmo and Harman, 1993). This hydrolysis is important in the cycling of carbon (C) and nitrogen (N) in soils because it participates in the processes that convert chitin to amino sugars, which are a major source of mineralizable N in soils (Wood et al., 1994; Sinsabaugh and Moorhead, 1995). N-acetyl-β-glucoaminidase also is involved in biological control of plant pathogens (Chet and Baker, 1981). This enzyme is highly correlated to fungal biomass (Miller et al., 1998).

**Principle**

The assay for N-acetyl-ß-glucoaminidase may be determined on both air-dry and field-moist samples; however, immediate analysis is not required for air-dry samples. Acetate buffer (100 mM, pH 5.5) is added to 1-g samples (control and treatment) and then 10 mM p-nitrophenyl-N-acetyl-β-glucosaminidase (PNAG) is added to the treatment samples only. All of the samples are incubated for 1 h at 37 °C. After incubation, 0.5 M CaCl_2_ and 0.1 M THAM pH 12 are added to each sample. To the control sample only, PNAG is added. All samples are allowed to settle for 5 minutes. A 10-mL aliquot of each sample is filtered. A calibration curve is prepared that plots the absorbance at 405 nm versus the amount of p-nitrophenol. If readings exceed the high standard, the sample is diluted with a 1:1 mixture of acetate buffer pH 5.5 and 0.1 M THAM pH 12. Data are reported as mg p-nitrophenol per kg oven-dry soil per h.

**Interferences**

Because p-nitrophenol is light sensitive, samples must be read as soon as possible after filtration.

**Equipment**

- - Electronic balance, ±1.0 mg sensitivity
  - Volumetric flasks, acid washed, 50 mL, 100 mL, 1000 mL
  - Plastic bottles, amber colored, 1000 mL
  - Disposable plastic cups, 1 oz. (30 mL)
  - Pipettes, electronic digital, 2500-uL and 10 mL, 2500-uL and 10-mL tips
  - Pipette, electronic digital, 50 mL
  - Syringe filters, 0.4 µm, Whatman
  - Centrifuge tubes with lids, 50 mL
  - Cuvettes, plastic, 4.5 mL, 1-cm light path
  - Incubator or water bath, 37 °C
  - Spectrophotometer, UV visible
  - Vortexer, mini
  - Syringes, 10 mL
  - Beaker, 500 mL
  - pH meter
  - Magnetic stir bars
  - Magnetic stir plate

**Reagents**

- - Reverse osmosis, deionized (RODI) water, ASTM Type I grade reagent water
  - THAM: tris(hydroxymethyl)aminomethane (NH_2_C(CH_2_OH)_3_) (CAS #77-86-1)
  - 4-nitrophenyl N-acetyl-β-D-glucosaminide (C_14_H_18_N_2_O_8_) (CAS #3459-18-5)
  - Calcium chloride dihydrate (CaCl_2_•2H_2_O) (CAS #10035-04-8)
  - Sodium acetate trihydrate (C_2_H_3_NaO•3H_2_O) (CAS #6131-90-4)
  - Glacial acetic acid (99%) (CH_3_CO_2_H) (CAS #64-19-7)
  - p-nitrophenol (C_6_H_5_NO_3_) (CAS #100-02-7)
  - Sodium hydroxide (NaOH) (CAS #1310-73-2)
  - Acetate buffer solution, 100 mM, pH 5.5

Components.—sodium acetate trihydrate, (C_2_H_3_NaO•3H_2_O), glacial acetic acid (99%) (CH_3_CO_2_H), RODI water

- To a 1-L glass volumetric flask, add the following in order:
- 800 mL RODI water,
- 13.6 g sodium acetate trihydrate,
- adjust to pH 5.5 with glacial acetic acid, and
- fill to volume with RODI water.
- Store solution in a refrigerator.
  - PNAG solution, 10 mM

Components.—acetate buffer solution, 4-nitrophenyl N-acetyl-β-D-glucosaminide (C_14_H_18_N_2_O_8_)

- To a 100-mL volumetric flask, add the following in order:
- 80 mL acetate buffer solution,
- 0.342 g 4-nitrophenyl N-acetyl-β-D-glucosaminide, and
- fill to volume with acetate buffer solution.
- Store solution in a refrigerator.
- Prepare and use for analysis within the same week.
- Reagent sufficient for approximately 50 samples in duplicate (control and treatment samples.)
  - Calcium chloride solution, 0.5 M

Components.—calcium chloride dihydrate (CaCl_2_•2H_2_O), RODI water

- To a 1-L glass volumetric flask, add the following in order:
- 700 mL RODI water,
- 73.5 g CaCl_2_•2H_2_O, and
- fill to volume with RODI water.
- Invert to mix.
  - Sodium hydroxide solution, 0.5 M

Components.—sodium hydroxide (NaOH), RODI water

- To a 1-L glass volumetric flask, add the following in order:
- 700 mL RODI water,
- 20 g NaOH, and
- fill to volume with RODI water.
- Invert to mix.
  - THAM 12 solution, 0.1 M, pH 12

Components.—THAM: tris(hydroxymethyl)aminomethane (NH_2_C(CH_2_OH)_3_), sodium hydroxide solution, RODI water

- To a 1-L volumetric flask, add the following in order:
- 800 mL RODI water,
- 12.20 g THAM,
- 0.5 M NaOH to adjust the pH to 12, stirring constantly, and
- fill to volume with RODI water.
  - p-nitrophenol stock standard solution (reagent A)

Components.—p-nitrophenol (C_6_H_5_NO_3_), RODI water

- In a fume hood, add the following in order to a 1-L volumetric flask:
- 700 mL RODI water,
- 1.0 g p-nitrophenol, and
- fill to volume with RODI water (after p-nitrophenol is dissolved).
- Store in amber bottle at 4 °C.
- Use within 30 days.
  - p-nitrophenol working standard solution (reagent A1)

Components.—reagent A, RODI water

- In a fume hood, add the following in order to a 100-mL volumetric flask:
- 1 mL reagent A and
- fill to volume with RODI water.
- Invert to mix.
- Store in amber bottle at 4 °C.
- Prepare and use for analysis within the same week.
  - Standard p-nitrophenol calibration and verification solutions (reagents A2-A6)

Components.—reagent A1, THAM 12 solution, calcium chloride solution, RODI water

- Refer to table 1 for mixing instructions.
- Refer to table 2 for concentrations.
- Standards are not incubated.
- Prepare in 50-mL centrifuge tubes. Cap tubes and shake to mix.

**Table 1: Standards A2-A6—Preparation**

| **Standard p-nitrophenol calibration and verification solutions** | | | | | |
| --- | --- | --- | --- | --- | --- |
| **Standard** | **Final amount** (µg) | **Reagent A1**  (mL) | **CaCl_2_** | **THAM 12** | **RODI**  **water** |
| A6 | 50.0 | 5 | 1 mL | 4 mL | 0 |
| A5 | 40.0 | 4 |  |  | 1 |
| A4 | 30.0 | 3 |  |  | 2 |
| A3 | 20.0 | 2 |  |  | 3 |
| A2 | 10.0 | 1 |  |  | 4 |
| Blank | 0 | 0 |  |  | 5 |

**Table 2:** **Reagents A2-A6—Total Amount in 10 ml of Solution**

| **Standard p-nitrophenol calibration and verification concentrations** | |
| --- | --- |
| **Standard** | **Amount** **in 10 ml of solution** (µg) |
| A6 | 50.0 |
| A5 | 40.0 |
| A4 | 30.0 |
| A3 | 20.0 |
| A2 | 10.0 |
| Blank | 0 |

- - Toluene

Optional application that inhibits microbial growth during long incubation periods. It is not advised to use toluene for a 1-hour incubation period (Dr. Richard Dick, Ohio State University, June 2017).

**Health and Safety**

Wear disposable gloves that have an appropriate polymer rating for chemical resistance, safety glasses and/or face shield, and a lab coat or apron when preparing reagents, especially concentrated acids and bases. Dispense concentrated acids and bases in a fume hood. Thoroughly wash hands after handling reagents. Use safety showers and eyewash stations to dilute spilled acids and bases. Use sodium bicarbonate and water to neutralize and dilute spilled acids.

Always work in a fume hood and wear gloves when weighing and handling p-nitrophenol and PNAG solutions.

Follow the manufacturer's safety precautions when using the spectrophotometer.

**Preparation of Samples**

Air-dry the field sample at 30 to 35 °C, crush, and sieve to <2 mm. The weight of the air-dried soil sample remains relatively constant. Biological activity of the air-dried sample is low during storage.

**Procedure**

- - Weigh 1 g (±0.03 g) of <2-mm, air-dry soil into centrifuge tubes. Label odd-numbered tubes “control” and even-numbered tubes “treatment.”
  - Prior to assay, preheat acetate buffer and substrate to 37 °C. Warm CaCl_2_ and NaOH 0.5M to room temperature.
  - Working in a fume hood, add 4 mL acetate buffer solution to all tubes (control and treatment).
  - Add 1 mL PNAG solution to the treatment tubes only. Cap all tubes. Immediately start the 60-minute timer. After starting the timer, use vortexer to mix the tubes. Place the tubes in a water bath or an incubator.
  - Incubate all samples (control and treatment) at 37 °C for 1 hour. After 1 hour, remove from the water bath or incubator.
  - Add 1 mL 0.5 M calcium chloride solution and 4 mL THAM 12 solution to all tubes. Swirl the tubes.

Note: The CaCl_2_ and THAM reagents may be combined in a ratio of 1:4. Combine reagents 10 to 15 minutes before use. Add 5 mL of the combined reagent to each tube.

- - Add 1 mL PNAG solution to control tubes only. Cap all tubes. Mix all tubes in vortexer. Remove caps. Let samples settle 5 minutes before filtering.
  - Draw up sample into a 10-mL syringe and attach a 0.45-µm filter. Filter sample directly into disposable cup. Transfer to cuvettes. Because p-nitrophenol is light sensitive, read samples as soon as possible after filtration. Cover the samples waiting to be read.
  - Prepare calibration curve to plot absorbance at 405 nm versus amount of p-nitrophenol. Auto zero with the calibration blank.
  - Read the samples in control/treatment pairs. Record amounts of p-nitrophenol.
  - If readings exceed the high standard, dilute original extract with a 1:1 mixture of the acetate buffer pH 5.5 and 0.1 M THAM pH 12 until the absorbance readings are within the limits of the calibration curve. Dilute the original extract of both the treatment and control samples that exceed the standard.

**Calculations**

mg p-nitrophenol / kg oven-dry soil / h = B x [(DT x AT / WtT) − (DC x AC / WtC)] / T

where:

AT = Amount of p-nitrophenol in treatment sample (µg)

AC = Amount of p-nitrophenol in control sample (µg)

WtT = Weight of treatment sample (g)

WtC = Weight of control sample (g)

DC = Dilution factor for control sample

DT = Dilution factor for treatment sample

T = Incubation time (h)

B = Air-dry/oven-dry ratio (method 3D1) or field-moist/oven-dry ratio (method 3D2)

Note: µg p-nitrophenol / g oven-dry soil / hour = mg p-nitrophenol / kg oven-dry soil / h

**Report**

Report N-acetyl-B-glucosaminidase activity as mg p-nitrophenol / kg oven-dry soil / hour.

**References**

Acosta-Martínez, V., & Tabatabai, M. A. (2011). Phosphorus Cycle Enzymes. In Methods of Soil Enzymology (pp. 161–183). John Wiley & Sons, Ltd. https://doi.org/10.2136/sssabookser9.c8

Acosta-Martínez, V., & Tabatabai, M. A. (2000). Enzyme activities in a limed agricultural soil. Biology and Fertility of Soils, 31(1), 85–91. https://doi.org/10.1007/s003740050628

Bandick, A. K., & Dick, R. P. (1999). Field management effects on soil enzyme activities. Soil Biology and Biochemistry, 31(11), 1471–1479. https://doi.org/10.1016/S0038-0717(99)00051-6

Chróst, R. J. (1990). Microbial Ectoenzymes in Aquatic Environments. In J. Overbeck & R. J. Chróst (Eds.), Aquatic Microbial Ecology: Biochemical and Molecular Approaches (pp. 47–78). Springer. https://doi.org/10.1007/978-1-4612-3382-4_3

Deng, S., & Popova, I. (2011). Carbohydrate Hydrolases. In Methods of Soil Enzymology (pp. 185–209). John Wiley & Sons, Ltd. https://doi.org/10.2136/sssabookser9.c9

Eivazi, F., & Tabatabai, M. A. (1977). Phosphatases in soils. Soil Biology and Biochemistry, 9(3), 167–172. https://doi.org/10.1016/0038-0717(77)90070-0

Eivazi, F., & Tabatabai, M. A. (1988). Glucosidases and galactosidases in soils. Soil Biology and Biochemistry, 20(5), 601–606. https://doi.org/10.1016/0038-0717(88)90141-1

Klose, S., Moore, J. M., & Tabatabai, M. A. (1999). Arylsulfatase activity of microbial biomass in soils as affected by cropping systems. Biology and Fertility of Soils, 29(1), 46–54. https://doi.org/10.1007/s003740050523

Madejón, E., Burgos, P., López, R., & Cabrera, F. (2001). Soil enzymatic response to addition of heavy metals with organic residues. Biology and Fertility of Soils, 34(3), 144–150. https://doi.org/10.1007/s003740100379

Parham, J. A., & Deng, S. P. (2000). Detection, quantification and characterization of β-glucosaminidase activity in soil. Soil Biology and Biochemistry, 32(8), 1183–1190. https://doi.org/10.1016/S0038-0717(00)00034-1

Tabatabai, M. A., & Bremner, J. M. (1970). Arylsulfatase Activity of Soils. Soil Science Society of America Journal, 34(2), 225–229. https://doi.org/10.2136/sssaj1970.03615995003400020016x

Tronsmo, A., & Harman, G. E. (1993). Detection and quantification of N-acetyl-beta-D-glucosaminidase, chitobiosidase, and endochitinase in solutions and on gels. Analytical Biochemistry, 208(1), 74–79. https://doi.org/10.1006/abio.1993.1010

# **Laboratory Method 6: Permanganate Oxidizable C (Active Carbon)**

**Standard Procedure**

Method code: POX_C

**Application**

This method is commonly called the POXC or Weil active carbon method (Weil et al., 2003), but it is referred to as the reactive carbon method by the KSSL. It is a quick, easy field test for the assessment of reactive soil organic carbon (C). Following the principle of bleaching chemistry, potassium permanganate (KMnO4) is used to oxidize organic matter in soil. The oxidized organic matter is associated with the reactive C pool (Blair et al., 1995). A reactive soil organic C index can be expressed as the quotient of reactive soil organic C to soil organic C (Blair et al., 2001). The stability of this index over time is a useful measure of soil quality (Islam and Weil, 1997). POXC measures the portion of the soil organic matter that is readily available as a source of food and energy for the soil microbial community, which helps to maintain a healthy web of food in soil.

**Summary of Method**

To measure POXC, soil is reacted with a potassium permanganate solution, which has a deep purple color. As the solution oxidizes, it loses some of its color. This loss of color upon reaction is directly proportional to the amount of POXC in a soil sample; it is determined by using a spectrophotometer and calibrated against standards of known concentration. Soil samples are air-dried to a constant weight, shaken with 0.02 KMnO_4_ solution, allowed to settle or centrifuged, and diluted, and then absorbance is measured at 550 nm. This standard operating procedure is from Moebius-Clune (2016) and is based on the method of Weil et al. (2003). Reactive carbon is reported as milligrams POXC per kilogram oven-dry soil (mg reactive carbon kg−1).

**Interferences**

Chemical oxidation methods for the determination of labile soil C have a number of limitations. Soil samples may have variable amounts of readily oxidizable fractions, which makes standardization of any method difficult. Results are influenced by the amount of C in a sample, the concentration of MnO4, and the contact time (Blair et al., 1995).

**Safety**

Wear protective clothing (coat, apron, and gloves) and eye protection (safety glasses and other equipment, as appropriate) while preparing reagents and performing procedure. Exercise special care when preparing reagents. Use a vented hood. Thoroughly wash hands after handling all chemicals.

Potassium permanganate is a strong oxidizer. Avoid contact with eyes, skin, and clothing. Contact with other material may cause a fire. In case of fire, soak with water. In case of spill, sweep up, remove, and flush spill area with water.

Inhalation of KMnO4 dust may severely damage respiratory passages and lungs. Contact with skin or eyes may cause severe irritation or burns. Substance is readily absorbed through the skin. See the Material Safety Data Sheet (MSDS) for further information regarding KMnO4.

**Collecting, Handling, and Processing of Soil Samples**

Ensure the soil sample collection bags are properly labeled. Keep collected samples cool and out of sunlight while in the field (preferably in a cooler).

After returning from the field, place samples in a cool area (refrigerator).

Process samples by air-drying to a constant weight at 30 – 35 ^o^C in a forced air-drying oven and sieving through a 2-mm screen.

**Equipment**

- - Centrifuge tubes, 50 mL, graduated, polyethylene, screw tops
  - Dropper pipette, 1 mL, graduated
  - Squirt bottle
  - Electronic balance, 5 g (or electronic balance, ±0.01-g sensitivity)
  - Petri dish for sun-drying crumbled soil, if necessary
  - Stopwatch, timer, or watch that has a second hand
  - Pipette, electronic digital, 10 mL
  - Volumetric flasks, 50 mL, 100 mL, 250 mL, and 2 L that have stoppers
  - Spectrophotometer, UV visible; Varian, Cary 50 Conc, Varian Australia Pty Ltd., or equivalent
  - Computer that has Cary WinUV software, Varian Australia Pty Ltd., and printer
  - Cuvettes, plastic, 4.5 mL, 1-cm light path, Daigger Scientific
  - Vortexer

**Reagents**

- - Reverse osmosis, deionized (RODI) water, ASTM Type I grade reagent water
  - CaCl2•2H2O, 0.1 M solution. In a 2-L volumetric flask, dissolve 29.40 g CaCl2•2H2O in 1L RODI water. Bring to a volume of 2 L. Store in a polyethylene bottle.
  - KOH, 0.1 M solution. In a 100-mL volumetric flask, dissolve 0.561 g potassium hydroxide in 50 mL RODI water. Bring to volume with RODI water. Invert to mix thoroughly.
  - Stock KMnO4 solution, 0.2 M in 0.1 M CaCl2 solution (pH 7.2). In a 250-mL volumetric flask, dissolve 7.90 g potassium permanganate crystals in 100 mL 0.1 M CaCl2. Bring to volume with 0.1 M CaCl2. Adjust pH of solution to 7.2 with 0.1 M KOH, generally 1 or 2 drops of KOH. May take several hours to dissolve; cover flask with aluminum foil. Solution is stable for 3 days.
  - Working KMnO4, 0.02 M. Add 200 mL KMnO4 stock solution in a 2-L volumetric flask. Bring to volume with RODI water and invert. Store in the dark in the refrigerator. Solution is stable for 3 days.
  - Standard KMnO4 working solutions (SKMnO4WS), 0.04, 0.02, 0.01, and 0.005 M KMnO4. To four 50-mL volumetric flasks, add 10, 5, 2.5, and 1.25 mL of reagent 6.4 (0.2 M KMnO4, in 0.1 M CaCl2 solution, pH 7.2), respectively. Bring to volume with RODI water. Invert to mix thoroughly. Store in a refrigerator. Allow to equilibrate to room temperature before use. Prepare fresh weekly.
  - Standard KMnO4 calibration solutions (SKMnO4CS), 0.0004, 0.0002, 0.0001, 0.00005, and 0 M KMnO4. To four 50-mL volumetric flasks, add 0.5-mL aliquots of the SKMnO4WS. For the blank, add 5 mL 0.1 M CaCl2. Bring to volume with RODI water. Invert to mix thoroughly. Make fresh daily.

**Procedure**

- - Weigh 2.5 g of <2-mm sieved, air-dry soil to the nearest mg. Add to centrifuge tube. Add 20 mL working KMnO_4_, 0.2 M.
  - Vortex each sample. Allow sample to settle for 10 minutes. Do not disturb during settling period.
  - After 10 minutes, centrifuge 10 minutes at 2000 rpm.
  - Add 49.5 mL RODI water to a clean, labeled centrifuge tube. Transfer 0.5 mL of the supernatant solution to the tube and mix.
  - Transfer sample extracts and SKMnO_4_CS to cuvettes.
  - Set the spectrophotometer to 550 nm. Auto zero with calibration blank.
  - Use the calibration solutions to calibrate the instrument. The data system then associates the concentrations with the instrument responses for each calibration solution. Rejection criteria for calibration is R_2_ <0.99.
  - Run samples using calibration curve. Sample concentration is calculated from the regression equation. Record results to the nearest 0.01 unit for the sample extract and each calibration solution.
  - If samples have <0.00003 absorbance (A), reweigh a smaller sample size (e.g., 2.50 g) and re-analyze. Samples that have low absorbance have a large amount of reactive carbon.

**Calculations**

The bleaching (loss of purple color; reduction in absorbance) of the KMnO4 is proportional to the amount of oxidizable C in a soil sample. It is assumed that 1 mole (mol) MnO4 is consumed (reduced from Mn7+ to Mn^2+^) in the oxidation of 0.75 mol (9000 mg) C.

KMnO4 C (mg kg−1) = (0.02 mol L−1 − A) x (9000 mg C mol−1) x (0.02 L solution /

0.005 kg) x AD/OD

where:

0.02 mol L−1 = Initial solution concentration

A = Analyte reading (mol L−1)

9000 = Mg C (0.75 mole) oxidized by 1 mole MnO4, changing from Mn7+ to Mn^2+^

0.02 L = Volume of KMnO_4_ solution reacted

0.005 = Kg of soil used

AD/OD = Air-dry/oven-dry ratio (If not available, use 1.00.)

**Report**

Report reactive C (mg kg−1) as oxidizable C, potassium permanganate (POXC).

**Alternate Procedure (Stock solution, Shaker, and Settling)**

Moebius-Clune, B.N., D.J. Moebius-Clune, B.K. Gugino, O.J. Idowu, R.R. Schindelbeck, A.J. Ristow, H.M. van Es, J.E. Thies, H. A. Shayler, M. B. McBride, D.W. Wolfe, and G.S. Abawi, 2016. Comprehensive Assessment of Soil Health – The Cornell Framework Manual, Edition 3.0, Cornell University, Geneva, NY. Available at [CASH-Standard-Operating-Procedures-030217final-u8hmwf.pdf (bpb-us-e1.wpmucdn.com)](https://bpb-us-e1.wpmucdn.com/blogs.cornell.edu/dist/7/9922/files/2021/11/CASH-Standard-Operating-Procedures-030217final-u8hmwf.pdf)

Method code: POX_C002

**Preparation of 0.2 M KMnO4 Stock Solution (1 liter)**

- In a beaker, dissolve 11.09 g CaCl2 in ~750 mL dH2O. Dissolve completely using a stir plate with a stir bar (final concentration 0.1 M).
- Add 31.61 g KMnO4 to the solution and an additional 200 mL dH2O. Cover solution and stir plate with an opaque box or paper bag and allow to dissolve completely.
- Ensure the pH meter is properly calibrated.
- Measure pH of solution. Final pH should be 7.2.
- Depending on pH measurement, make a dilute (~0.1 M) acid or base solution using HCl or KOH. Using a pipettor, slowly add acid or base and monitor pH until it is at a constant of 7.2.
- Pour solution into a 1-L volumetric flask and bring to 1000 mL with dH2O. Because the solution is light sensitive, transfer it to an opaque bottle. Label and date the bottle. The solution remains stable 3 to 6 months.

**Equipment**

- 50-mL centrifuge tubes with caps (e.g., Falcon® tubes), racks
- Bottle-top solution dispenser
- pH meter and buffered calibration solutions
- Analytical balance (with 3 decimal places)
- Colorimeter (with 550-nm setting)
- Kimwipes® or other laboratory tissue
- 100- to 1000-µl pipettor and disposable tips
- Platform shaker
- Stopwatch
- Stir plate and stir bar
- 1000-mL volumetric flasks, beakers, and graduated cylinder
- Amber bottles

**Measuring Active Carbon in Soil Samples**

- Sieve soil to 2 mm and air-dry to constant mass.
- Run each soil sample in duplicate. This requires two centrifuge tubes with 18 mL dH2O and two centrifuge tubes with 18.8 mL dH2O.
- Generally, run samples in groups of 20 per rack (10 duplicate soil samples).
- Dispense distilled water into a beaker for as many as ten soil samples. Set aside.
- In centrifuge tubes, measure two 2.5 g replicates for each soil sample (±0.005 g).
- Dispense small amounts of 0.2 M KMnO4 solution into beaker as needed. Cover with an opaque container to block from light.
- In sequence, add 18 mL dH2O to each tube containing a soil sample. In same sequence, then begin redox reaction by adding 2 mL 0.2 M KMnO4 to each tube. Cap tightly.
- Place tubes and rack on the shaker at 120 rpm. Start stopwatch. Allow to shake 2 minutes.
- After 2 minutes (do not stop stopwatch), remove samples from the shaker and “slosh” solution in tubes to ensure soil is not stuck to the cap or top of the tube. Uncap tubes. On benchtop, allow settling and reaction to continue for another 8 minutes.
- After the total reaction time of 10 minutes, remove 0.2 mL from each reaction tube and transfer to a centrifuge tube with 18.8 mL distilled water. Dispensing this 0.2 mL aliquot from the reaction tube into 18.8 mL distilled water is a dilution of 100 times, which ends the reaction.
- After all reactions have been ended, cap the diluted sample tubes and shake them by hand 10 seconds.
- Immediately read and record absorbance of each sample.
- Rerun duplicates that have a difference in absorbance of more than 5 percent.
- Clean all materials using dH2O, particularly colorimeter cuvettes if they will be reused.

**Standard Curve**

- Ensure the colorimeter is set to 550 nm and zero with dH2O.
- Dispense 45 mL dH2O into each of three centrifuge tubes.
- Add additional dH2O to the tubes in the following volumes:
- tube 1, 3.75 mL;
- tube 2, 2.50 mL; and
- tube 3, 0.00 mL.
- Add 0.2 M KMnO4 to the tubes in the following volumes:
- tube 1, 1.25 mL;
- tube 2, 2.50 mL; and
- tube 3, 5.00 mL.
- Final concentrations of the 50-mL KMnO4 standard solutions are now 0.005 M, 0.01 M, and 0.02 M. Cap tubes. Shake tubes for 10 seconds.
- Dispense 20 mL distilled water into nine Falcon® tubes—three for each standard solution.
- Add 0.2 mL of each standard solution to each triplicate set. Cap tubes. Shake tubes for 10 seconds.
- Fill a cuvette with one volume of standard solution and then clean the outside with a Kimwipe® to remove any liquid or smudges. Read and record the absorbance of each triplicate standard.
- Concentration = a + b * (absorbance). Determine the slope (b) and y-intercept (a) of a linear regression equation with concentration as the dependent variable (y) and absorbance as the independent variable (x).

**Calculations**

The bleaching (loss of purple color; reduction in absorbance) of the KMnO4 is proportional to the amount of oxidizable C in a soil sample. It is assumed that 1 mole (mol) MnO4 is consumed (reduced from Mn7+ to Mn^2+^) in the oxidation of 0.75 mol (9000 mg) of C.

Active C (mg/kg) = [0.02 mol/L - (a + b * absorbance)] * (9000 mg C/mol) * (0.02 L solution/0.0025 kg soil)

where:

0.02 mol/L = Initial solution concentration

(a + b * absorbance) = Post-reaction concentration

9000 mg C (0.75 mol) = Assumed amount to be oxidized by 1 mol of MnO4 changing from Mn^7+^ to Mn^2+^

0.02 L = Volume of KMnO4 solution reacted

0.0025 kg = Weight of soil used

**References**

Blair, G.J., R. Lefroy, and L. Lise. 1995. Soil carbon fractions based on their degree of oxidation, and the development of a carbon management index for agricultural systems. Australian J. Agric. Res. 46:1459–1466.

Blair, G.J., R. Lefroy, A. Whitbread, N. Blair, and A. Conteh. 2001. The development of the KMnO4 oxidation technique to determine labile carbon in soil and its use in a carbon management index. p. 323–337. In R. Lal, J. Kimble, R. Follet, and B. Stewart (eds.) Assessment methods for soil carbon. Lewis Publ. Boca Raton, FL.

Islam, K.R., and R.R. Weil. 2000. Soil quality indicator properties in mid- Atlantic soils as influenced by conservation management. J. Soil and Water Conserv. 55:69–78.

Weil, R.R., R.I. Kandikar, M.A. Stine, J.B. Gruver, and S.E. Samson-Liebig. 2003. Estimating active carbon for soil quality assessment: A simplified method for laboratory and field use. Am. J. Alternative Agric. 18(1):3–17.

# **Laboratory Method 7: Autoclaved Citrate Extractable (ACE) Protein Content**

Moebius-Clune, B.N., D.J. Moebius-Clune, B.K. Gugino, O.J. Idowu, R.R. Schindelbeck, A.J. Ristow, H.M. van Es, J.E. Thies, H. A. Shayler, M. B. McBride, D.W. Wolfe, and G.S. Abawi, 2016. Comprehensive Assessment of Soil Health – The Cornell Framework Manual, Edition 3.0, Cornell University, Geneva, NY. Available at [CASH-Standard-Operating-Procedures-030217final-u8hmwf.pdf (bpb-us-e1.wpmucdn.com)](https://bpb-us-e1.wpmucdn.com/blogs.cornell.edu/dist/7/9922/files/2021/11/CASH-Standard-Operating-Procedures-030217final-u8hmwf.pdf)

Based on Wright, S.F., and A. Upadhyaya. 1999. Quantification of arbuscular mycorrhizal fungi activity by the glomalin concentration on hyphal traps. Mycorrhiza 8: 283-285. <http://link.springer.com/article/10.1007%2Fs005720050247?LI=true>

Cited by <http://scholar.google.com/scholar?cites=7372525266975194944&as_sdt=5,28&sciodt=0,28&hl=en>

Method code: ACE

**Application**

Soil proteins are measured for two reasons. First, the measurement is an index of the size of the pool of organically bound nitrogen (N) in a soil. The pool consists of the major compound classes of soil organic matter derived from expected biomass inputs that are likely to contribute to N mineralization and subsequent plant uptake of N.

Second, it is a general indicator of the (re-)coupling of carbon (C) and N in a soil ecosystem. The soil proteins likely contribute to the immobilization, storage, and remineralization of plant-available N because they are abundant, have a relatively high molecular weight and stability, are enzymatically degradable by a wide variety of microbes, and have a low C:N ratio as compared to numerous other compound classes in mixed soil material. N cycling in a soil ecosystem that has a healthy, active biological community is strongly associated with C cycling. The microbial activity in the ecosystem is fueled by energy stored in organic compounds. When these compounds are broken down by microbial activity, they release N if the C:N ratio is sufficiently low. The organic N in the ecosystem is stored dominantly in the microbial biomass, and much of this N is stored as protein. A substantial fraction of the N in soil in the form of protein indicates that the processes involved in the storage and release of N in organic form are functioning.

The objective of this procedure is to extract protein from the organic matter in soil samples and to quantify the content of protein in the extract. The protein is extracted by using a neutral sodium citrate buffer to disaggregate soil and dissolve soil protein under high heat and pressure in an autoclave, and the content of protein is quantified by using a bicinchoninic acid protein assay. The extraction procedure is modified from an approach used to extract proteins from fungi and soil (Keen and Legrand, 1980; Wright and Upadhyaya, 1996) and has been shown to extract proteins from numerous sources. The quantification assay is a well-established procedure and chemistry. It is run at a high temperature for an **extended period** to increase the sensitivity of protein **measurement** and decrease variation by the type of protein (Walker, 2002).

**Summary of Method**

Proteins are extracted from soil samples in a sodium citrate solution that has been adjusted to neutral pH with citric acid. The sodium and citrate ions contribute to the dispersal of soil material, and it is dispersed further by mechanical agitation. When the soil is disaggregated, or slaked, the soil organic matter particles are exposed to the extractant solution. The mixture is exposed to high heat (121 °C) in an autoclave to further solubilize proteins. After the mixture is cooled, aliquots are clarified by centrifugation, which settles the soil particles. The concentration of dissolved proteins in the clarified extract is determined by reaction in a bicinchoninic acid assay at 60 °C, and it is quantified against a bovine serum albumin (BSA) standard curve by colorimetry using a 96-well spectrophotometric plate reader.


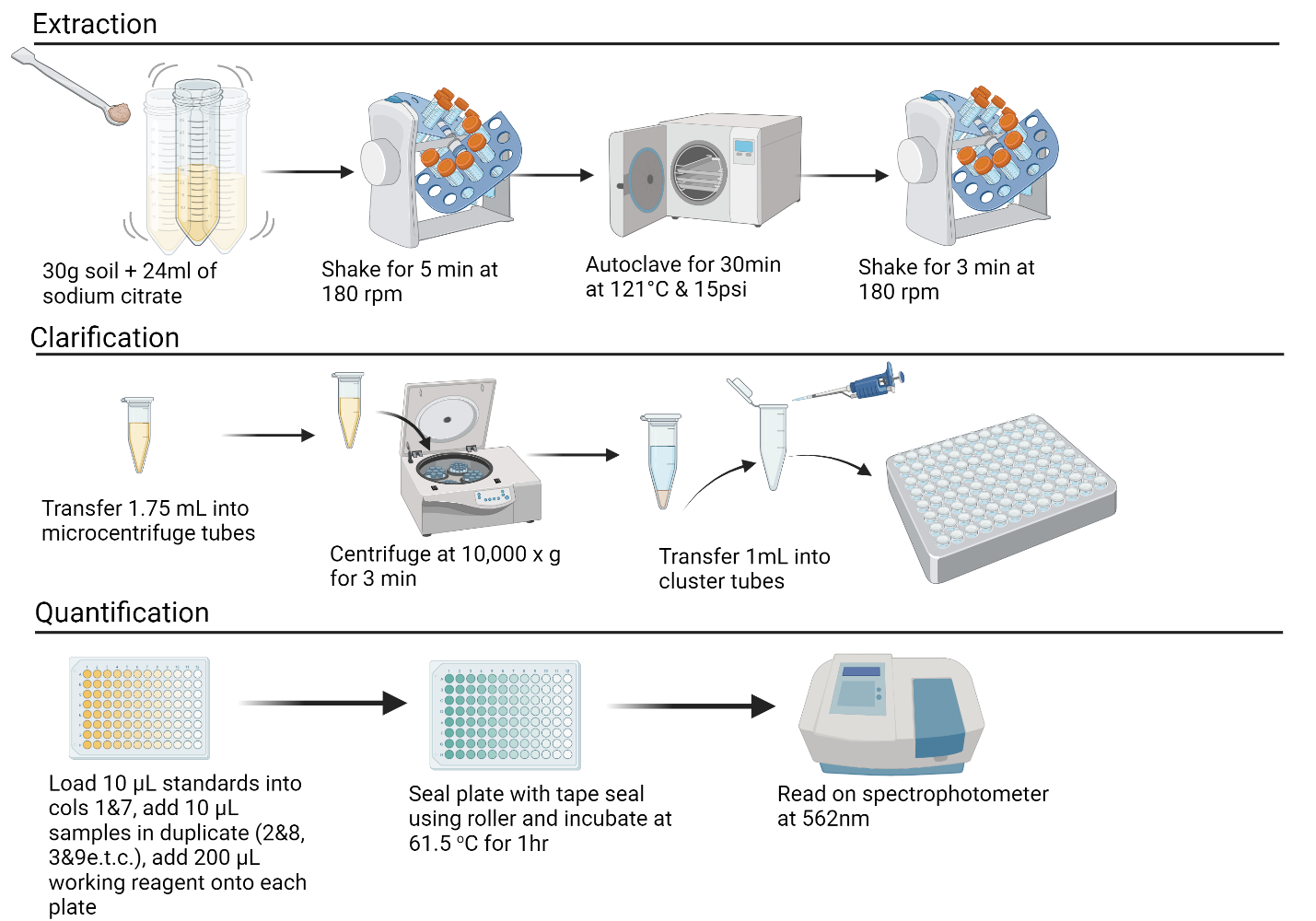


Illustrative pipeline of autoclaved-citrate extractable (ACE) protein by Ekundayo Adeleke

**Interferences**

- Scratches, smudges, dust, or other defects of the 96-well plate affect the spectrophotometric reading. Carefully inspect the plate before adding soil samples and reagents.
- For BSA standards aliquoted into microtiter tubes, use a different strip of caps each time tubes are recapped to avoid contamination of the standards set.
- Pipetting a 10-µl volume of a sample into the reaction plate has a high sensitivity to potential for error; therefore, it is necessary to ensure the full volume is drawn up and dispensed. Change pipette tip after each replicate.

**Safety**

Exposure to steam from the autoclaves can cause severe thermal burns. Use appropriate personal protective equipment (heat-resistant gloves, face shields, lab coat), and ensure the autoclave is safe to open when removing samples. Failure to loosen caps on extraction tubes before autoclaving can result in the tubes over-pressurizing and shattering in the autoclave.

**Collecting, Handling, and Processing of Soil Samples**

Ensure the soil sample collection bags are properly labeled. Keep collected samples cool and out of sunlight while in the field (preferably in a cooler).

After returning from the field, place samples in a cool area (refrigerator). Do not dry or freeze.

Process samples by air-drying in a cool location and sieving through an 2-mm screen.

Follow the established protocol for laboratory analysis.

**Materials**

- Soil sample (air-dried, sieved to 8 mm)
- Glass extraction tubes and caps
- 25- or 50-ml bottle-top reagent dispenser
- Microcentrifuge tubes
- Storage tubes in racks (1.1-ml, open-top tubes in strips of 8, racked in 96-place format)
- Strip caps for storage tubes
- Transfer pipettes
- Pipettors, 1000 µl, large and small volume, 8 channel
- 1000-, 200-, and 20-µl tips (utilize low retention tips, they maximize sample delivery)
- Multichannel pipetting reservoir
- 96-well, clear, flat-bottom chimney well, polystyrene plate
- Tape seal for plate

**Pre-assay Preparation**

*Prepare 1L of 20 mM sodium citrate (Na_3_C_6_H_5_O_7_) stock solution preparation*:

- Prepare 100 mL of 1M citric acid for adjustment of pH by adding 19.2g citric acid to 75ml of water in a volumetric flask (100 mL capacity), bring the volume to 100 mL by DI water.
- To make 20 mM sodium citrate, pH 7.0 (extraction buffer). Add a stir bar to a 1L beaker, add ~750 ml distilled water and slowly add 11.764 g tribasic sodium citrate dihydrate (Na_3_C_6_H_5_O_7_•2H_2_O, mw. 294.10) while spinning the mixture on low rpm. Bring the volume to 2L. Using a 1ml Pasteur pipette, adjust the pH of the solution to 7.0 with the previously prepared 1M citric acid.

**Method**

**Part 1: Extraction**

- Weigh 3.00 g <2-mm, air-dried soil sample. Transfer to glass extraction tube.
  Note: Two replicates are required for each soil sample. The ideal batch size is 42 glass tubes, which equates to 20 soil samples (done in duplicate), one control soil sample, and one blank. Set up tubes in racks suitable for use on a shaker and in an autoclave.
- Cap tubes, unless immediately proceeding to extractant step.
- Add 24.00 ml extractant (20 mM sodium citrate, pH 7.0) using a bottle-top dispenser.
- Cap tubes tightly after adding extractant.
- Briefly agitate the tube by hand (to ensure the soil-solution gets wets before homogenizing on the shaker).
- Shake at 180 revolutions per minute (rpm) for 5 minutes. While tubes are shaking, begin preheating the autoclave.
- Remove tubes from shaker. Swirl mixture in tubes to move solids that may have stuck to the sides down into the extractant.
- Loosen caps of tubes so they are not airtight but are on well enough to protect the contents. Failure to loosen caps may result in the tubes over-pressurizing and shattering in the autoclave.
- Autoclave at 121 °C and 15 pounds per square inch (psi) for 30 minutes.
- After door of autoclave is securely closed, turn knob to the 35-minute mark (timing empirically determined to expose to full temperature for 30 min)
- After samples have been autoclaved, allow the autoclave to cool so it can be opened safely.
- Wear heat-resistant gloves to remove racks of tubes. Set racks aside to cool to room temperature before clarification.

**Part 2: Clarification**

- Prepare a set of 2.0-ml microcentrifuge tubes (1 per glass extraction tube) and one or more racks of sample storage tubes (1.1-ml, open-top tubes in strips of 8, racked in 96-place format) to accommodate the range of samples to be clarified.
- Retighten caps on glass extraction tubes. Resuspend solids by shaking tubes for 1 minute at 180rpm.
- Swirl tube to move any solids on the side back into the solution (Be sure not to have any soil clinging to the side of the tube). Loosen or remove caps.
- Using a pipette, transfer approximately 1.75 ml from extraction tube into the pre-labeled 2-ml microcentrifuge tube. Close the lid.
- Place microcentrifuge tubes in microcentrifuge rotor slots, ensuring the microcentrifuge is properly balanced.
- Centrifuge at 10,000 x g for 3 minutes.
- Transfer 1 ml of the cleared extract liquid to a storage tube (microtiter tube) in a 96-well rack. Avoid dislodging the pellet of soils at the bottom of the tube.
- Cap tubes. Unless quantifying on the same day, set rack with tubes in refrigerator overnight.

***Pre-quantification preparation of standard***:

- BCA reagents A and B
- To make enough BCA working solution for one 96 well plate, use 25 mL of A and 0.5 mL of B.
- Prepare fresh solution before analysis. Dilute 0.5 ml BCA reagent B with 25 ml BCA reagent A. Mix thoroughly. Solution may be made in larger volumes at a ratio of 1:50.
- Bovine serum albumin (BSA) standards set (0, 125, 250, 500, 750, 1000, 1500, and 2000 µg/ml)

**Part 3: Quantification**

- Remove racks of BSA standards and sample microtiter tubes from refrigerator.
  Note: BSA standards should be aliquoted into smaller volumes in microtiter tubes. This minimizes the number of times they are refrigerated and brought to room temperature and reduces the amount of standard that would be discarded if contamination occurs.
- Before quantifying, allow tubes to equilibrate with room temperature.
- Inspect a 96-well reaction plate for scratches and other imperfections and for cleanliness.
- Preheat the heat block to 61.5 °C (depending on your product this may take up to 30mins).
- Make the BCA 50:1 working reagent. For 25.5 ml, add 0.5 ml reagent B to a 50-ml tube and then add 25 ml reagent A. Mix thoroughly.
- Remove strip caps from tubes that contain standards (0, 125, 250, 500, 750, 1000, 1500, and 2000 µg/ml BSA)
- Using an 8-channel, small-volume, multichannel pipettor and 20-µl tip, pipette 10 microliters of the BSA standards into the first and seventh columns of the reaction plate. Recap standards with new cap strips. Dispense the aliquot towards the bottom of the well. Use new tips for each well. For the 0 standard, use 20 mM sodium citrate from the same batch that you used to extract your samples. NOTE: It is CRITICAL not to cross-contaminate standards. Be extremely careful!
- Uncap samples, mix by pipetting “up and down” (aspirating and dispensing) before pipetting 10 µl into the appropriate columns of the plate.
- Pipette two replicate columns of each strip of eight sample tubes into the plate wells, following the first and seventh columns.

*Example of plate set up*:

|  | 1 | 2 | 3 | 4 | 5 | 6 | 7 | 8 | 9 | 10 | 11 | 12 |
| --- | --- | --- | --- | --- | --- | --- | --- | --- | --- | --- | --- | --- |
| A | **Std1** | 01A | 05A | 09A | 13A | 17A | **Std1** | 01A | 05A | 09A | 13A | 17A |
| B | **Std2** | 01B | 05B | 09B | 13B | 17B | **Std2** | 01B | 05B | 09B | 13B | 17B |
| C | **Std3** | 02A | 06A | 10A | 14A | 18A | **Std3** | 02A | 06A | 10A | 14A | 18A |
| D | **Std4** | 02B | 06B | 10B | 14B | 18B | **Std4** | 02B | 06B | 10B | 14B | 18B |
| E | **Std5** | 03A | 07A | 11A | 15A | 19A | **Std5** | 03A | 07A | 11A | 15A | 19A |
| F | **Std6** | 03B | 07B | 11B | 15B | 19B | **Std6** | 03B | 07B | 11B | 15B | 19B |
| G | **Std7** | 04A | 08A | 12A | 16A | 20A | **Std7** | 04A | 08A | 12A | 16A | 20A |
| H | **Std8** | 04B | 08B | 12B | 16B | 20B | **Std8** | 04B | 08B | 12B | 16B | 20B |

- When all samples (and standards) have been placed in the appropriate wells of the reaction plate, recap the samples and set aside.
- Transfer BCA 50:1 working reagent into a clean, dry, multichannel pipettor reservoir.
- Using a larger volume, multichannel pipette and 200-µl tip, add 200 µl BCA working reagent to each well of the reaction plate.
- When the plate is filled, apply a tape seal and secure using a tape seal roller.
- When plate is sealed, place in heat block and cover.
- Start 60-minute timer.
- When plate has incubated for 60 minutes, remove from heat block and place on benchtop to cool for 10 minutes.

*Reading plate*

- Turn on plate reader and computer, and open plate-reader software.
- When plate has cooled, ensure that the sealing tape is well in place. Invert the plate and re-right to incorporate the droplets that have collected on the tape seal.
- Remove the tape seal carefully to avoid splashing and contaminating wells with content of other wells.
- Place plate in tray of plate reader.
- Read plate at 562 nm wavelength without shaking or other special effects.
- When plate reading is complete, save the file generated by the plate-reader software.
- Note any samples for which the concentration exceeds the high standard for calibration. Reread these samples after diluting them with sodium citrate.

**Calculation**

Average the absorbance values for the reaction replicates of the same extract before calculating the protein concentration. Average the concentration values across replicate extractions of the same soil sample and determine percent relative standard deviation (RSD). If the relative standard deviation of replications exceeds 5 percent, flag the sample for rerun.

From the absorbance values, create a standard curve of the standards, by subtracting the buffer blank from all the samples. Please note: This curve is quadratic! It should look something like this for direct plot of non:


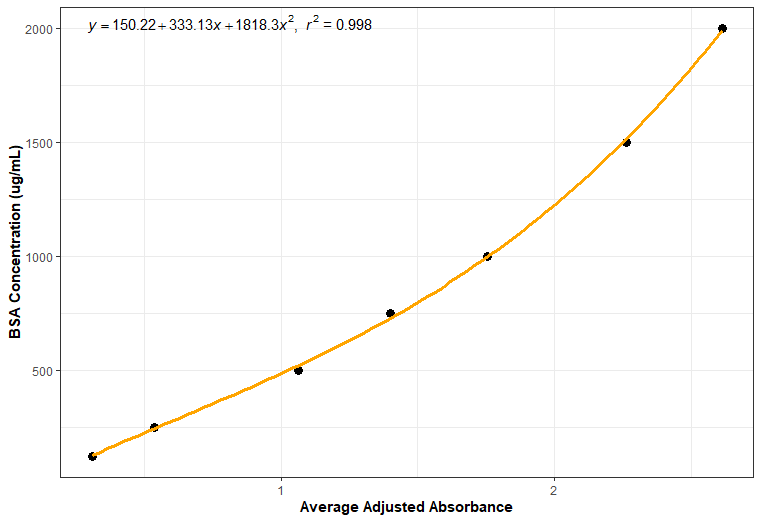


To determine the mass of protein from the amount extracted from the soil. Note, that this assay does not extract every soil protein from the soil however, it is a depiction of bioavailable pool that serves as a potential indicator in soil.

Use the following equation to determine protein content:

*ACE Protein* (mg. g-^1^ soil) = [(*a*x^2^ + *b*x + *c*) µg. mL^-1^ × 24 mL × 1mg] / (W g × 1000 µg)

Where:

*a* = coefficient of the x^2^ from the standard curve

*b* = coefficient of the x from the standard curve

*c* = intercept of the standard curve

x = difference between the average absorbance of the unknown and the blank

W = weight of air-dried soil sample

**Report**

Report protein concentration as g.kg-^1^ soil which is equivalent to mg. g-^1^ soil.

**Example Calculation**:

Construct standard curve using the following raw absorbance values from column 1 and 7 representing the values of the standards:


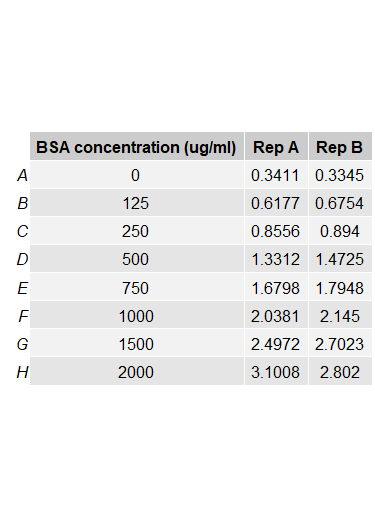


- Subtract the value of the blank from all the other values to determine the adjusted absorbance.
- Determine the average of the absorbance values in the first (Rep A) and second (Rep B) replicates.
- Removing the first row of zero value (0,0)
- This produces a table that looks like this:


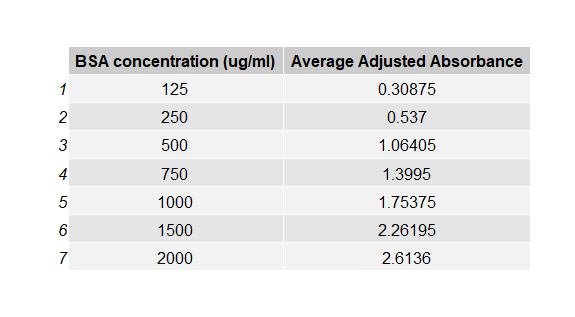


- This produces a parabolic (second order) regression line of best fit and the following quadratic equation:

*y* = 1818.3*x*^2^+ 333.13*x* + 150.22, r^2^ = 0.998

*To determine unknown sample absorbance*: 1.3685

*Average absorbance of blank*: 0.338

*Unknown sample weight*: 3.00g

*ACE Protein* (g. kg^-1^ soil) = [(1818.3(1.3685 – 0.338)^2^ + 333.13(1.3685 – 0.338) + 150.22) × 24 mL × 1mg] / (3.00g × 1000 µg)

= **19.4g Protein kg^-1^ soil**

**References**:

Keen N.T. and Legrand M. 1980. Surface glycoproteins - evidence that they may function as the race specific phytoalexin elicitors of Phytophthora megasperma f. sp. glycinea. Physiological Plant Pathology 17: 175-192.

Walker J.M. 2002. The bicinchonic acid (BCA) assay for protein quantitation. In: Walker J. M. (ed), The Protein Protocols Handbook. Humana Press, Totowa, NJ.

Wright S.F. and Upadhyaya A. 1996. Extraction of an abundant and unusual protein from soil and comparison with hyphal protein of arbuscular mycorrhizal fungi. Soil Science 161: 575-586.

# **Laboratory Method 8: Particle-Size Distribution Analysis**

Adapted from USDA NRCS. (2023). [Kellogg Soil Survey Laboratory Methods Manual, Soil Survey Investigations Report No. 42, Version 6.0, Part 1: Current Methods (usda.gov)](https://www.nrcs.usda.gov/sites/default/files/2023-01/SSIR42.pdf)

Method code: Sand_pct; Silt_pct; Clay_pct; Texture

**Sampling of Particle-Size Distribution Analysis Samples**

Process the air-dry soil by sieving to <2mm as described above. Briefly, before air-drying sample, weigh the sample to nearest g on a tared tray and record weight of the sample. Dry the samples in an oven at 30 – 35^o^C until constant weight, typically for 3 to 7 days depending on the moisture content of the field-moist sample. Weigh again after air-drying is completed and record weight.

Weigh subsample from airdried samples and record the weight to the nearest g. This weight includes the >2mm fraction. Crush clods of samples to pass through 2mm sieve. Crush clayey soils before passing through sieve to easy the sieving of the soil.

**Application**

Particle-size distribution analysis (PSDA) is a measurement of size distribution of individual particles in soil sample. These data of size distribution can be used to develop a cumulative distribution curve that have been used in different fields of study ranging from soil science, hydrology, geology, geomorphology to civil engineering. In USDA classification system, soil texture refers to relative proportion of sand, silt and clay based on the <2mm soil fraction.

Further evaluations, including the assessment of fine clay granulometric percentages, are employed to ascertain the presence of argillic horizons or to serve as aid in elucidating soil genesis.

**Summary of Method**

The standard method used for PSDA is via pipette method. This method utilizes a 10 g of <2mm air-dried sample that is pretreated to remove the organic matter and soluble salts. Briefly, the initial weight of air-dried sample is taken, dispersed with a sodium hexametaphosphate solution, and mechanically shaken. The sand fraction is removed from the suspension by wet sieving and then fractionated by sieving. Then the clay and fine silt fractions are determined using the suspension remaining the wet sieving process. After stirring the suspension that has been diluted to 1 L in a sedimentation cylinder, a 25-ml aliquot is removed with a pipette at an interval based on calculation of Stokes’ law. The aliquot is then oven-dried at 110 ^o^C and the weight is taken. Coase silt (an important fraction) is calculated based on difference between 100% and the sum of sand, fine silt, and clay.

**Interferences**

The cementing agents (like carbonates, iron oxides and silica) can hinder full dispersion in soil analyses. Pretreatments and dispersion methods are recommended for removal of cementing agents when necessary. Gypsum affects particle distribution by causing particle clumping and is removed by agitation and washing using reverse osmosis water; samples with over 25% gypsum need NaCl treatments. Excess salt or hydrogen peroxide can induce partial clumping in soils. Using H_2_O_2_ on micaceous soils leads to separation of the mica plates and leaves behind a matting particle after drying in the oven, making true fraction measurement uncertain. Removing carbonates with 1N sodium acetate (pH 5) acidifies samples and might damage the clay’s primary structure. Also, setting water bath over 80^o^C during iron removal can cause elemental Sulphur to precipitate. The use of 0.1 N sodium hydroxide to remove silica may damage 2:1 phyllosilicate clays or semi-amorphous clays.

**Safety**

Use appropriate personal protective material (lab coats, aprons, sleeve guards, gloves, face shields, and safety glasses) when handling acid and H_2_O_2_, and ensure to handle heated samples with leather gloves. Heat samples in ventilated fume hoods for removal of organic matter or cementing agents. Also, mix acids in ventilated fume hoods. If there is a spill, use sodium bicarbonate and water to neutralize and dilute the acids. Use and handle centrifuge according to manufacturer’s manual.

**Equipment**

- KIMAX 250-mL GL-45 bottle, tared to 1 mg. Wash and tare bottles every 6 months.
- Ceramic filter, 0.3-μm absolute retention
- Rack to hold ceramic filters and sample containers
- Mechanical shaker, horizontal, 120 oscillations min−1, 1½-in strokes
- Cylinders, 1-L, white line fused onto glass at 1-L mark
- Oven, 110 °C
- Hot plate, 100 °C
- Vacuum, capable of 0.8 bar (80 kPa)
- Thermometer, 0 to 150 °C
- Desiccator
- Motor driven stirrer. (The KSSL can be contacted for more information.)
- Cyclonic dampening hand stirrer that has a brass rod threaded at one end and a perforated plexiglass disk fastened to the threaded end to reduce sample vortex from mechanical stirrer. The rod should be slightly longer than the height of the settling cylinders. The plexiglass disk should be ½” narrower than inside diameter of the cylinders
- Adjustable pipette rack
- Lowy pipettes, 25-mL, with overflow bulb. (The KSSL can be contacted for more information.)
- Polyurethane foam pipe insulation that fits snugly around cylinder
- Sieve shaker with 12.7-mm (½ in) vertical and lateral movement at 500 oscillations min^−1^, accommodates nesting 2-$\frac{3}{8}$ inch sieves
- Weighing bottles, 90-mL, tared to 0.0001 g
- Drying dishes, aluminum
- Lab timer
- Electronic balance, ± 0.10-mg sensitivity
- Electronic balance, ± 1.0-mg sensitivity
- Watch glass, 65-mm diameter
- Evaporating dish, porcelain, 80-mm diameter, 32-mm height, with lip
- Centrifuge, floor model capable of 1,500 rpm
- Centrifuge bottles, 500-mL
- Torsion balance
- Manometer, hand-held, gauge and differential pressure, capable of 1,000

psi

- Gelatin capsules, 5-mL
- Machined PVC caps for threaded 90-mL weighing bottles, 3.2-cm (1¼ in) diameter with 1.1-cm (7/16 in) diameter hole drilled in center, O-ring seal
- O-rings, 3.2 x 38.1 mm (⅛ x 1½ in)
- Septa, rubber, 7.9-mm (5/16 in) diameter. Place in machined cap.
- Hypodermic needle, 25.4-mm (1-in), 23-gauge
- Mortar and pestle
- Stir rod with rubber policeman
- Set of 60-mm (2-3/8 in) sieves, square-weave phosphor bronze wire cloth

(except 300 mesh, which is twilled weave). U.S. series and Tyler Screen Scale equivalent designations are as follows:

| **Sand Size** | **Opening (mm)** | **U.S. No.** | **Tyler Mesh Size** |
| --- | --- | --- | --- |
| Very coarse sand (VCS) | 1.0 | 18 | 16 |
| Coarse sand (CS) | 0.5 | 35 | 32 |
| Medium sand (MS) | 0.25 | 60 | 60 |
| Fine sand (FS) | 0.105 | 140 | 150 |
| Very fine sand (VFS) | 0.047 | 300 | 300 |

**Reagents**

- Reverse osmosis (RO) water, ASTM Type III grade of reagent water
- Sodium hexametaphosphate (NaPO_3_)_6_ (CAS# 68915-31-1)
- Sodium carbonate (Na_2_CO_3_) (CAS# 497-9-8)
- Sodium Chloride (NaCl) (CAS# 7647-14-5), granular
- Hydrochloric acid (HCl) (CAS# 7647-01-0), concentrated, technical grade
- Ethanol 95% (CH3CH2OH) (CAS# 64-17-5)
- Hydrogen peroxide, 30 to 35% (H_2_O_2_) (CAS# 7722-84-1)
- Calcium sulfate (CaSO4) (anhydrous) or equivalent desiccant (example: Drierite)
- Sodium hexametaphosphate solution: Dissolve 35.7 g of (NaPO_3_)_6_ and 7.94 g of Na_2_CO_3_ in a 1 L polyethylene bottle containing 1L of RO water. Invert to mix
  - Designate weighing bottles for standardization only
  - Wash and tare these bottles after each standardization
  - Standards should be run in duplicate
  - Add aliquots of sodium hexametaphosphate solution to numbered, tared, 90-mL weighing bottles as: 8.5, 9.0, 9.3, 9.6, 10.0, 10.3, 10.6 and 11.0mL
  - Place the 16 weighing bottles and aliquots in the oven overnight and record the dry weight of the sodium hexametaphosphate solution
  - Calculate the specific volume of solution required to introduce 0.4408 g of sodium hexametaphosphate into each sample by correlating the solution volume with the dry residue weight of sodium hexametaphosphate.
- Hydrochloric acid (HCl) solution, 6N
  - Dilute concentrated HCl by adding 1 L of RO water to 2 L polyethylene bottle and thereafter add 1 L of concentrated HCl. Invert to mix
- Sodium carbonate (Na_2_CO_3_) solution
  - Dissolve 10.6 g Na_2_CO_3_ in 1 L of RO water and Invert to mix

**Procedure**

- Weigh 10 g of <2-mm, air-dried soil to the nearest mg using an electronic balance. Transfer the sample into a labeled, pre-weighed container. Incorporate a quality-control standard sample into every batch of up to 24 samples. For samples with a high percentage of organic content, divide the sample between two beakers, either 500 mL or 1,000 mL in volume.
- Add ≈50 mL of RO water and 7.5 mL of H_2_O_2_ to the soil sample
- Heat the sample to 90^o^C using a hot plate. Introduce additional H_2_O_2_ in four separate additions of 7.5 mL, spaced 30 minutes apart. If the organic matter hasn’t fully oxidized yet, continue to add H_2_O_2_ until the process is complete. Record any unusual reactions observed in the sample
- Position the sample vessel on the filter rack and fill it with RO water until the 150 mL indicator on the container. Introduce a ceramic filter into the sample, link it to the vacuum trap mechanism using a tube, and turn on the vacuum. Continue filtration until only about 5ml of liquid remains at the bottom of the vessel
- Rinse the sample off the filter, then redissolve in ≈150 mL of RO water. Continue the filtration and redissolving process until the sample has been rinsed a total of 5 times. Each time you add water, agitate the sample alongside the filter to guarantee thorough rinsing of all soil particles
- After completing the filtering process, place the sample in the oven. Dry the sample overnight at 110 ^o^C. Remove the sample from the oven, place in a desiccator, and cool to ambient temperature
- Record the total weight (TW) of the sample to the nearest mg
- Add the precise volume of the sodium hexametaphosphate solution (≈10mL) required to incorporate 0.4408g of sodium hexametaphosphate into each sample. Allow the sample to sit for ≈ 1 hour or until it’s fully saturated by the sodium hexametaphosphate solution. If the sample absorbs the hexametaphosphate, replenish with RO water until it is just moist. For hydrophobic samples, administer a few drops of ethanol. If samples don’t disaggregate as expected, use the rubber policeman to separate them. Gentle abrasion using a mortar and pestle can also aid in the process. Subsequently, fill with RO water up to the 175mL indicator on the vessel.
- Position the sample in a horizontal shaker at 120 oscillations min^-1^ and shake for 15 h (overnight)
- After removing the sample from the shaker, set up a 300-mesh (0.047-mm) sieve in a ring stand. Position a funnel into a 1-L graduated cylinder and arrange both beneath the sieve to capture the silt and clay fractions. Pour the sample through the sieve, ensuring all particles smaller than 20-µm are channeled into the cylinder. Continue this rinsing process into the volume of the suspension in the cylinder is approximately 800 mL. Top up the cylinder to the 1 L mark using the RO water rand the shield it with a 65-mm watch glass.
- Rinse the sand and the coarse silt from the sieve into an evaporation dish. Dry the contents at 110^o^C overnight. Once dried, record the weight.
- Position the cylinder on a steady bench top and wrap them with pipe insulation. This insulation assists in tempering any temperature fluctuations and settling velocities. Set u0p a cylinder filler with RO water as a blank control to monitor temperature variations. Let the cylinders sit overnight, allowing the suspension to stabilize and match the ambient room temperature
- Transfer the dried sand to a nest of sieves. Shake on sieve shaker for 3 minutes. Record the weight of each separate sand fraction (SWi) to the nearest mg
- Stir the silt and clay suspension with the mechanical stirrer for at least 5 minutes. Place the cylinder on a stable lab bench. With the cyclonic dampener, draw the rod up-and-down through the length of the cylinder for 30 s to eliminate swirling action caused by the mechanical stirrer
- Acquire fine silt and clay separates gravimetrically by using a Lowy 25-mL pipette mounted on an adjustable pipette rack (figs. 3A1a1–1, 3A1a1–2, and 3A1a1–3). Obtain an aliquot of <20-μm fraction sample from the cylinder based on the temperature of the blank and table 3A1a1a–1. Withdraw an aliquot at the calculated time in the table. Slowly lower the closed pipette to a depth of 10 cm below the surface of the suspension in the cylinder. Regulate the vacuum such that the pipette fills in ≈12 s
- Dispense the aliquot into a tared and numbered, 90-mL weighing bottle. Rinse the pipette twice with RO water, placing the rinse water into the tared weighing bottle.
- Wash and tare <2-μm employed bottles after every use. Wash and tare <20-μm bottles after every fourth use.
- Record temperature of the sample (T1) and the blank (T2). Use the average of T1 and T2 to adjust the pipette depth in the suspension as indicated in table 3A1a1a–2 for the <2-μm fraction pipetting. To obtain an aliquot of <2-μm size fraction, pipette after one of the following time intervals: 4.5, 5, 5.5, 6, or 6.5 h according to table 3A1a1a–2.
- Perform the aliquot withdrawal for the <2-μm size fraction the same as described for the <20-μm fraction. Regulate the vacuum such that the pipette fills in ≈12 s.
- Retain sample cylinder if optical mineralogy or fine-clay determinations are requested. Dry the aliquots at 110 °C overnight and cool in a desiccator. Record the weight of the residue (RW) to the nearest 0.1 mg.
- If optical mineralogy is requested, decant remaining sample suspension and transfer the sediment to a 400-mL beaker.
- Fill the beaker to a height of 5.5 cm. Stir the sediment and allow it to settle for 5 minutes. Discard the supernatant. Refill the beaker to a height of 5.5 cm. Stir again, allow to settle for 3 min, and then decant. Fill, stir, and decant after 2-minute settling. Repeat filling, stirring, and decanting until the top half of suspension is clear after the 2-minute settling time.
- Transfer the sediment to a labeled drying dish. Pour off excess water. Air dry. Save in the drying dish for optical mineralogy. Sediment is ≈20 to 50 μm.

**Calculations**

Clay (%) = 100 x [(RW2 – DW) x (CF / TW)]

RW2 = Residue weight (g), <2-μm fraction

DW = Dispersing agent weight (g) = (0.4408 / CF)

CF = 1,000 mL / DV

DV = Dispensed pipette volume

TW = Total weight (g), H_2_O_2_-treated, oven-dry sample

Fine Silt (%) = 100 x [(RW20 – DW) x (CF / TW)] – Clay (%)

RW20 = Residue weight (g) of <20-μm fraction

Sand (%) = Σ (Swi / TW) x 100

Swi = Weight of sand fractions (1.0, 0.5, 0.25, 0.1, and 0.047 mm)

Coarse silt (%) = 100 − (Clay (%) + Fine silt (%) + Sand (%))

Silt (total, %) = 100 – (Clay (%) + Sand (%))

≈ Fine Silt (%) + Coarse Silt (%)

% = weight percent

**References**

USDA NRCS. (2023). [Kellogg Soil Survey Laboratory Methods Manual, Soil Survey Investigations Report No. 42, Version 6.0, Part 1: Current Methods (usda.gov)](https://www.nrcs.usda.gov/sites/default/files/2023-01/SSIR42.pdf)

# **Laboratory Method 9: Bulk Density**

Adapted from USDA NRCS. (2023). [Kellogg Soil Survey Laboratory Methods Manual, Soil Survey Investigations Report No. 42, Version 6.0, Part 1: Current Methods (usda.gov)](https://www.nrcs.usda.gov/sites/default/files/2023-01/SSIR42.pdf)

Method code: Bulk_Density

**Sampling of Field Bulk Density Samples**

Bulk density is used to determine the bulk density value of a moist soil core of known volume. Bulk density can be used to convert the data from a weight basis to a volume basis, determining the coefficient of linear extensibility, estimate saturated hydraulic conductivity, and identify compacted horizons. This method is used to determine the bulk density of field-moist soil core of known volume.

**Application**

Field bulk density (Db_f_) offers the opportunity to obtain relatively cheaply bulk density information without the expense incurred to obtain water retention. Db_f_ is particularly useful if the soil layers are at or above field capacity and/or the soils have low extensibility and do not exhibit desiccation cracks even if below field capacity.

**Summary of method**

A metal cylinder is pressed or driven into the soil. The cylinder is removed extracting a sample of known volume. The moist sample weight is recorded. The sample is then dried in an oven and weighed.

**Interferences**

During coring process, compaction of the sample is a common problem. Compression can be observed by comparing the soil elevation inside the cylinder with the original soil surface outside the cylinder. If compression is excessive, soil core may not be a valid sample for analysis. Rock fragments in the soil interfere with core collection. Dry or hard soils often shatter when hammering the cylinder into the soil. Pressing the cylinder into the soil reduces the risk of shattering the sample. If soil cracks are present, select the sampling area so that crack space is representative of sample, if possible. If this is not possible, make measurements between the cracks and determine the aerial percentage of total cracks or of cracks in specimen.

**Safety**

Follow standard field safety precautions.

**Procedure**

- Record the empty core weights (CW).
- Prepare a flat surface, either horizontal or vertical, at the required depth in sampling pit.
- Press or drive core sampler into soil. Use caution to prevent compaction. Remove core from the inner liner, trim protruding soil flush with ends of cylinder, and place in air-tight container for transport to laboratory. Collect three soil cores per layer. If soil is too loose to remain in the liner, use core sampler without the inner liner and deposit only the soil sample in air-tight container. Moisture cans may also be pushed directly into a prepared face. For fibrous organic materials, trim sample to fit snugly into a moisture can.
- Dry core in an oven at 110 °C until weight is constant. Record oven-dry weight (ODW).
- Measure and record cylinder volume (CV).
- If sample contains rock fragments, wet-sieve sample through a 2-mm sieve. Dry and weigh the rock fragments that are retained on sieve. Record weight of rock fragments (RF). Determine density of rock fragments (PD).

**Calculations**

Db = (ODW – RF – CW) / [CV – (RF / PD)]

Db = Bulk density of <2-mm fabric at sampled, field-water state (g cm−3)

ODW = Oven-dry weight

RF = Weight of rock fragments

CW = Empty core weight

CV = Core volume

PD = Density of rock fragments

**References**

Grossman, R.B., and T.G. Reinsch. 2002. Bulk density and linear extensibility. p. 201–228. *In* J.H. Dane and G.C. Topp (eds.) Methods of soil analysis, Part 4. Physical methods. Soil Sci. Am. Book Series No. 5. ASA and SSSA, Madison, WI.

# **Laboratory Method 10: Water Content**

Adapted from USDA NRCS. (2023). [Kellogg Soil Survey Laboratory Methods Manual, Soil Survey Investigations Report No. 42, Version 6.0, Part 1: Current Methods (usda.gov)](https://www.nrcs.usda.gov/sites/default/files/2023-01/SSIR42.pdf)

Method code: Water_Content

**Sampling of Water Content Samples**

Soil properties generally are expressed on an oven-dry weight basis. The field sample is air-dried at 30 to 35 °C, crushed, and sieved to <2 mm. The weight of air-dry soil remains relatively constant. For air-dry/oven-dry (AD/OD) ratio analysis (required if any air-dry analysis is determined), select material for representative subsamples from at least five different areas on the plastic tray. Prepare a subsample of the air-dry, sieved <2-mm fraction in a 2-oz metal weighing can.

**Application**

The moisture content is expressed as a ratio of the air-dry weight to the oven-dry weight (AD/OD). This is used to adjust all results to an oven-dry basis, and if necessary to calculate the sample weight that is equivalent to the oven-dry weight. The AD/OD ratio, unless otherwise specified, is determined on a <2-mm sieved sample. If analysis requires sieving to smaller fractions (2-0.5mm for aggregate stability) or grinding to fine fractions (≈180µm for total C), the AD/OD ratio of <2mm is used in any required calculations for moisture content.

**Summary of method**

A sample is weighed, dried to a constant weight in an oven, and reweighed. The moisture content is expressed as a ratio of the air-dry weight to the oven-dry weight (AD/OD). Soil properties of gypsiferous/gypseous soils that are reported on an ovendry weight basis are converted to include the weight of the crystal water. When reporting the water content of gypsiferous/gypseous soils, the crystal water content must be subtracted from the total oven-dry water content. The AD/OD ratio is corrected to a crystal water basis when the gypsum content of the soil is ≥1%.

**Interferences**

Samples may not reach a constant weight when dried overnight. The most frequently used definition for a dry soil is the soil mass after it has come to a constant weight at a temperature of 100 to 110 °C (ASTM, 2012). Temperatures that are >50 °C may promote oxidation or decomposition of some forms of organic matter. Do not add moist samples to an oven that already contains samples unless the initial samples have been in the oven for at least 12 to 16 hours. Weigh samples within 30 minutes of the samples cooling. Samples may adsorb significant amounts of atmospheric moisture. The removal of structural water, most commonly in gypsum, can produce a positive error. When reporting the water content of gypsiferous/gypseous soils, the crystal water content must be subtracted from the total oven-dry water content. Gypsum, hydrous oxides, and amorphous material may be affected by heating.

**Equipment**

- Electronic balance, ± 1-mg sensitivity
- Oven, thermostatically controlled, 110 ± 5 °C
- Thermometer, 0 to 200 °C
- Tin dishes, 4.5-cm diameter x 3-cm height, with covers

**Health and Safety**

Use heat resistant gloves to remove weighing containers from a hot oven. No other significant hazard is associated with this procedure. Follow standard laboratory procedures.

**Procedure**

- Tare the moisture dishes. Record each sample number and associated dish number
- Add 10 to 20 g <2-mm, air-dry soil to each moisture dish for AD/OD determination. Weigh the dish plus the sample and record the weight to the nearest 1 mg. Place the sample dish in a drying oven at 110 ± 5 °C. Allow the sample to remain in the oven overnight (12 to 16 h)
- Remove the sample dish and allow it to cool before reweighing. Do not allow the sample dish to remain at room temperature for more than 30 min
- Record the oven-dry weight to the nearest 1 mg
- Discard the sample

**Calculations**

AD/OD ratio = AD / OD

AD = (Air-dry weight) − (Tin tare weight)

OD = (Oven-dry weight) − (Tin tare weight)

Water content (%) = [(AD − OD) x 100] / OD

AD = (Air-dry weight) − (Tin tare weight)

OD = (Oven-dry weight) − (Tin tare weight)

**References**

American Society for Testing and Materials (ASTM). 2012. Standard practice for description and identification of soils (visual-manual procedure). D 2488. Annual book of ASTM standards. Construction. Section 4. Soil and rock; dimension stone; geosynthesis. Vol. 04.08. ASTM, Philadelphia, PA.

# **Laboratory Method 11: Total Nitrogen (as part of total carbon, total nitrogen, and Sulfur analysis)**

Adapted from USDA NRCS. (2023). [Kellogg Soil Survey Laboratory Methods Manual, Soil Survey Investigations Report No. 42, Version 6.0, Part 1: Current Methods (usda.gov)](https://www.nrcs.usda.gov/sites/default/files/2023-01/SSIR42.pdf)

Method code: TN_pct

**Sample preparation**

For total nitrogen measured as part of CNS analysis by dry combustion, the field sample is air-dried at 30 to 35 °C, crushed, sieved to <2mm, and milled to pass an 80-mesh (177-micron) sieve. The weight of air-dry soil remains relatively constant, and biological activity is low during storage.

**Application**

Total N includes organic and inorganic forms. The total N content of the soil may be <0.02% in subsoils, 2.5% in peats, and 0.06 to 0.5% in surface layers of many cultivated soil (Bremner and Mulvaney, 1982). The total N data may be used to determine the soil C:N ratio, the soil potential to supply N for plant growth, and the N distribution in the soil profile. The C:N ratio generally ranges between 10 to 12. Variations in the C:N ratio may serve as an indicator of the amount of soil inorganic N.

**Summary of Method**

An air-dry (80 mesh, <180µm) sample is packed in a tin foil, weighed, and analyzed for total C, N, and S by an elemental analyzer. The elemental analyzer works according to the principle of catalytic tube combustion in an oxygenated CO_2_ atmosphere and high temperature. The combustion gases are freed from foreign gases. The desired measuring components (N_2_, CO_2_, and SO_2_) are separated from each other with the help of specific adsorption columns and are determined in succession with a thermal conductivity detector. Helium is the flushing and carrier gas.

**Interferences**

Contamination through body grease or perspiration must be avoided in sample packing. Substance loss after weighing should be avoided by exact folding of the sample into the tin foil. Air in the sample material (falsifying the N value) should be minimized by compressing the sample packing. Insufficient O_2_ dosing reduces the catalysts, decreasing their effectiveness and durability. Burnt sample substance that remains in ash finger falsifies the results of subsequent samples. WO_3_ is used as sample additive and combustion filling to aid combustion or bind interfering substances (alkaline or earth-alkaline elements, non-volatile sulfates).

**Health and Safety**

Exhaust gas pipes should lead into a ventilated fume hood. Aggressive combustible products should not be analyzed. Before working on electrical connections (adsorption columns) or before changing reaction tubes, the instrument must be cooled down and cooled off. Gloves and safety glasses should always be worn during operation and maintenance of instrument.

**Equipment**

Elemental analyzer with on-line electronic balance (0.1 ±mg sensitivity) and automatic sample feeder, Elementar vario EL Elementar vario EL III, and Elementar vario Cube, Elementar Analysensysteme GmbH, Hanau-Germany, and combustibles (Elementar Americas, Inc., Mt. Laurel, NJ; Alpha Resources Inc., Stevensville, MI) as follows:

- Quartz ash finger, quartz
- Quartz bridge
- Combustion tube
- Reduction tube
- Gas purification (U-tube, GL 18)
- Support tube (65 mm)
- Protective tube
- O2 lance (150 mm rapid N)
- Tin boats (4 x 4 x 11 mm)
- Tin foil cups

Computer, with vario EL software, Elementar Analysensysteme GmbH, Hanau-Germany, and printer

**Reagents**

- Sulfanilic acid, calibration standard, 41.6% C, 4.1% H, 8.1 % N, 27.7% O, and 18.5% S
- Copper sticks
- Corundum balls, high purity, alumina spheres, 3–5 mm
- Cerium dioxide, 1–2 mm
- Tungsten oxide powder, sample additive
- Tungsten trioxide granulate, combustion tube filling
- Quartz wool

**Procedure**

*Elemental Analyzer Set-up and Operation*

Refer to the manufacturer’s manual for operation and maintenance of the elemental analyzer. Conditioning of the elemental analyzer and determination of factor and blank value limit are part of the daily measuring routine. The following are only very general guidelines for instrument parameters for the various analytes in the CNS mode.

| **Instrument Parameters** | | |
| --- | --- | --- |
| **Temperature** |  |  |
|  | Furnace 1 | 1140 ^o^C |
|  | Furnace 2 | 850 ^o^C |
|  | Furnace 3 | 0 ^o^C |
|  | CO2 column | 85 ^o^C |
|  | SO2 column | 210 ^o^C |
|  | SO2 col. standby | 140 ^o^C |
| **Timing** |  |  |
|  | Flush | 5 s |
|  | Oxygen delay | 10 s |
|  | Autozero delay | 30 s |
|  | Integrator reset delay | 50 s |
|  | Peak anticipation N | 70 s |
|  | Peak anticipation C | 125 s |
|  | Peak anticipation S | 70 s |
| **Integrated Reset Delay for S** |  | 60 s |
| **Thresholds** |  |  |
|  | N peak | 3 mV |
|  | C peak | 3 mV |
|  | S peak | 3 mV |

**Elemental Analyzer Calibration and Analysis**

A calibration that covers the desired working range of each element is performed. The calibration test analyzes sulfanilic acid with each given element content at different weights. The PC program automatically computes the calibration function (linear, polynomial, or mixed). Calibration typically remains stable for at least 6 months. Re-calibration is recommended when the daily factor is outside the range of 0.9 to 1.1 or if components that influence the results (e.g., detector or adsorption column) have been exchanged. Changing the desorption temperature of adsorption columns can also require a re-calibration.

Add 0.100 g of tungsten oxide in tin foil and tare. A homogenized, fine-grind, air-dried soil sample is then packed in the tin foil, which is weighed (0.100 to 0.05 g) and placed into the carousel of the automatic sample feeder of the elemental analyzer. Sample weight is based on visual observation of the sample, related to element content, homogeneity, and combustion behavior of the sample. The sample weight is entered in the PC from an on-line electronic balance via an interface. A quality control (QC) sample is performed at a minimum of every 35 to 40 samples.

**Calculations**

C (%) = C_i_ x AD/OD

where:

C (%) = C (%), oven-dry basis

C_i_ = C (%) instrument

AD/OD = Air−dry/oven-dry ratio

N (%) = N_i_ x AD/OD

where:

N (%) = N (%), oven-dry basis

N_i_ = N (%) instrument

AD/OD = Air-dry/oven-dry ratio

S (%) = S_i_ x AD/OD

where:

S (%) = S (%) on oven-dry basis

S_i_ = S (%) instrument

AD/OD = Air-dry/oven-dry ratio

**Report**

Report the total N to the nearest 0.001% and total C and S to the nearest 0.01%

**References**

Bremner, J.M. and Mulvaney, C.S. (1982) Nitrogen-Total. In: Methods of soil analysis. Part 2. Chemical and microbiological properties, Page, A.L., Miller, R.H. and Keeney, D.R. Eds., American Society of Agronomy, Soil Science Society of America, Madison, Wisconsin, 595-624

# **Laboratory Method 12: Effervescence**

Adapted from USDA NRCS. (2023). [Kellogg Soil Survey Laboratory Methods Manual, Soil Survey Investigations Report No. 42, Version 6.0, Part 1: Current Methods (usda.gov)](https://www.nrcs.usda.gov/sites/default/files/2023-01/SSIR42.pdf)

Method code: Effervescence

**Sample preparation**

To perform effervescence or check for the presence of carbonate in samples, the field sample is air-dried at 30 to 35 °C, crushed, sieved to <2mm, and milled to pass an 80-mesh (180µm) sieve in a 20 mL glass vial.

**Application**

The distribution of CaCO_3_ are important factors affecting fertility, erosion, available water capacity, and genesis of the soil. The formation of calcic and petrocalcic horizons has been related to a variety of processes, some of which include translocation and net accumulation of pedogenic carbonates from a variety of sources as well as the alteration of lithogenic (inherited) carbonate to pedogenic carbonate (soil-formed carbonate through *in situ* dissolution and re-precipitation of carbonates) (Rabenhorst et al., 1991).

**Procedure**

To check for the presence of carbonates, use the prepared subsample above. Place 1 g of the air-dry fine-earth fraction in porcelain spot plate, add reverse osmosis water, and stir to remove entrapped air.

Add 1 N HCl to soil (method 3A2a1, reagent 6.2), observe amount of effervescence, and record as follows:

**None**. —No visual effervescence.

**Very Slight**. — Bubbles rise at a few points in the sample and consistently appear at the same point in either a steady stream of tiny bubbles or in a slower stream of larger bubbles. Do not mistake trapped air bubbles for a positive test. Generally, these air bubbles appear immediately after the addition of 1 N HCl.

**Slight**. — More small bubbles, and possibly a few larger bubbles, appear throughout the sample than with a very slight reaction.

**Strong**. — More large bubbles are evident than with a slight reaction. Often the reaction is violent at first and then quickly decreases to a reaction that produces many small bubbles.

**Violent**. — The sample effervesces violently. Many large bubbles appear to burst from the spot plate.

**References**

Rabenhorst, M.C., L.T. West, and L.P. Wilding. 1991. Genesis of calcic and petrocalcic horizons in soils over carbonate rocks. p. 61–74. In W.D. Nettleton (ed.) Occurrence, characteristics, and genesis of carbonate, gypsum, and silica accumulations in soils. Soil Sci. Soc. Am. Spec. Publ. No. 26. ASA and SSSA, Madison, WI.

# **Laboratory Method 13: Electrical Conductivity**

Adapted from USDA NRCS. (2023). [Kellogg Soil Survey Laboratory Methods Manual, Soil Survey Investigations Report No. 42, Version 6.0, Part 1: Current Methods (usda.gov)](https://www.nrcs.usda.gov/sites/default/files/2023-01/SSIR42.pdf)

Method code: EC

**Sample preparation**

The field sample is air-dried at 30 to 35 °C, crushed, and sieved to <2 mm. The weight of air-dry soil remains relatively constant.

**Application**

The electrical conductivity (EC) of soil can be used to predict the soils that have measurable amounts of soluble salts but also to predict the quantity and appropriate dilutions for salts analyses of those soils. When salt is <0.25 mmhos.cm^-1^ (dS.cm^-1^) soils are considered not salty.

**Summary of Method**

A soil sample is mixed with water and allowed to stand overnight. The EC of the mixture is measured using an EC meter. This EC value is used to indicate the presence of soluble salts (U.S. Salinity Laboratory Staff, 1954).

**Interferences**

Reverse osmosis deionized water is used to zero and flush the conductivity cell. The extract temperature is assumed to be 25 °C. If the temperature deviates significantly, a correction may be required.

Provide airtight storage of KCl solution and samples to prevent soil release of alkali-earth cations. Exposure to air can cause gains and losses of water and dissolved gases, significantly affecting EC readings.

**Equipment**

- Electronic balance, ±1.0-mg sensitivity
- Conductivity bridge and conductivity cell, with automatic temperature adjustment, 25 ±0.1 °C, Markson Model 1056, Amber Science, Eugene, Oregon
- Plastic cups, 30-mL (1 oz), with lids, Sweetheart Cup Co. Inc., Owings Mills, MD
- Dispenser, re-pipette or equivalent, 0 to 10 mL

**Reagents**

- Reverse osmosis (RO) water, ASTM Type III grade of reagent water
- Potassium chloride (KCl), 0.010 *N*. Dry KCl overnight in oven (110 ^o^C). Dissolve 0.7456 g of KCl in RODI water and bring to 1-L volume. Conductivity at 25 ^o^C is 1.412 mmhos.cm^−1^

**Procedure**

- Weigh 5.0 g of <2-mm, air-dry soil in a 30-mL (1 oz) condiment cup
- Add 10 mL of RO water to sample using a re-pipette dispenser
- Swirl to mix, cap, and allow to stand overnight
- Standardize the conductivity bridge using RO water (blank) and 0.010 *N* KCl (1.41 mmhos.cm^−1^)
- Read conductance of supernatant solution directly from the bridge
- Record conductance to 0.01 mmhos.cm^−1^

**Calculations**

- No calculations are required for this procedure.
- Use the following relationship to estimate the total soluble cation or anion concentration (meq.L^−1^) in the soil.

EC (mmhos cm^−1^) x 10 = Cation or Anion (meq.L^−1^)

- Use the following relationship to estimate the total soluble cation or anion concentration (meq.g^−1^ oven-dry soil) in the soil.

EC (mmhos.cm^−1^) x 20 = Cation (meq.g^−1^ soil)

EC (mmhos.cm^−1^) x 20 = Anion (meq.g^−1^ soil)

**Report**

Report prediction conductance to the nearest 0.01 mmhos.cm-1 (dS.m-1)

**References**

U.S. Salinity Laboratory Staff. 1954. Diagnosis and improvement of saline and alkali soils. L.A. Richards (ed.). USDA Agric. Handbook 60. U.S. Govt. Print. Office, Washington, DC.

# **Laboratory Method 14: Hydrogen-Ion Activity (pH)**

Adapted from USDA NRCS. (2023). [Kellogg Soil Survey Laboratory Methods Manual, Soil Survey Investigations Report No. 42, Version 6.0, Part 1: Current Methods (usda.gov)](https://www.nrcs.usda.gov/sites/default/files/2023-01/SSIR42.pdf)

Method code: pH

**Application**

Soil pH is one of the most indicative measurements of chemical properties. The pH is more than just acidity or basicity of soil. Depending on the predominant clay present in the soil, the pH could be used as an indicator of the base saturation. pH also serves as a critical factor affecting the bioavailability of most essential elements for plant uptake. However, pH value is affected by many factors ranging from inorganic and organic matter to soil to solution ratio.

**Summary of Method**

The pH is measured in soil-water (1:1) by measuring a 20-g soil sample mixed with 20 mL of reverse osmosis (RO) water (1:1 w:v) with occasional stirring. The sample is allowed to stand 1 h with occasional stirring. The sample is stirred for 30 s, and the 1:1 water pH is measured.

**Interferences**

The pH varies between the supernatant and soil sediment (McLean, 1982). Measure the pH just above the soil sediment to maintain uniformity. Clays may clog the KCl junction and slow the electrode response. Clean the electrode. Wiping the electrode dry with cloth, laboratory tissue, or similar material may cause electrode polarization. Rinse the electrode with distilled water and pat dry.

Atmospheric CO_2_ affects the pH of the soil: water mixture. Closed containers and nonporous materials will not allow equilibration with CO_2_. If critical work is being done, the partial pressure of CO_2_ and the equilibrium point must be considered at the time of pH determination.

**Equipment**

- Measuring scoop, handmade, ≈20-g capability
- Paper or plastic cup, 120-mL (4 fl. oz.), disposable
- Dispenser, 0 to 30 mL, Repipet or equivalent
- Beverage stirring sticks, wood
- Titration beakers, polyethylene, 250 mL
- Automatic titrator
- Combination pH-reference electrode

**Reagents**

- Reverse osmosis (RO) water, ASTM Type III grade of reagent water
- pH buffers, pH 4.00, 7.00, and 9.18, for electrode calibration

**Procedures**

- Use a calibrated scoop to measure ≈20 g of <2-mm or fine-grind, air-dry soil. If sample is moist, use calibrated scoop to achieve ≈20 g of air-dry soil.
- Place the sample in a 120-mL (4-oz) paper cup.
- Dispense 20 mL of RO water into sample and stir with the wooden beverage stirrer.
- Allow to stand for 1 h, stirring occasionally.
- Calibrate the pH meter using the pH 9.18, 7.00, and 4.00 buffer solutions.

**Report**

Report the 1:1 water pH to the nearest 0.1 pH unit.

**References**

McLean, E.O. 1982. Soil pH and lime requirement. p. 199–224. In A.L. Page, R.H. Miller, and D.R. Keeney (eds.) Methods of soil analysis. Part 2. Chemical and microbiological properties. 2nd ed. Agron. Monogr. 9. ASA and SSSA, Madison, WI.

# **Laboratory Method 15: Community Structure, Phospholipid Fatty Acids (PLFA)**

Method code: PLFA

**Sampling and Shipping of PLFA Samples**

Determine whether or not a soil sample is regulated by the USDA Animal and Plant Health Inspection Service (APHIS) <https://www.aphis.usda.gov/plant_health/permits/organism/soil/downloads/Fed-SoilRegs.pdf>. Soil samples regulated by APHIS need to be shipped separately from those not regulated by APHIS.

Label all samples with the following:

- state of origin,
- county of origin,
- study title,
- principle investigator,
- study site,
- sample identifier (information needed to identify sample), and
- sampling date.

**PLFA Samples Not Regulated by APHIS**

Take a representative subsample, 100 to 150 g or about 1/4 cup, of the soil sample. Place the subsample in a labeled, zippered plastic bag. Attach “Minimum Information Required for Shipping” to bag.

Keep samples cool by using refrigeration or frozen gel packs. Ship samples overnight with sufficient frozen gel packs to keep them cool until they reach the Soil Health Assessment Center (SHAC). The number of frozen gel packs needed varies with the amount of soil being shipped, the shipping container used, and the time of year the soil is shipped. Ship samples in a shippable cooler or a box lined with a tied-off garbage bag (to protect samples from damage during shipping). Avoid shipping over a weekend.

Before shipping, contact the SHAC to alert staff of pending shipment at the following—

573-882-0941 (office)

573-882-3704 (laboratory)

[brandtdk@missouri.edu](mailto:brandtdk@missouri.edu)

Send samples to—

Attn: Donna K. Brandt

Soil Health Assessment Center

3600 New Haven Road

Columbia, MO 65201

**PLFA Samples Regulated by APHIS**

Take a representative subsample, 100 to 150 g or about 1/4 cup, of the soil sample. Place the subsample in a labeled, zippered plastic bag. Attach “Minimum Information Required for Shipping” to bag. Place the sample in a second zippered plastic bag (secondary container).

Ensure the sample is free from foreign matter or debris, plants and plant parts including noxious weeds, and infestations of other macro-organisms such as insects, cyst nematodes, mollusks, and acari. Unauthorized material will be subject to re-export, or destruction, as will authorized material that is comingled with unauthorized material.

Review a copy of the Soil Health Assessment Center’s “Permit to Receive Soil” (permit number P330-17-00216) and include in all shipments. Attach a PPQ Form 550 label with clear tape to the exterior of each package being imported under the permit. Avoid shipping over a weekend.

Enclose the following information in each shipment—

Permittee name: Donna Kay Brandt

Permit number: P330-17-00216

Label number: PPQ Form 550

Before shipping, contact the SHAC to alert staff of pending shipment at the following—

573-882-0941 (office)

573-882-3704 (laboratory)

[brandtdk@missouri.edu](mailto:brandtdk@missouri.edu)

Send samples to—

Attn: Donna Kay Brandt

Soil Health Assessment Center

3600 E. New Haven Road

Columbia, MO 65201

**High-Throughput Neutral Lipid Fatty Acids (NLFA) and Phospholipid Fatty Acids (PLFA) Analyses of Soil**

This standard operating procedure (SOP) is from the laboratory of Jeff Buyer, USDA-ARS (Buyer and Sasser, 2012), with interpretations from the University of Missouri Soil and Plant Testing Laboratory courtesy of Donna Brandt.

**Major Equipment**

- Lyophilizer
- High-speed concentrator
- Ultrasonic cleaning bath
- Multichannel pipettor reagents

**Bligh-Dyer Extractant**

- 200 mL 50-mM PO_4_ buffer pH 7.4 (8.7 g K_2_HPO_4_ per liter)
- 500 mL methanol
- 250 mL chloroform
- Mix fresh daily, or mix fresh weekly if many runs are anticipated.

**Transesterification Reagent**

- 0.561 grams KOH
- 75 mL methanol
- 25 mL toluene
- Dissolve KOH in methanol and then add toluene
- Prepare weekly.

**Internal Standard**

- Phospholipid: 1,2-dinonadecanoyl-sn-glycero-3-phosphocholine, Avanti Polar Lipids Catalog #850367P (white powder)
- Neutral lipid: Trinonadecanoin glyceride, Nu-Check-Prep Catalog #T-165
- Dissolve 40.9 mg phospholipid and 31.1 mg neutral lipid in 10 mL chloroform. Bring to 20 mL with methanol (2.5 mM solution of phospholipid and 1.67 mM neutral lipid).
- Store at −20 °C.
- Just before extracting, warm to room temperature. Add to an appropriate volume of extractant at a rate of 0.5 μL internal standard per mL of extractant and mix. This is equivalent to adding 10 nmoles 19:0 phospholipid and 10 nmoles 19:0 neutral lipid.

**Soil Drying**

- Weigh 95 13×100-mm screw-cap glass test tubes (without caps on).
- Add 1.5 to 2.0 g soil to each test tube. If using a lyophilizer, freeze test tubes.
- Run overnight in a high-speed concentrator (e.g., SpeedVac®) at room temperature, or use a freeze-dryer or lyophilizer. Lyophilization is preferred because tubes occasionally break in a high-speed concentrator.
- Weigh tubes with dry soil to calculate dry weight of soil.
- If storing samples before extraction, cap each tube with a Teflon-lined screw cap and store in a freezer at −20 °C.
- If working with previously dried soil, simply weigh 1 to 2 g into test tube.

**Extraction**

- - Use one additional test tube as a blank.
- Add 4 mL Bligh-Dyer extractant that contains internal standard.
- Sonicate 10 minutes in an ultrasonic cleaning bath at room temperature.
- Incubate at room temperature 2 hours with end-over-end shaking.
- Centrifuge 10 minutes in SpeedVac® (without vacuum).
- Transfer liquid phase to a 13×100-mm test tube that has a polytetrafluoroethylene (PTFE)-lined screw cap.

**Separation**

- Add 1 mL each of chloroform and deionized water to the test tube.
- Vortex 10 seconds and then centrifuge 10 minutes in high-speed concentrator (without vacuum).
- Aspirate top (aqueous) phase.
- Concentrate to dryness in test tubes at 30 °C (about 1 hour).
- Dissolve in 1 mL chloroform for chromatography.

**Lipid Separation**

- Use a 50-mg, silica gel, solid phase extraction (SPE), 96-well plate.
- Wash each well three times with 1 mL methanol and then 3 times with 1 mL chloroform.
- Place a clean 1.5-mL, multi-tier microplate in the bottom of the 96-well plate.
- Add extract to wells.
- Let sample drain into column and collect the eluate (NLFA fraction 1). Seal with a Teflon or silicone cap mat.
- Using another clean 1.5-mL, multi-tier microplate, repeat transfer with another 1 mL chloroform and collect the eluate (NLFA fraction 2). Seal with a Teflon or silicone cap mat.
- Wash with 1 mL chloroform and 1 mL acetone. Discard the eluate.
- Place clean 1.5-mL, multi-tier microplate in bottom of manifold of 96-well plate.
- Elute phospholipids with 0.5 mL 5:5:1 methanol:chloroform:H2O. Seal with Teflon or silicone cap mat. Store at 4 °C while processing the NLFA.
- Concentrate both NLFA fractions at 37 °C to approximately half the original volume (about 30 minutes).
- Transfer remaining volume in fraction 2 to fraction 1 using a multichannel pipettor. Use the high-speed concentrator to take the sample to dryness (37 °C, 1 hour). Seal with Teflon or silicone cap mat. Store at −20 °C.
- Use the high-speed concentrator to take the 5:5:1 fraction (PLFA) to dryness (70 °C, 30 minutes, and then 37 °C until dry, about 2 hours total). Seal with Teflon or silicone cap mat. Store at −20 °C.

**Transesterification and Transfer to Gas Chromatograph** **(GC) Vials**

- Carry out the entire procedure on either the NLFA or PLFA first. Once complete, carry out the other procedure.
- Let samples warm to room temperature. Add 0.2 mL transesterification reagent and mix.
- Incubate 37 °C for 15 minutes.
- Add 0.4 mL 0.075 M acetic acid and 0.4 mL chloroform.
- Seal with Teflon or silicone cap mat. Shake vigorously. Let separate.
- Transfer from bottom 0.3- to 1-mL multi-tier plate (E & K Scientific #EK-99234) using multichannel pipettor. If 1-mL tips displace too much volume or don’t fit in wells, use two 150-μl transfers that have 250-μl pipette tips.
- Repeat with another 0.4 mL chloroform and transfer bottom 0.4 mL this time. If 1 mL tips displace too much volume or don’t fit in wells, use two 200-μl transfers that have 250-μl pipette tips.
- If any aqueous phase is on top of the transferred chloroform, remove with a clean, disposable pasteur pipette. Evaporate the chloroform in the high-speed concentrator at room temperature and remove from concentrator as soon as dry (about 45 minutes).
- Redissolve extract in 75-μL hexane.
- Transfer to limited-volume insert placed in GC vial. Insert a PTFE/silicone septa in cap, and screw cap on vial.

**Gas Chromatography**

Use a gas chromatograph (GC) equipped with an autosampler, split-splitless inlet, and flame ionization detector. Consult the analytical methods for the GC regarding details on the separation of fatty acid methyl esters (FAMEs). For example, using an appropriate column, Buyer and Sasser (2012) used a split ratio of 30:1 with the hydrogen carrier gas at a constant flow rate of 1.2 mL/minute. The oven temperature is set at 190 °C initially, ramped to 285 °C at 10 °C/minute, then to 310 °C at 60 °C/minute, and held at 310 °C for 2 minutes. The injector temperature is 250 °C, and the detector temperature is 300 °C. The GC should have appropriate software for identifying microbial peaks.

**Glassware Cleaning**

- Scrub all glassware carefully with detergent and thoroughly rinse while wearing gloves.
- Use of an ultrasonic cleaning bath is helpful. Lipids form a monolayer and spread over the entire surface of wet glass, so wearing gloves is absolutely necessary.
- If possible, bake glassware at 400 to 500 °C at least 2 hours.
- Use a muffle furnace dedicated to clean glassware. Never put samples in the furnace.
- Shake all screw caps with hexane in test tube. Make sure Teflon liner is in place before using caps.
- Clean cap mats by gently scrubbing with soap and water. Rinse sequentially with deionized water, ethanol, and chloroform. Dry under a laminar flow hood.

**Notes**

- Use organic solvents that are graded high performance liquid chromatography (HPLC) or better.
- Run at least one blank with every batch of samples.
- Always wear gloves. Nitrile gloves may be better suited than latex gloves.
- GC caps must have PTFE/silicone septa.
- Test tube caps must be lined with PTFE.
- Limited-volume inserts must not have polyspring feet.

**Guide to Understanding Phospholipid Fatty Acid (PLFA) Data and Initial Exploratory Analyses**

**Step-by-Step**

1. First, refer to biology texts, scientific literature, and other sources readily available online or at a library for basic information on PLFAs, PLFA nomenclature, and PLFA extraction methods (e.g., [Buyer and Sasser, 2012](#_ENREF_3); [Tunlid and White, 1992](#_ENREF_16); [Zelles, 1999](#_ENREF_18); [Zelles et al., 1992](#_ENREF_19)). The document by Zelles (1999) describes PLFA versus neutral fatty acids, non-ester-linked fatty acids, etc.
2. Use this guide to aid in understanding and exploring PLFA output files from the Soil and Plant Testing Laboratory.
3. Consult the scientific literature pertinent to a particular study (e.g., similar management or ecosystems) for further information and ideas on interpretation of PLFA data. This helps to put the results in context.
4. Consult with an advisor or committee members to determine an appropriate analytical approach. The exploratory techniques listed in this guide are a starting point for that process. Many ideas are in PLFA literature (other than references cited in this document). Ultimately, the final data and statistical analyses depend on the objectives of a study, the study design, the quality and quantity of the data, and the skillset of the analyst (student, advisor, or other collaborator, such as a statistician).
5. Many criticisms of PLFA analyses, interpretations, and conclusions are documented in literature. Students should familiarize themselves with the pros and cons of the data and the analyses (e.g., [Frostegård et al., 2011](#_ENREF_6); [Kaur et al., 2005](#_ENREF_9)).
6. This guide is specific to the Buyer and Sasser extraction method and the Sherlock® Microbial Identification System (MIS) software of the MIDI Corporation. Peak assignments, microbial groupings, etc., vary in the literature.

**General PLFA Nomenclature**

Phospholipid fatty acids (PLFA) are an essential structural component of all microbial cellular membranes. The PLFA nomenclature generally is in a pattern of A:BωC and is defined as follows:

- The letter “A” identifies the total number of carbon atoms in the fatty acid.
- The letter ‘‘B’’ identifies the number of double bonds.
- The letter “C” designates the carbon atom from the aliphatic end before the double bond.
- The symbol “ω” (omega) denotes that the positions of the double bonds (“C” positions) are counted relative to the methyl group end instead of from the carboxyl group end (“front” end). For example, counting from the omega end, 18:2 ω6,9c has two double bonds at carbons 6 and 9 and 18:3 ω3,6,9c has 3 double bonds at carbons 3, 6, and 9.
- A letter “c” designates cis configuration of monoenoics and a “t” designates trans configuration of monoenoics (i.e., only one double bond).
- The letters “br” designate branching.
- The letters “i” and “a” designate iso and anteiso, respectively.
- The letters “me” designate midchain branching.
- The letters “cy” or “cyclo” designate cyclopropyl fatty acids.

The position of hydroxy groups are noted. Saturated fatty acids can be straight or branched chains and have no double bonds. That is, the chain of carbon atoms is fully saturated with hydrogen atoms. Saturated straight chains are designated as 12:0, 13:0, 18:0, etc., and are ubiquitous. Monounsaturated fatty acids (MUFA) have only one double bond (e.g., 16:1 ω7c), and polyunsaturated fatty acids (PUFA) have more than one double bond (e.g., 18:2 ω6,9c or 18:3 ω3,6,9c).

**Examples of PLFA Structures**

Straight-chain Saturated Fatty Acid


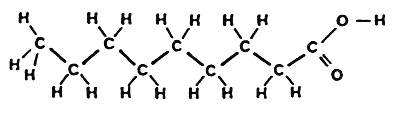


10-Methyl Branched Saturated Fatty Acid


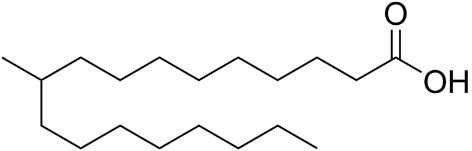


Trans-MUFA
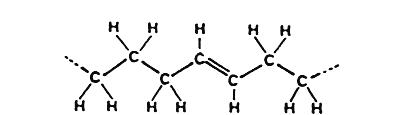


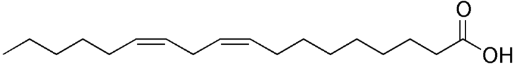
Cis-MUFA


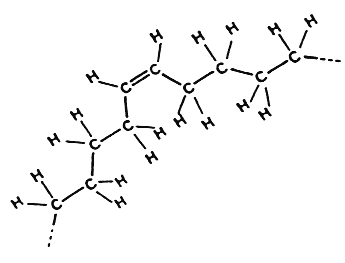


PUFA

Cyclopropyl Fatty Acid
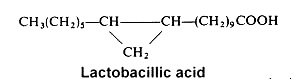


**Files Obtained from the University of Missouri Soil and Plant Testing Laboratory**

For alternative file formats, please contact the laboratory manager, Donna Brandt, at 573-882-0941. For example, she can provide Microsoft Access files, original chromatograms, and other files. By appointment, she also can assist onsite in the use of the Sherlock® Microbial Identification System (MIS) software, Version 6.0 (MIDI Corporation, Newark, NJ) to modify the plots, etc. If Donna isn’t able to answer the phone, please leave a voicemail and she will return the call.

The PLFA extraction procedure used by the laboratory follows the extraction method of Buyer and Sasser ([2012](#_ENREF_3)). Due to the high throughput of this extraction method and the subsequent GC analysis (Sherlock® software), peak resolution is reduced and isomers such as cis and trans cannot be resolved. Also, only ester-linked PLFAs are identified. Therefore, key biomarkers of archaea and certain anaerobic bacteria are not extracted or detected ([Kaur et al., 2005](#_ENREF_9)).

If a PLFA marker or microbial group is not identified in any of the samples in a dataset, no column for that marker or group will be in the file. If a marker or group is detected in some samples but not all, the cell will be blank for samples in which the marker or group was not detected. Note: If statistical analyses is being conducted, it is important to be cognizant of how the software (SAS, R, Matlab, etc.) handles blank cells. Some software treats blank cells as missing data. If preferred, talk to Donna Brandt about replacing blank cells with zeros.

1. [**WT_wt.xlsx**] Quantity of individual PLFA peaks in pmol/g dry soil.—This is the master peak file. The name of each peak (abbreviation) is given at the top of each column. The total PLFA content (the sum of all peaks in pmol/g soil) has been shown to correlate with methods for estimating microbial biomass ([Tunlid and White, 1992](#_ENREF_16); [Zelles et al., 1995](#_ENREF_20)) and can be used as an estimate of total microbial biomass for each sample. This file also provides the total number of peaks identified in each sample. Analysis of variance (ANOVA) can be used to compare the total PLFA content across treatments. This file is also useful to look at specific peaks (markers) or peak ratios that are not provided in the general output. An overall “global” read of the file can detect patterns or data that stand out. It may be preferable to convert from pmol/g to nmol/g (divide by 1000).
2. [**WT_%.xlsx**] Proportion of total response in mol % for individual PLFA peaks.—The name of each peak (abbreviation) is given at the top of each column. The sum of these values equals 100 percent for each sample. It may be beneficial to look in depth at the proportions of specific peaks of interest in each sample. For example, ANOVA can be used to compare the proportions across treatments.
3. [**MC_wt.xlsx**] Microbial groups in pmol/g dry soil.—Groups include Gram Positive (G+), Gram Negative (G-), Anaerobe, Actinomycetes (now called Actinobacteria), Methanobacter, Fungi, and AM Fungi. These values are an estimate of the microbial biomass in each microbial group for each sample. Note: An exact conversion of PLFA mass to group biomass does not exist. Some authors provide conversion factors, but large species-specific variation coupled with a lack of knowledge about species composition and the effects of environmental and growth stages make these conversion factors questionable. The MIDI software automatically assigns each PLFA peak to one of the microbial categories and then sums the peaks for each microbial type. The PLFA mass of specific groups can be compared across treatments in a study. For example, use ANOVA to compare the Actinobacteria PLFA mass (in pmol/g or nmol/g) across multiple treatments. These values can be used to calculate ratios, such as that of G+ to G-. The general (“None” or “Nonspecific”) category is not shown in this output. A description of microbial groups and some common ratios used in the literature are provided later in this section. To avoid redundancy or counting of peaks twice, the subcategories are not included in the sum for the overall category. For example, Actinobacteria are G+, but the Actino marker is not counted in the G+ category. Similarly, AMF are fungi, but the AMF marker is not included in the Fungi category.
4. [**MC_%.xlsx**] Proportion (mol %) of each microbial group.—The sum of all assigned microbial groups equals 100 percent (“None” category not included). The name of each microbial type is given at the top of each column. The PLFA mass of specific groups can be compared across treatments in a study. For example, use ANOVA to compare the Actinobacteria PLFA mass (pmol/g or nmol/g) across multiple treatments. The proportions of these groups may be affected by treatments (not just the estimated biomass given in the corresponding [**MC_WT.xlsx**] file).
5. [**FT_WT.xlsx**] PLFA fatty acid types in pmol/g dry soil.—Groups include Straight, Branched, MUFA, PUFA, Cyclo, Hydroxy, 10-methyl, DMA, 16:1 ω5c, 18:1 ω9c, 18:2 ω6,9c, and Other. These values are an estimate of the biomass of each fat type in a sample. The MIDI software automatically assigns each PLFA peak to one of the categories of fatty acids and then sums the peaks for each fat type. The biomass of specific fats can be compared across treatments in a study. The types of fatty acid commonly are associated with specific microbial groups or different growth phases.
6. [**FT_%.xlsx**] Proportion (mol %) of each PLFA fatty acid type.—The sum of all assigned fat types equals 100 percent (“Other” category included). The name of each fat type is given at the top of each column. The proportion of specific fat types can be compared across treatments in a study. For example, use ANOVA to compare the percent MUFA (monounsaturated fatty acids) across multiple treatments. PLFA fat types include Straight, Branched, MUFA, PUFA, Cyclo, Hydroxy, 10-methyl, DMA, 16:1 ω5c, 18:1 ω9c, 18:2 ω6,9c, and Other.

**Miscellaneous qualitative multivariate-type MIDI output files**

“Cluster” is a Sherlock® applet that allows users to find groups of samples that are closely related to each other. These groups are referred to as clusters. Sherlock algorithms use Euclidean distance to determine proximity (similarity of profiles determined by Euclidean distance between two profiles). The smaller the number, the more closely related. Various visualization and reporting tools can be used to examine the clusters. These plots are an initial, visual indication of the relationships among the samples. For thesis or journal-quality plots or analyses, the student may have to produce their own plots. Some plot data can be downloaded into Excel for use in other plotting programs. Please contact Donna Brandt at the Soil and Plant Testing Laboratory for more information on these plots and the associated data. Some of the exploratory output files provided by the laboratory include:

- [**NJT.rtf**] = Neighbor joining tree
- [**Dendrogram.rtf**] = Dendrogram
- [**PC.rtf**] or [**Excel_PC.xlsx**] = 2D principal components plot (3D not currently available in Sherlock® software)

**Other exploratory data analyses**

1. Standard ANOVA techniques following the experimental design of the study.
2. Regression/correlation analysis using PLFA markers (correlations among PLFA markers) and with other soil properties (SOC, total nitrogen, enzyme activity). These relationships can be used as supporting information for other results or conclusions.
3. Plots such as pie charts, bar graphs, etc., can help to visualize data.
4. The Shannon Diversity Index, Pielou’s Evenness Index, or Richness Indices can be calculated. Read the literature before applying these methods, and use care in applying them ([Frostegård et al., 2011](#_ENREF_6); [Kaur et al., 2005](#_ENREF_9)).
5. Multivariate analyses such as Partial Least Squares Regression (PLSR) or the more sophisticated Bayesian approaches, etc.
6. Other ideas can be gleaned from the literature. Use your imagination and try different things!

**Interpretations of Microbial Categories Assigned to Individual or Collective PLFA Biomarkers**

**Microbial Groups**

(Some are identified by MIDI, and some are not.)

Recall: These values do not reflect absolute biomass values since no conversion factors from PLFA concentration to actual biomass are applied.

Gram-negative (G-) bacteria.—These bacteria are a major component of the plant rhizosphere. They improve plant growth by increasing solubility of many nutrients. Some G- bacteria, such as Rhizobium, form mutually beneficial relationships with plants (legumes) and fix atmospheric nitrogen to produce ammonium, which is a form of nitrogen plants can use. G- bacteria are smaller than G+ bacteria, and their cells do not retain the crystal violet stain used in Gram staining. Generally, G- bacteria are dominant in the surface soil layer and break down newly added organic matter and G+ bacteria are dominant in the rooting zone. G- bacteria cannot form endospores; thus, they may be more sensitive to water stress than are G+ bacteria. G- bacteria produce MUFA and cyclopropane PLFAs. The MIDI software automatically assigns peaks (63 possible) to this category.

Gram-positive (G+) bacteria.—These bacteria are common in the bulk soil, but are concentrated in the rhizosphere. Generally, G+ bacteria are dominant in subsurface layers (versus G-) but they are not as closely tied to the rhizosphere as are G- bacteria. G+ bacteria are larger than G- bacteria, and their cells retain the crystal violet stain used in Gram staining. G+ bacteria are widely dispersed in soil and tend to further decompose organic material that has been partially decomposed by fungi or G- bacteria. G+ bacteria are resistant to water stress because they can form endospores, which allows them to remain dormant for years during unfavorable environmental conditions. The PLFA profiles of G+ species have high percentages of saturated, branched-chain PLFAs such as 15:0iso and 15:0anteiso. Thus, the sum of iso and anteiso PLFAs provides an estimate of the abundance of the G+ bacteria (other than Actinobacteria) in a sample. The MIDI software automatically assigns peaks (24 possible) to this category.

Anaerobic bacteria.—These bacteria are important under conditions of low oxygen, such as in wet soils, deep soils, sediments, and the interior of soil macroaggregates. Therefore, they may be present in surface soil material. Some can only survive under an absence of oxygen (obligate anaerobes). Some anaerobes reduce nitrogen compounds in the soil and release elemental nitrogen to the atmosphere. Anaerobic bacteria can be either G+ or G-. Dimethyl acetals (DMA), such as 16:1 ω9c (1,1-Dimethoxy-7-Hexadecene), are considered PLFA biomarkers for anaerobic bacteria. The MIDI software automatically assigns peaks (17 possible) to this category.

Actinobacteria (formerly Actinomycetes).—These are G+ bacteria that are active in the decomposition of organic matter. They produce geosmin, which is the compound that generates the “earthy” smell of freshly tilled soils. These bacteria grow in branching, thread-like patterns similar to that of fungal hyphae. They are filamentous, which allows them to bridge gaps between water films and withstand water stress ([Moore-Kucera and Dick, 2008](#_ENREF_12)). They reproduce by spores and fragmentation (similar to fungi). They break down soil organic matter, including resistant compounds such as cellulose, chitin, and lignin. Actinobacteria can grow deep in a soil, but they are sensitive to acidic, low-oxygen, and wet or saturated conditions. Some species are heat-resistant (thermophilic) and are common in compost. These species have distinctive PLFAs with a methyl branch at the 10th carbon, such as 10Me16:0 (10-Methylhexadecanoic acid/10-Methylpalmitic acid), 10Me18:0, and others. The MIDI software automatically assigns peaks (7 possible) to this category.

Methanotrophs.—These bacteria are sometimes called methanophiles or methane-oxidizers. They are prokaryotes that can metabolize methane for carbon and energy. They are primarily G- bacteria. They do not produce methane; methanogens are a different type of bacteria. Methanotrophs can grow aerobically or anaerobically. Under aerobic conditions, they combine oxygen and methane to form formaldehyde. To assimilate carbon, the formaldehyde is then incorporated into organic compounds via the ribulose monophosphate (RuMP) pathway (type I methanotrophs—Gammaproteobacteria) or the serine pathway (type II methanotrophs—Alphaproteobacteria). A type X methanotroph is also in soil. Methane is a potent greenhouse gas, so the role of methanotrophs in the global methane budget is of interest in studies of climate change. The MUFA marker 16:1 ω8c (8-Hexadecenoic acid/cis-8-Palmitoleic acid) is used by MIDI, and it automatically assigns this peak to the methanotroph (type I) category. Other researchers also assign 18:1 ω8c (10-Octadecenoic acid) to type II methanotrophs. If 18:1 ω8c is in the samples, it will appear in the [**WT_wt.xlsx**] master PLFA peak file. Bannert et al. ([2012](#_ENREF_1)) also suggest various other markers that can be used for methanotrophs.

Sulfate-reducing bacteria (Desulfobacter sp.).—This group commonly is not identified in PLFA data, and it is not automatically identified by the MIDI software. Some researchers use cy17:0 and 10Me16:0 markers, but only if high levels of 10Me18:0 are not present ([Kaur et al., 2005](#_ENREF_9)). These peaks are in the master [**WT_wt.xlsx**] file.

Archaea (single-celled prokaryotes).—Archaea are universally distributed in soils and are important contributors to nitrification and NH_4_ oxidation in agricultural and forested soils. PLFAs from archaea are ether-linked, not ester-linked, so they are not in the Buyer-Sasser extraction analysis used by the Soil and Plant Testing Laboratory.

Eukaryotes.—This group includes fungi, algae, nematodes, earthworms, insects, arthropods, and protozoa that are important in soil ecology. Eukaryotes have more complex cell structures than do prokaryotes such as bacteria. The cells of eukaryotes have a membrane-bound nucleus and other membrane-bound organelles. Eukaryotes feed on soil organic matter, soil bacteria, plants, and other eukaryotes. When new residues are added to a soil, the population of bacteria increases. This leads to an increase in bacteria-feeding protozoa and subsequent cascading effects to the food web. The larger soil eukaryotes are also “nature’s tillers.” They help to mix the soil and create channels for water and air in the soil. General eukaryotic markers include PUFAs. The MIDI software automatically assigns peaks (28 possible) to this category.

Protozoa (unicellular eukaryotes).—Protozoa are not assigned by the MIDI software. Some researchers assign PUFA peaks to protozoa, including 20:2 ω6c (Eicosadienoic acid), 20:3 ω6c (Dihomo-gamma-linolenic acid), 20:4 ω6c (Arachidonic acid), and others ([Moore-Kucera and Dick, 2008](#_ENREF_12)). Use the [**WT_wt.xlsx**] master file for these peaks.

Fungi.—Fungi are important for decomposition, especially for recalcitrant organic compounds such as lignin. A wide variety of fungi are in soil. They range from single cell yeasts to some of the largest organisms in the world. Some produce plant diseases. Others live cooperatively with plants, such as saprophytic fungi that break down organic matter to help produce humus and provide plant-available nutrients. Fungi generally prefer more acidic soils than do bacteria, and they tend to prefer the surface soil horizon over subsurface horizons at a greater depth. The MIDI software assigns only 18:2 ω6c (9,12-Octadecadienoic acid/Linoleic acid) to fungi. Other researchers assign multiple peaks to fungi, including 18:1 ω9, 18:2 ω6,9, and 18:3 ω3,6,9 ([Frostegård et al., 2011](#_ENREF_6); [Kaiser et al., 2010](#_ENREF_8); [Klamer and Bååth, 2004](#_ENREF_11)). These additional peaks may or may not be in the data. Assign them to fungi if they are to be used in the analysis. Check the [**WT_wt.xlsx**] file to see if the extra fungi peaks are in the data. Note: The 18:1 ω9c and 18:2 ω6,9c peaks are unique categories in the Fatty Acid Types file [**FT_WT.xlsx**].

Arbuscular mycorrhizae fungi (AMF).—AMF grow in long, thin strands called hyphae, and they form mutually beneficial relationships with most plants. They colonize plant roots and produce growths called arbuscles inside the cells of the roots. Plants provide AMF with carbon (i.e., energy). In return, mycorrhizas provide plants with drought tolerance and increased nutrient uptake of phosphorus (P), nitrogen (N), sulfur (S), zinc (Zn) and copper (Cu). Mycorrhizas also produce glomalin, a protein that protects the hyphae. The glomalin also helps to glue soil particles together, promoting stability of soil aggregates. Mycorrhizal cover crops can increase inoculum for a succeeding crop. Some cover crops, such as buckwheat radishes and other brassicas, are non-mycorrhizal. These crops provide other benefits to soil, but overuse may decrease the population of mycorrhizas. AMF hyphae form lipid material that may be released into the soil. Also, AMF have lipid storage organs called vesicles that contain the fatty acids 18:2 ω6c (commonly used as an indicator of fungal content in PLFA analysis) and 16:1 ω5c (recommended as a biomarker for AMF but is also in bacteria). The MIDI software assigns only 16:1 ω5c to AMF. View the [**WT_wt.xlsx**] file to see if the extra AMF peak is in the data.

Nematodes.—Nematodes currently are not indicated by the MIDI software. They are identified by other methods found in the literature ([Chen et al., 2001](#_ENREF_4); [Ruess et al., 2002](#_ENREF_15)).

**Fatty Acid Types Identified by MIDI Software**

MIDI files [**FT_WT.xlsx and FT_%.xlsx**] include Straight, Branched, MUFA, PUFA, Cyclo, Hydroxy, 10-methyl, DMA, 16:1 ω5c, 18:1 ω9c, 18:2 ω6,9c, and Other.

- 16:1 ω5c—assigned to AMF
- 18:1 ω9c—can be assigned to fungi (but is not by MIDI)
- 18:2 ω6,9c—assigned to fungi
- 10-methyl—redundant with Actinobacteria, except 10Me22:0
- 10Me22:0—assigned to general PLFA category
- DMA—redundant with anaerobic bacteria
- Straight chains (i.e., 12:0, 13:0, 18:0, etc.)—ubiquitous; therefore, assigned to General category
- Branched chains (iso, anteiso)—redundant with G+ bacteria, except 4 iso/anteiso peaks that are assigned to General category (10Methyls are branched but in own category)
- Cyclos—from G- bacteria and represented by cy17:0 ω7c and cy22:0 ω6c (does not include cy19:0 ω7c marker used in stress ratio)
- MUFA—represents all G- bacteria markers, except cyclos, and the methanotroph marker (16:1 ω8c) (fungal MUFA 18:1 ω9c in own category)
- PUFA—represents eukaryotes (fungal PUFA 18:2 ω6,9c in own category; protozoa PUFA markers not categorized separately)
- Hydroxys—not expected in data because they require a harsher extraction method (per Jeff Buyer)

**Examples of Microbial Group, Fatty Acid Type, and “Stress” Indicator Ratios**

Changes in PLFA composition may represent phenotypic plasticity or shifts in the structure of the microbial community. None of these ratios currently are generated by the Sherlock® MIDI software. Calculate the ratios using the appropriate pmol/g [**wt.xlsx**] data file. A few ratios are given in this section, but other ratios are in the literature. Interpretation of these ratios (i.e., the meaning behind changes in the ratios) is speculative and controversial because the cause of the changes is not known ([Frostegård et al., 2011](#_ENREF_6)). Commonly, the cause is inferred from supporting data in the study (e.g., changes in other measured soil properties may correlate with changes in a ratio, which suggests a relationship).

These ratios do not reflect absolute biomass values because no conversion factors from PLFA concentration to actual biomass are applied. The ratios are a biomass index, and changes indicate relative changes in the biomass ratio. Note: MIDI software reports biomass levels as cells per gram, using a conversion factor of 20,000 cells/pmole of PLFA (See their website for more information. However, use of biomass conversion factors is not recommended [[Frostegård and Bååth, 1996](#_ENREF_5)]).

In general, PLFA structural ratios correspond to changes in microbial group data. The specific markers used vary in the literature.

Gram positive (G+)/gram negative (G-) ratio.—The G+ and G- categories are provided in the MIDI output files [**MC_wt.xlsx**], but the ratio is calculated by the analyst. Research on this ratio is contradictory. Studies suggest that some G+ bacteria may be more robust due to the ability to form spores, which allows them to withstand less hospitable conditions than can G- bacteria. Other studies suggest the reverse is true, which alludes to the enhanced cyclo-fatty acid content in the membrane of G- bacteria. Either way, a shift in the ratio may indicate stressful conditions such as low oxygen, suboptimal pH or water content, low nutrient supply, heavy metal contamination, etc., ([Guckert et al., 1986](#_ENREF_7); [Keynan and Sandler, 1983](#_ENREF_10); [Pennanen et al., 1996](#_ENREF_13)).

Fungal/bacterial ratio.—Fungi are important in the decomposition of organic matter, soil aggregation, and other soil functions. A decrease in this ratio may reflect a soil disturbance, application of fungicides, or other environmental changes. Fungi are represented by 18:2 ω6 and possibly a few other markers (See Fungi section.). Frostegård and Baath ([1996](#_ENREF_5)) suggested that a fungal/bacterial biomass ratio could be estimated by using the PLFA 18:2 ω6,9 as a measure of fungal biomass and the sum of 13 bacteria-specific PLFAs as a measure of bacterial biomass. Selected bacterial markers vary slightly in the literature. Calculate the bacteria category by summing selected bacterial marker peaks from the [**WT_wt.xlsx**] file. Examples of selected general bacterial PLFA peaks are below.

- i14:0, i15:0, a15:0, 15:0, i16:0, 10Me16:0, i17:0, a17:0, cy17:0, 17:0, br18, 10Me17:0, 18:1 ω7, 10Me18:0, cy19:0 ([Frostegård et al., 2011](#_ENREF_6))
- i15:0, a15:0, 15:0, 16:0, 16:1 ω9, 16:1 ω7, i17:0, 17:0, cy17:0, 18:1 ω7, cy19:0 ([Kaur et al., 2005](#_ENREF_9))
- Jeff Buyer uses 18:2 ω6c for fungi and sums G+ and G- for bacteria (Buyer and Sasser, [2012](#_ENREF_3)).
- Sum all the bacteria categories provided by MIDI (G+, G-, methanotrophs, anaerobes, and actinos).

Metabolic status and stress ratio.—G- bacteria produce high levels of monounsaturated fatty acids (e.g., 16:1 ω7 and 18:1 ω7) during active metabolism, or the growth phase. Much of the composition of unsaturated fatty acids is converted to cyclopropane fatty acids (e.g., 17:0 cyclopropane and 19:0 cyclopropane) when metabolism and cell division slow down due to a shortage of nutrition or other stress (pH, temperature, tillage, starvation, metal toxicity, pesticides, etc.). Thus, (cy17:0 + cy19:0) / (16:1 ω7c + 18:1 ω7c) is used as a stress indicator. Higher ratio values suggest greater stress ([Kaur et al., 2005](#_ENREF_9); [Moore-Kucera and Dick, 2008](#_ENREF_12); [Petersen and Klug, 1994](#_ENREF_14); [Villanueva et al., 2004](#_ENREF_17)).

MUFA/PUFA ratio.—MUFAs are produced by AMF, G- bacteria, methanotrophs (I and II), and fungi. PUFAs are produced by microeukaryotes, including fungi and protozoa markers. Higher MUFA than PUFA suggests a shift to G- bacteria.

Saturated/MUFA ratio.—The saturated part equals straight (general eukaryotes and prokaryotes) + branched (G+ bacteria) + 10Methyls (actinobacteria). The monounsaturated part equals MUFA (potentially plus 16:1 ω5c + 18:1 ω9c). Bossio and Scow ([1998](#_ENREF_2)) do not include branched saturated PLFAs in this ratio.

Cis/trans ratios (e.g., c/t ratio of 16:1 ω7).—Isomerization of cis unsaturated fatty acids (16:1 ω7c, 18:1 ω7c) to trans unsaturated fatty acids (16:1 ω7t, 18:1 ω7t) can be induced by environmental stress ([Guckert et al., 1986](#_ENREF_7); [Kaur et al., 2005](#_ENREF_9); [Petersen and Klug, 1994](#_ENREF_14)). The MIDI software does not differentiate between cis and trans isomers, so these ratios cannot be calculated with output from the Soil and Plant Testing Laboratory.

The literature provides more information on these ratios and others.

# **References**

Bannert, A., C. Bogen, J. Esperchutz, A. Koubova, F. Buegger, D. Fischer, V. Radl, R. FuB, A. Chronakova, D. Elhottova, M. Simek, and M. Schloter. 2012. Anaerobic oxidation of methane in grassland soils used for cattle husbandry. Biogeosciences 9, 3891-3899.

Bossio, D. A., and K. M. Scow. 1998. Impacts of carbon and flooding on soil microbial communities: Phospholipid fatty acid profiles and substrate utilization patterns. Microbial Ecology 35, 265-278.

Buyer, J. S., and M. Sasser. 2012. High throughput phospholipid fatty acid analysis of soils. Applied Soil Ecology 61, pages 127-130.

Bremmer, J.M., and C.S. Mulvaney. 1982. Nitrogen–Total. p. 595–624. *In* A.L.

Page, R.H. Miller, and D.R. Keeney (eds.) Methods of soil analysis. Part 2.

Chemical and microbiological properties. 2nd ed. Agron. Monogr. 9. ASA

and SSSA, Madison, WI.

Chen, J., H. Ferris, K. M. Scow, and K. J. Graham. 2001. Fatty acid composition and dynamics of selected fungal-feeding nematodes and fungi. Comparative Biochemistry and Physiology Part B: Biochemistry and Molecular Biology 130(2), 135-144.

Frostegård, A., and E. Bååth. 1996. The use of phospholipid fatty acid analysis to estimate bacterial and fungal biomass in soil. Biology and Fertility of Soils 22, 59-65.

Frostegård, A., A. Tunlid, and E. Bååth. 2011. Use and misuse of PLFA measurements in soils. Soil Biology and Biochemistry 43(8), 1621-1625.

Guckert, J. B., M. A. Hood, and D. C. White. 1986. Phospholipid ester-linked fatty acid profile changes dueing nutrient deprivation of Vibrio cholerae: Increases in the trans/cis ratio and proportions of cyclopropyl fatty acids. Applied and Environmental Microbiology 52(4), 794-801.

Kaiser, C., A. Frank, B. Wild, M. Koranda, and A. Richter. 2010. Negligible contribution from roots to soil-borne phospholipid fatty acid fungal biomarkers 18:2 ω6,9 and 18:1 ω9. Soil Biology and Biochemistry 42(9), 1650-1652.

Kaur, A., A. Chaudhary, A. Kaur, R. Choudhary, and R. Kaushik. 2005. Phospholipid fatty acid - A bioindicator of environment monitoring and assessment in soil ecosystem. Current Science 89(7), 1103-1112.

Keynan, A., and N. Sandler. 1983. Spore research in historical perspective. In The Bacterial Spore. A. Hurst and G. W. Gould, editors. Volume 2. Academic Press, New York, NY. Pages 1-48.

Klamer, M., and E. Bååth. 2004. Estimation of conversion factors for fungal biomass determination in compost using ergosterol and PLFA 18:2 ω6,9. Soil Biology and Biochemistry 36(1), 57-65.

Moore-Kucera, J., and R. P. Dick. 2008. PLFA profiling of microbial community structure and seasonal shifts in soils of a Douglas-fir chronosequence. Microbial Ecology 55, 500-511.

Pennanen, T., A. Frostegård, H. Fritze, and E. Bååth. 1996. Phospholipid fatty acid Composition and heavy metal tolerance of soil microbial communities along two heavy metal-polluted gradients in coniferous forests. Applied and Environmental Microbiology 62(2), 420-428.

Patterson, G.T. 1993. Collection and preparation of soil samples: Site description. p. 1–4. In Martin R. Carter (ed.) Soil sampling and methods of analysis. Can. Soc. Soil Sci. Lewis Publ., Boca Raton, FL.

Petersen, S. O., and M. J. Klug.1994. Effects of sieving, storage, and incubation temperature on the phospholipid fatty acid profile of a soil microbial community. Applied and Environmental Microbiology 60(7), 2,421-2,430.

Rabenhorst, M.C., L.T. West, and L.P. Wilding. 1991. Genesis of calcic and petrocalcic horizons in soils over carbonate rocks. p. 61–74. *In* W.D. Nettleton (ed.) Occurrence, characteristics, and genesis of carbonate, gypsum, and silica accumulations in soils. Soil Sci. Soc. Am. Spec. Publ. No. 26. ASA and SSSA, Madison, WI.

Ruess, L., M. M. Häggblom, E. J. Garcı́a Zapata, and J. Dighton. 2002. Fatty acids of fungi and nematodes—possible biomarkers in the soil food chain? Soil Biology and Biochemistry. 34(6), 745-756.

Soil Survey Staff. 2014. Kellogg Soil Survey Laboratory Methods Manual. Soil Survey Investigations Report No. 42, Version 5.0. R. Burt and Soil Survey Staff (ed.). U.S. Department of Agriculture, Natural Resources Conservation Service.

Tunlid, A., and D. C. White. 1992. Biochemical analysis of biomass, community structure, nutritional status, and metabolic activity of microbial communities in soil. In Soil Biochemistry. G. Stotzky and J. M. Bollag, editors. Volume 7. Marcel Dekker, Inc., New York. Pages 229-262.

Villanueva, L., A. Navarette, J. Urmeneta, D. C. White, and R. Guerrero. 2004. Combined phospholipid biomarker-16S rRNA gene denaturing gradient gel electrophoresis analysis of bacterial diversity and physiological status in an intertidal microbial mat. Applied and Environmental Microbiology 70(11), 6,920-6,926.

Zelles, L. 1999. Fatty acid patterns of phospholipids and lipopolysaccharides in the characterisation of microbial communities in soil: A review. Biology and Fertility of Soils 29(2), 111-129.

Zelles, L., Q. Y. Bai, T. Beck, and F. Beese. 1992. Signature fatty acids in phospholipids and lipopolysaccharides as indicators of microbial biomass and community structure in agricultural soils. Soil Biology and Biochemistry 24(4), 317-323.

Zelles, L., Q. Y. Bai, R. Rackwitz, D. Chadwick, and F. Beese. 1995. Determination of phospholipid- and lipopolysaccharide-derived fatty acids as an estimate of microbial biomass and community structures in soils. Biology and Fertility of Soils 19(2-3), 115-123.
